# Supplementary material for: Histone Deacetylase Inhibitory Activity and Antiproliferative Potential of New [6]-Shogaol Derivatives
Source: Molecules. 2022 May 22;27(10):3332. doi: 10.3390/molecules27103332 (PMC9144829; doi:10.3390/molecules27103332)
Supplement: Supplementary file 1 [file molecules-27-03332-s001.zip › molecules-1719646-supplementary.pdf]

Histone Deacetylase Inhibitory Activity and Antiproliferative Potential of New [6]-Shogaol Derivatives

Chanokbhorn Phaosiri [1], Chavi Yenjai [1], Thanaset Senawong [2], Gulsiri Senawong [2], Somprasong Saenglee [3], La-or Somsakeesit [4], Pakit Kumboonma [5*]

[1] Natural Products Research Unit, Center of Excellence for Innovation in Chemistry, Ministry of Higher Education, Science, Research and Innovation (Implementation Unit-IU, Khon Kaen University), Department of Chemistry, Faculty of Science, Khon Kaen University, Khon Kaen, 40002 Thailand

[2] Natural Products Research Unit, Department of Biochemistry, Faculty of Science, Khon Kaen University, Khon Kaen, 40002 Thailand

[3] Ban Dong Subdistrict Administration Organization, Ubolratana District, Khon Kaen, 40250 Thailand

[4] Department of Chemistry, Faculty of Engineering, Rajamangala University of Technology Isan, Khon Kaen, 40000 Thailand

[5] Department of Applied Chemistry, Faculty of Science and Liberal Arts, Rajamangala University of Technology Isan, Nakhon Ratchasima, 30000 Thailand

* To whom correspondence should be addressed. e-mail: pakit.ku@rmuti.ac.th

Supporting Information

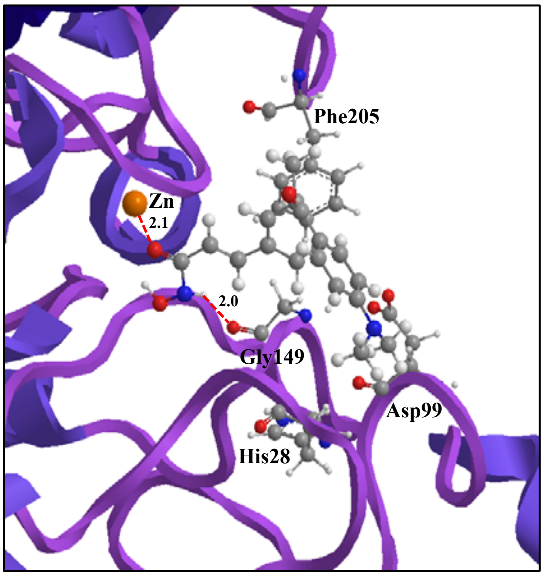

Figure S1. The interaction mode of TSA in the active site of HDAC1.

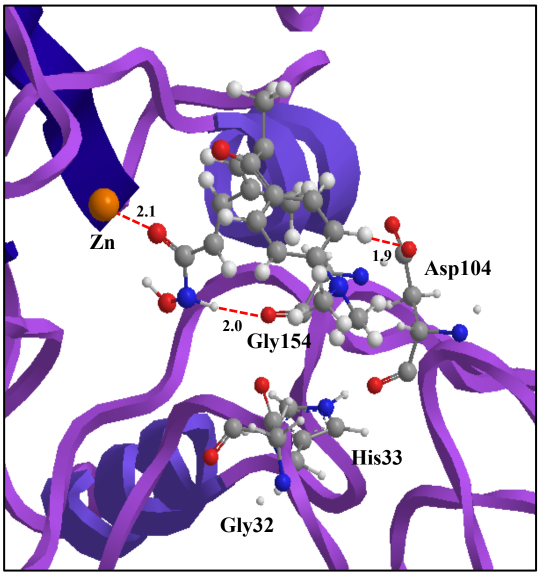

Figure S2. The interaction mode of TSA in the active site of HDAC2.

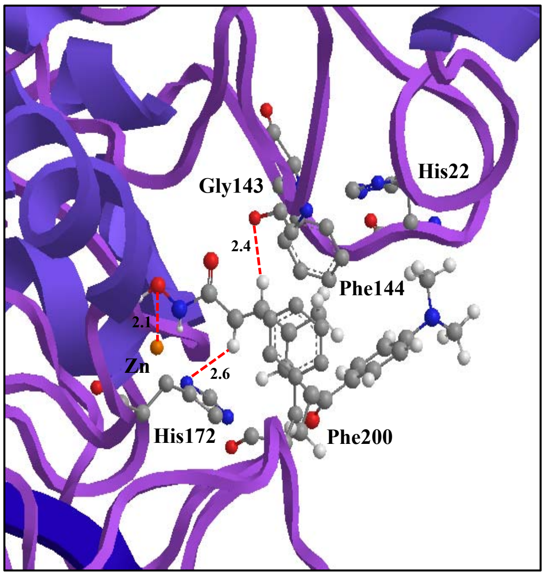

Figure S3. The interaction mode of TSA in the active site of HDAC3.

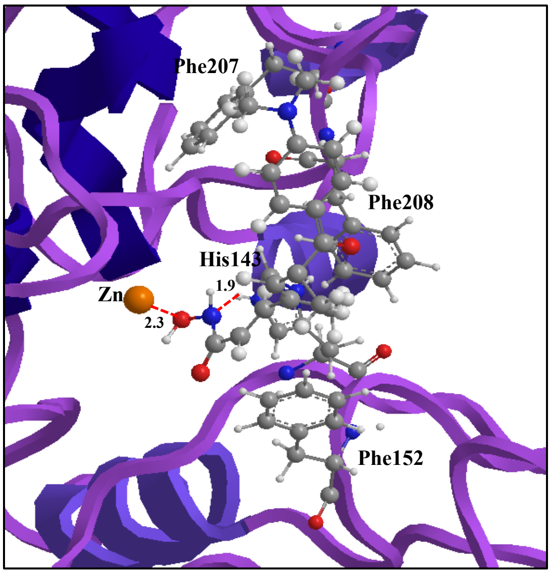

Figure S4. The interaction mode of TSA in the active site of HDAC8.

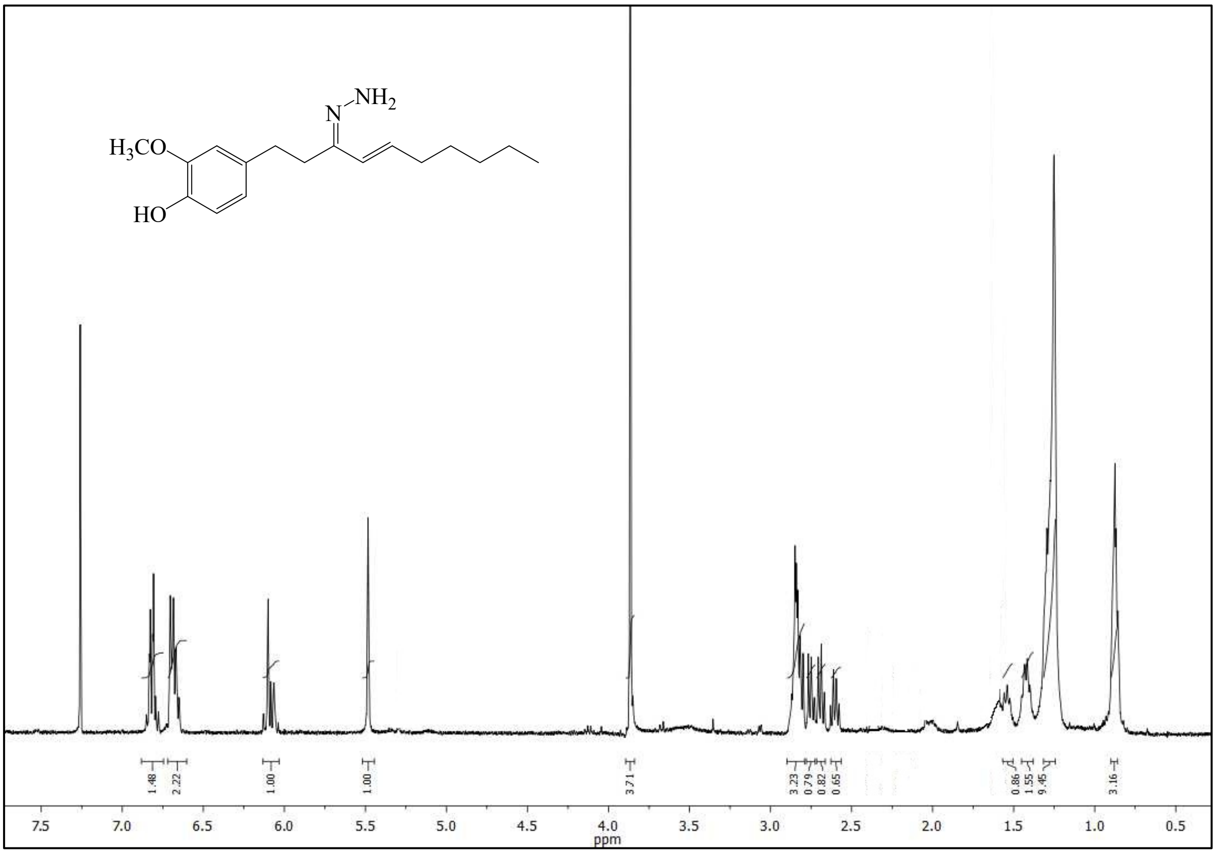

Figure S5. ^{1}H NMR spectrum of **4a**.

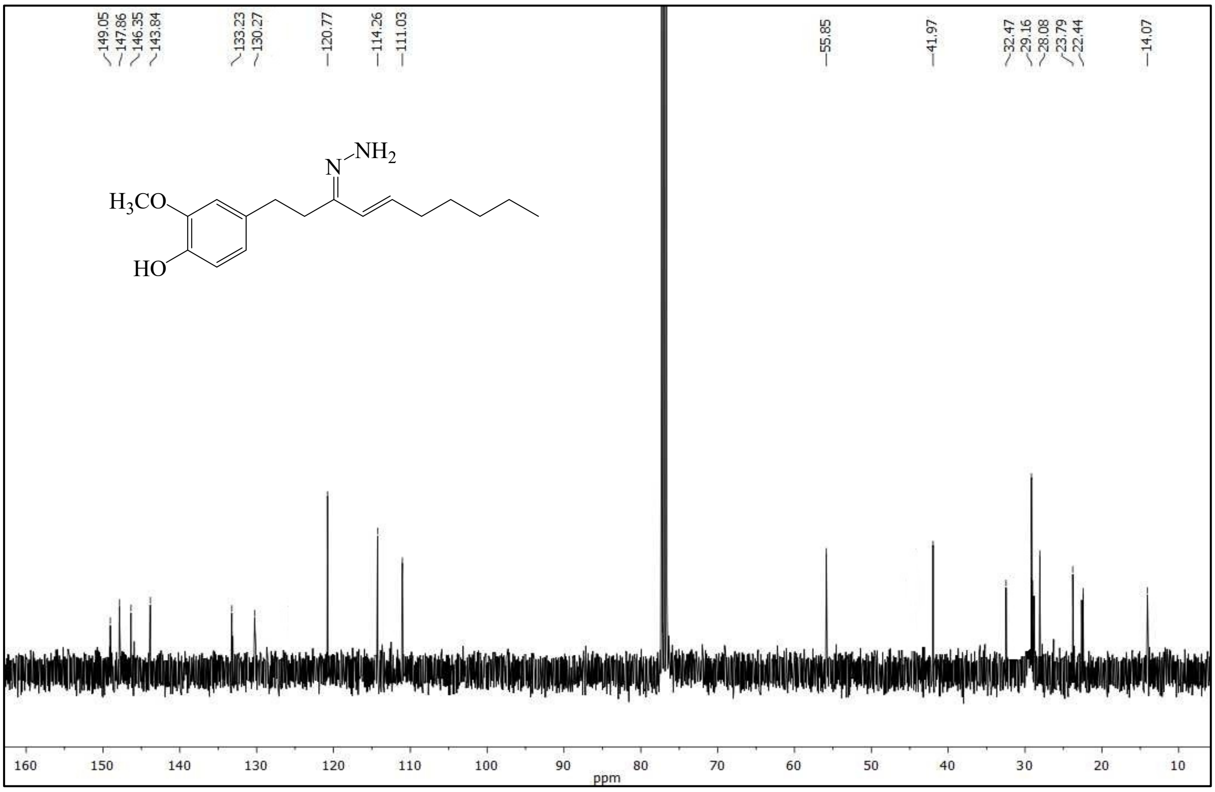

Figure S6. ^{13}C NMR spectrum of **4a**.

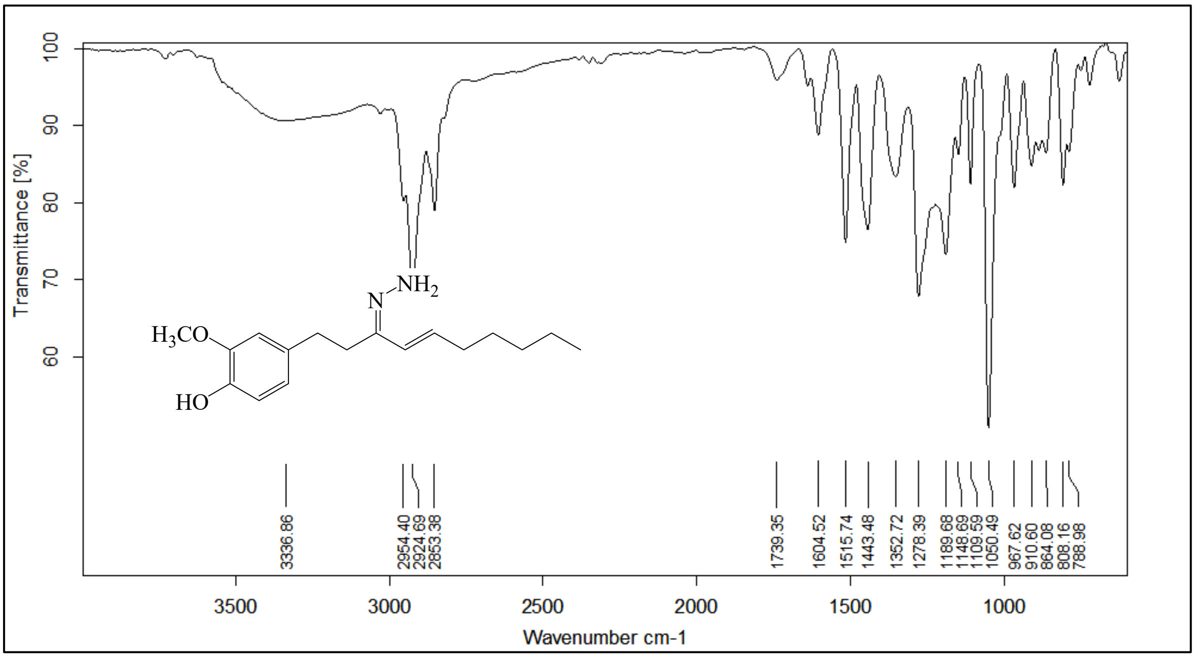

Figure S7.IR spectrum of **4a**.

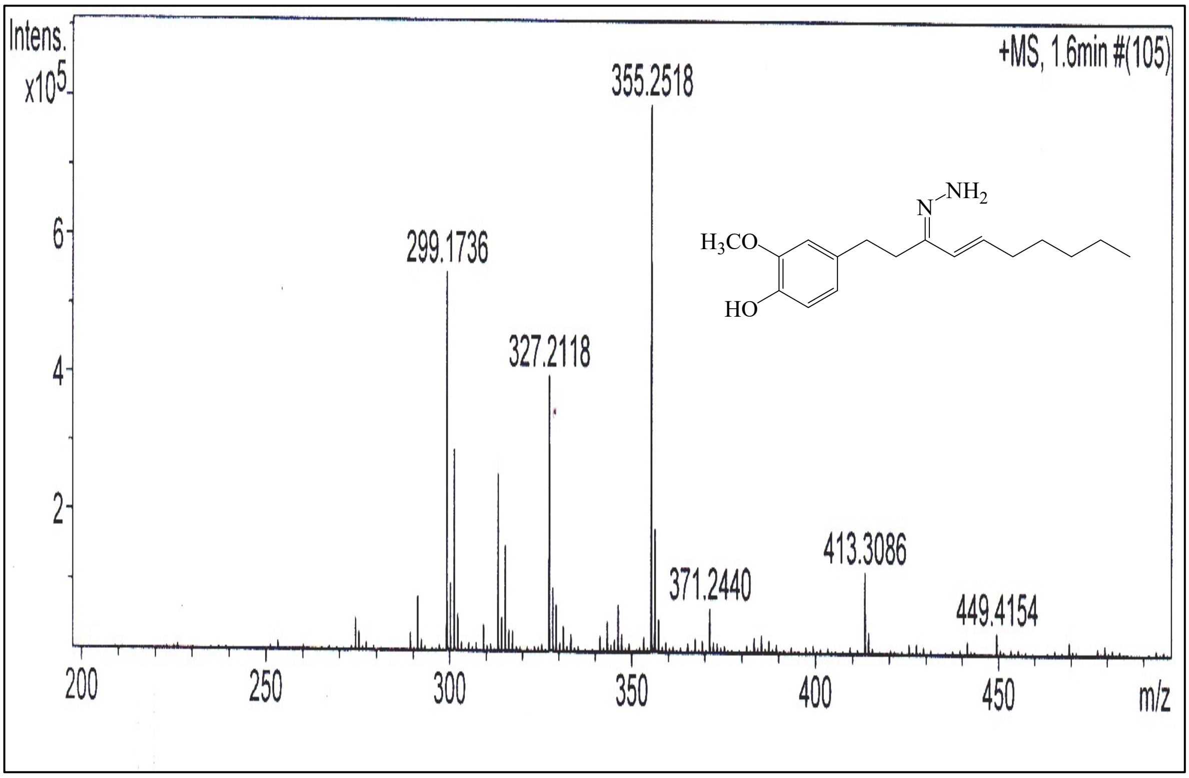

Figure S8. Mass spectrum of **4a.**

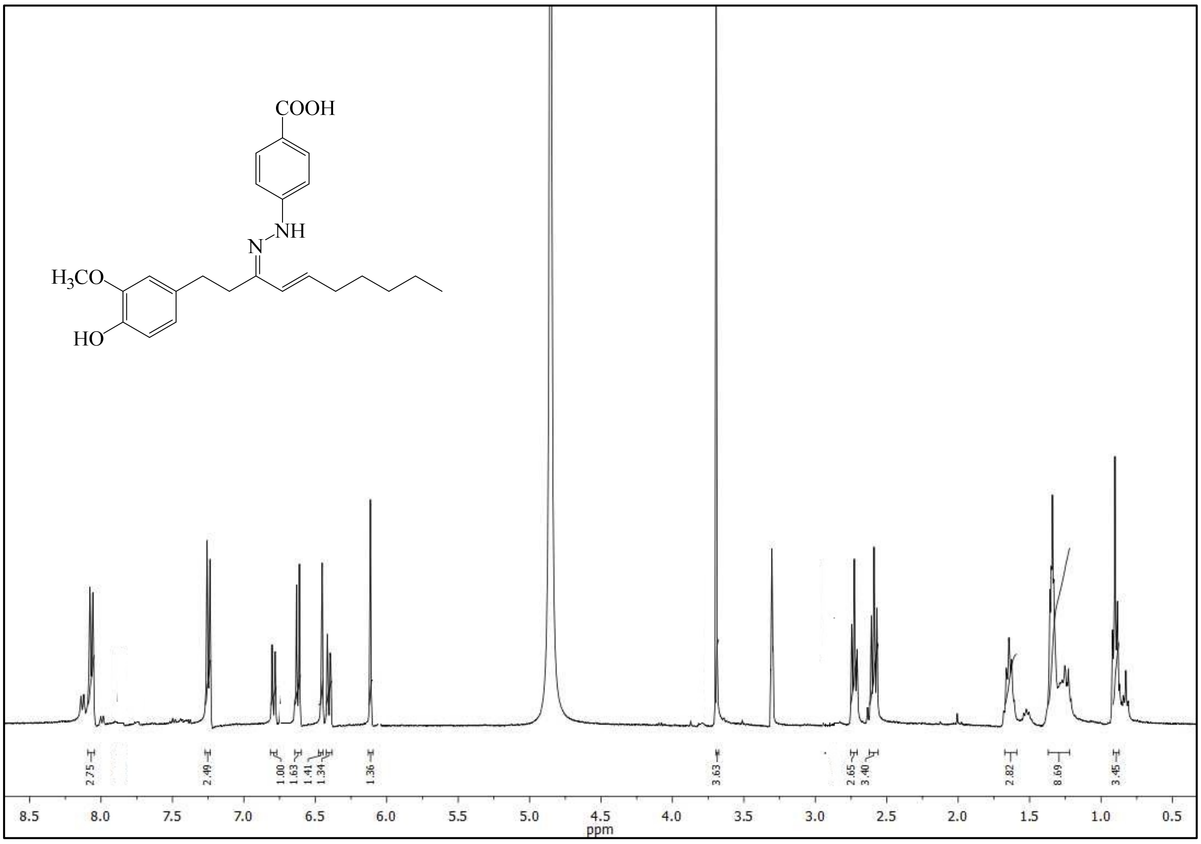

Figure S9. ^{1}H NMR spectrum of **4b**.

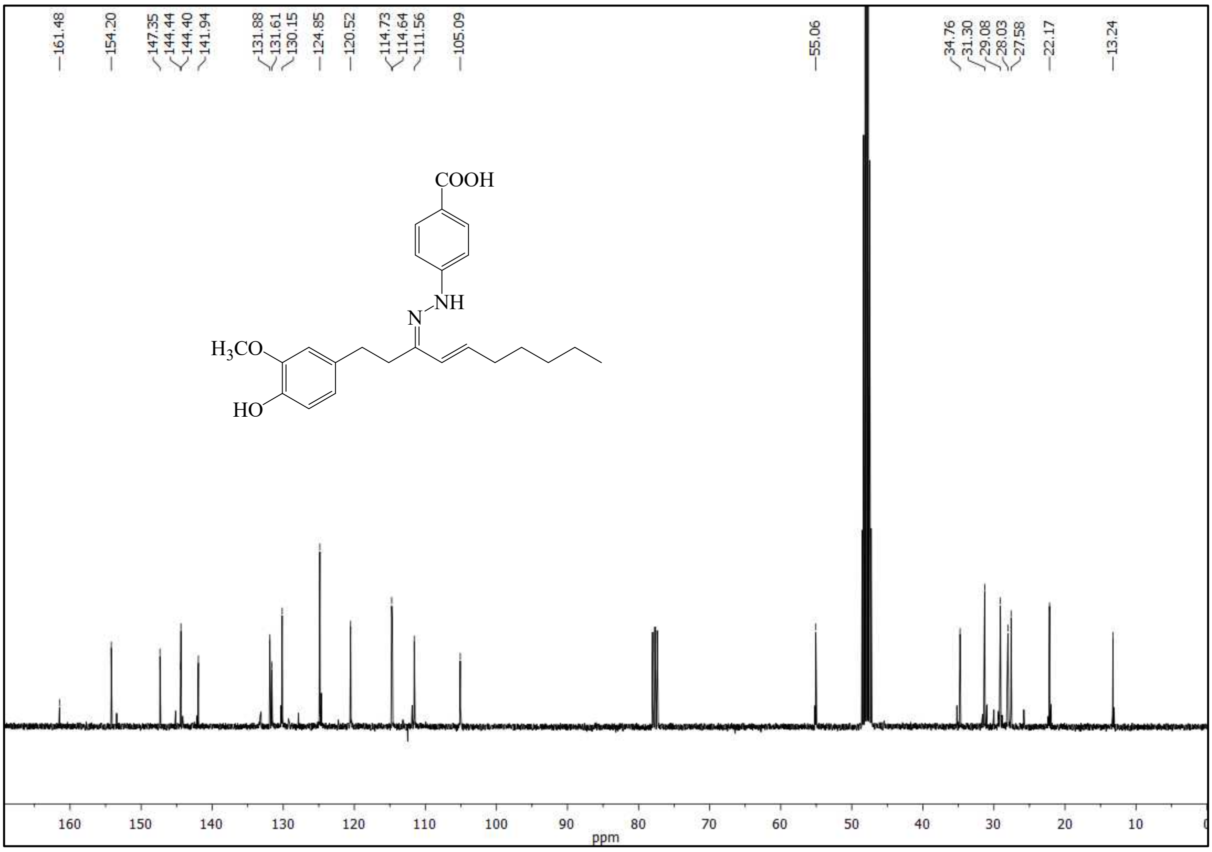

Figure S10. ^{13}C NMR spectrum of **4b**.

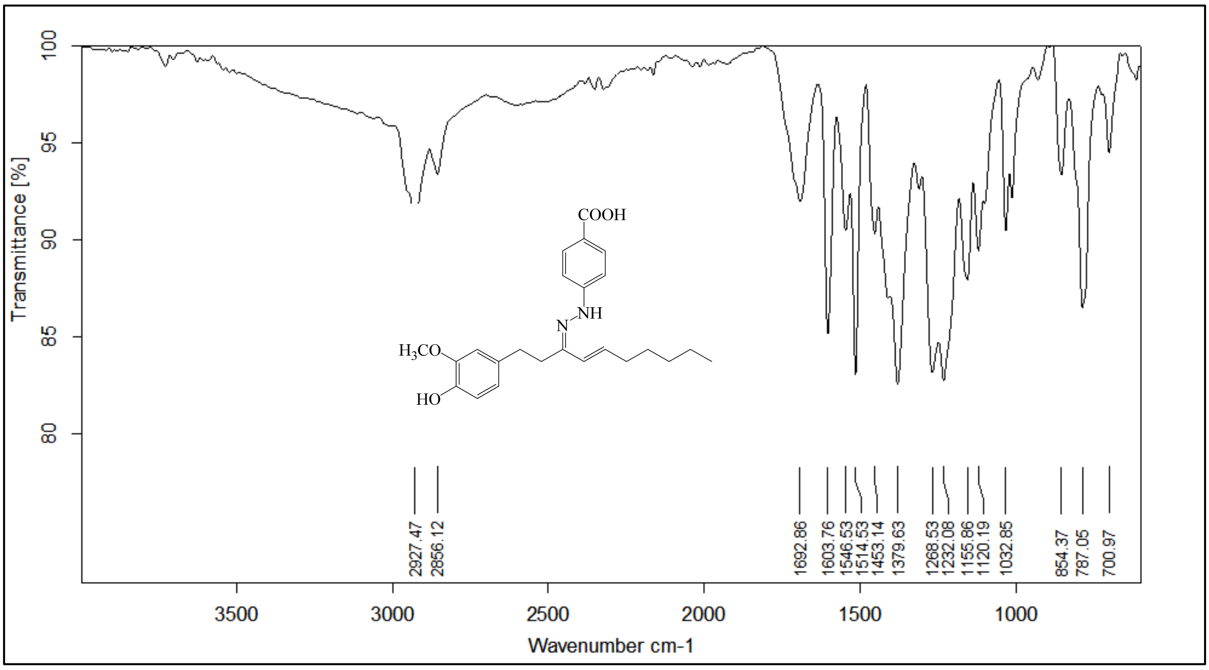

Figure S11. IR spectrum of **4b**.

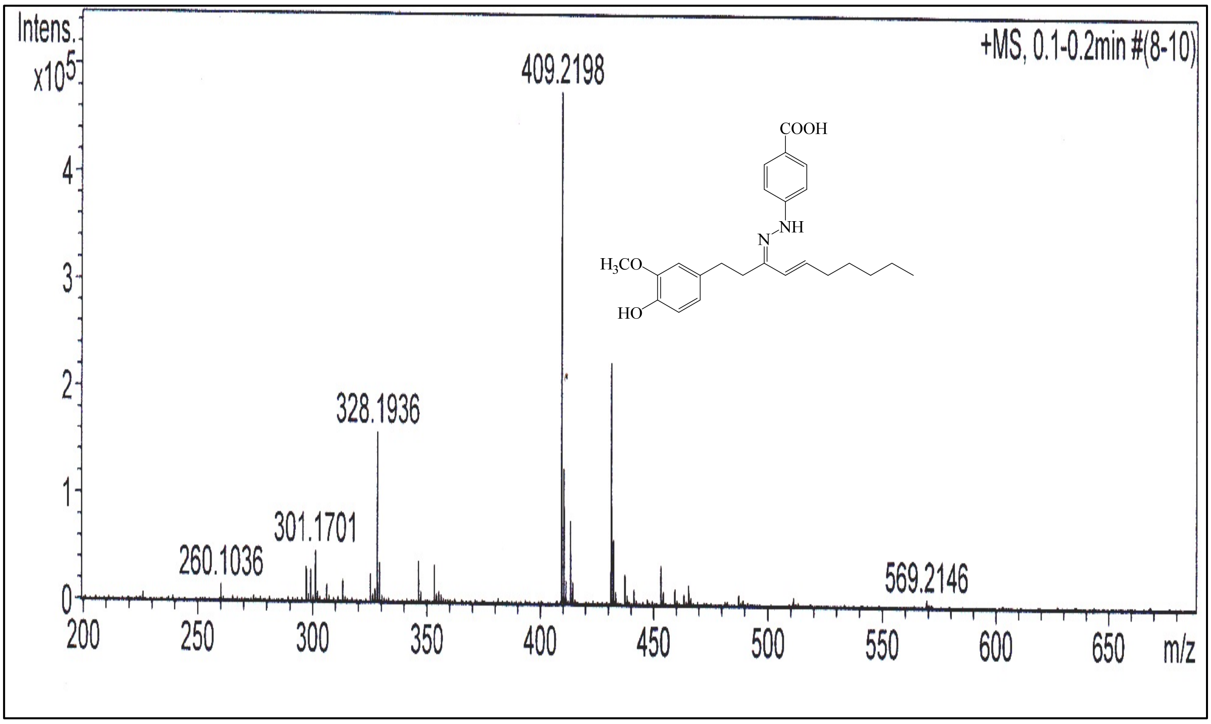

Figure S12. Mass spectrum of **4b**.

#

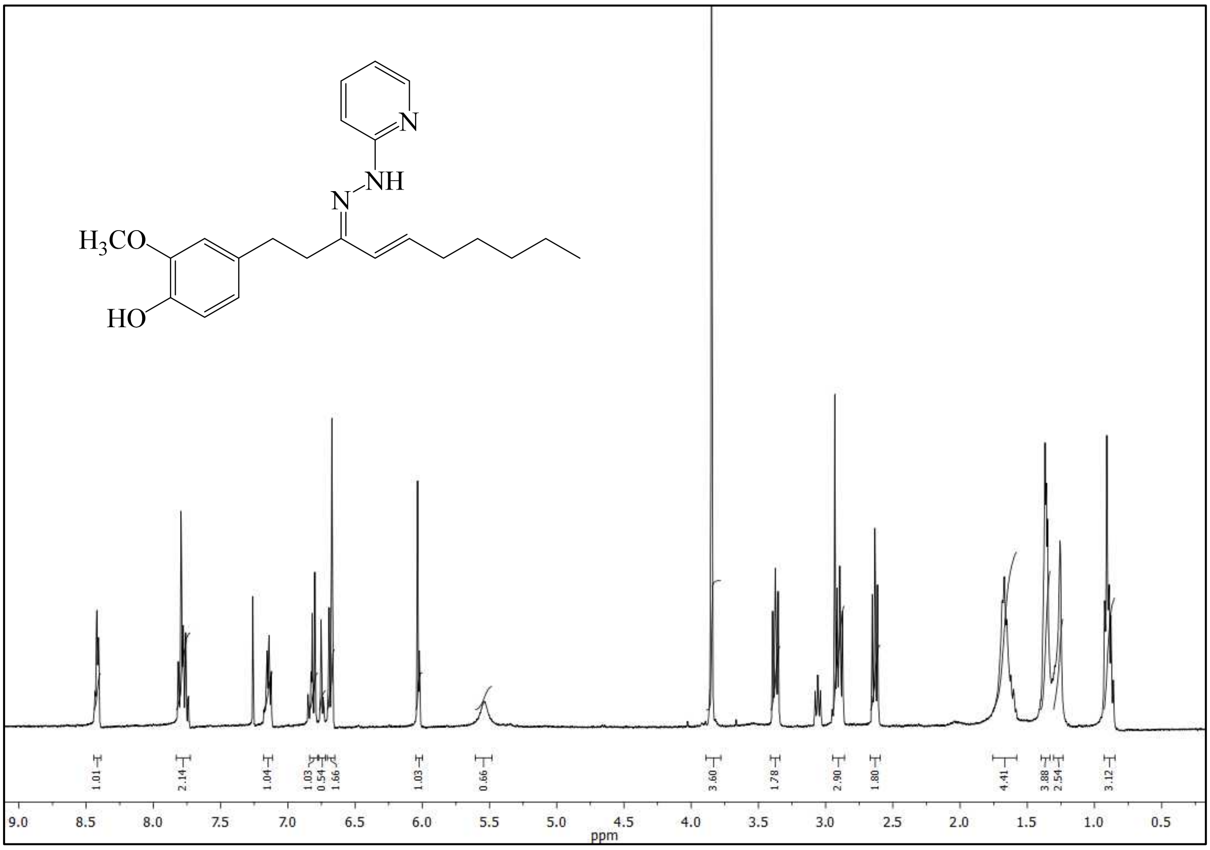

Figure S13. ^{1}H NMR spectrum of **4c.**

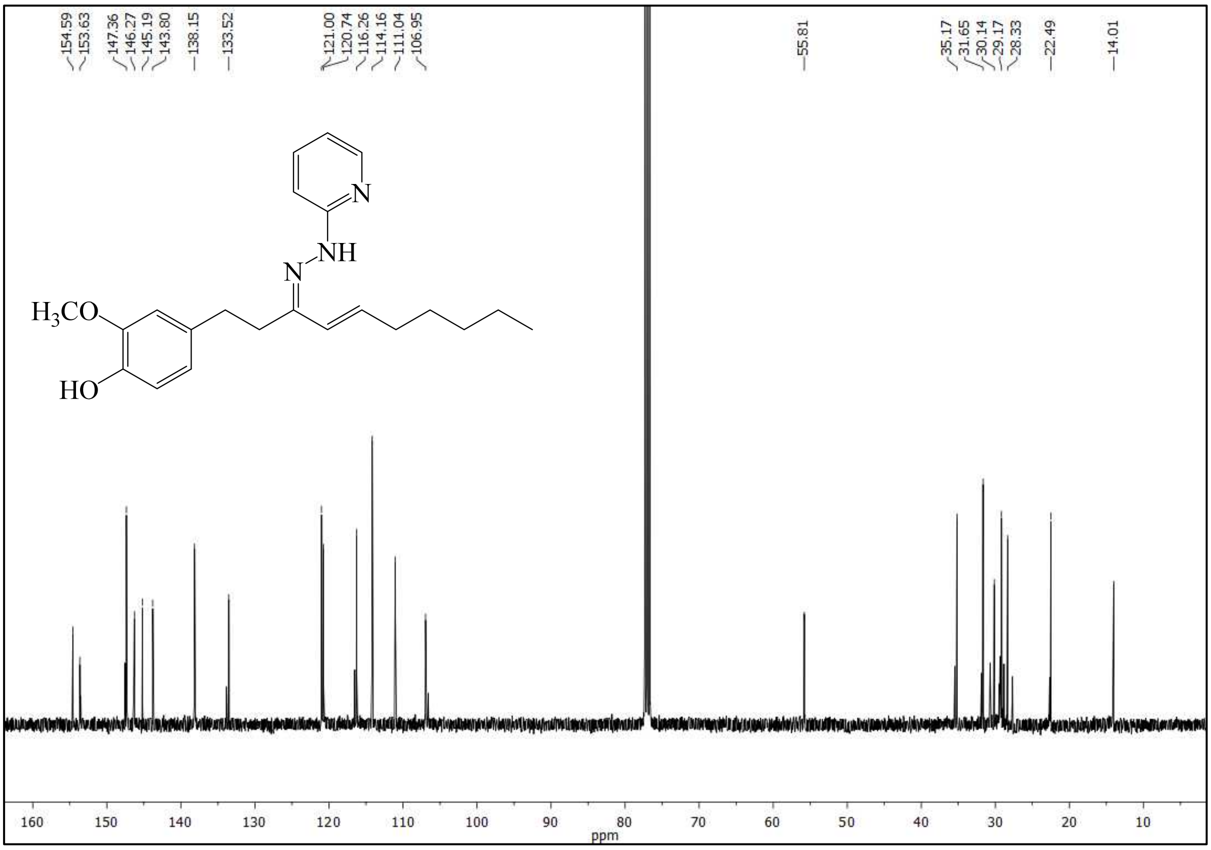

Figure S14. ^{13}C NMR spectrum of **4c**.

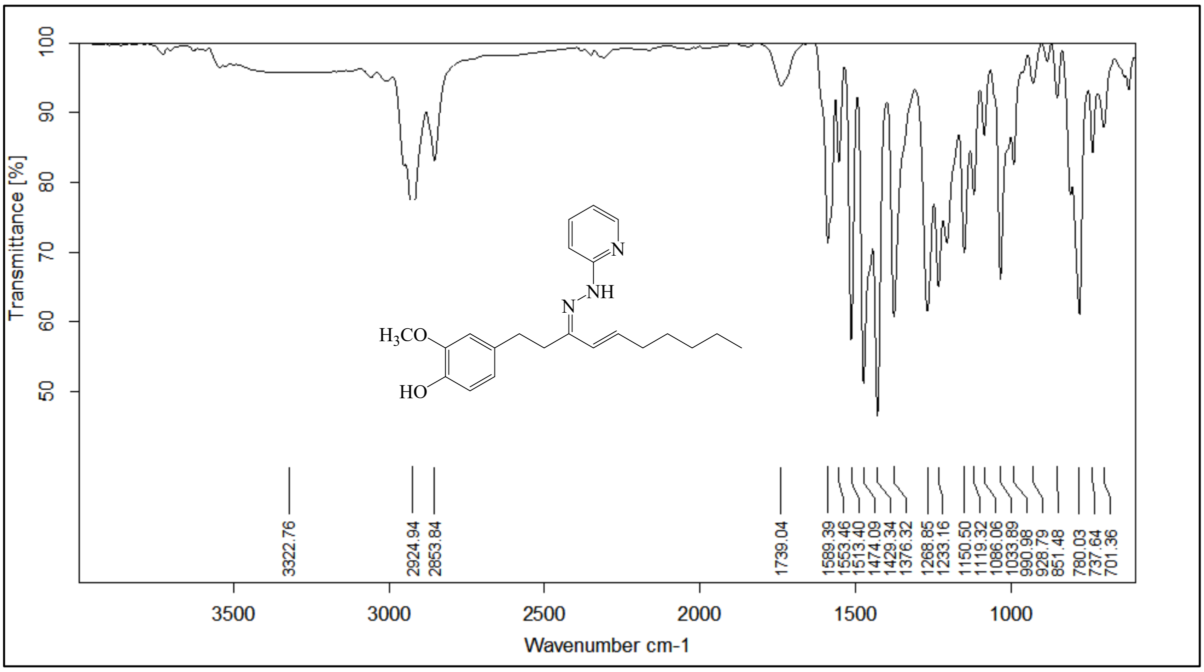

Figure S15. IR spectrum of **4c**.

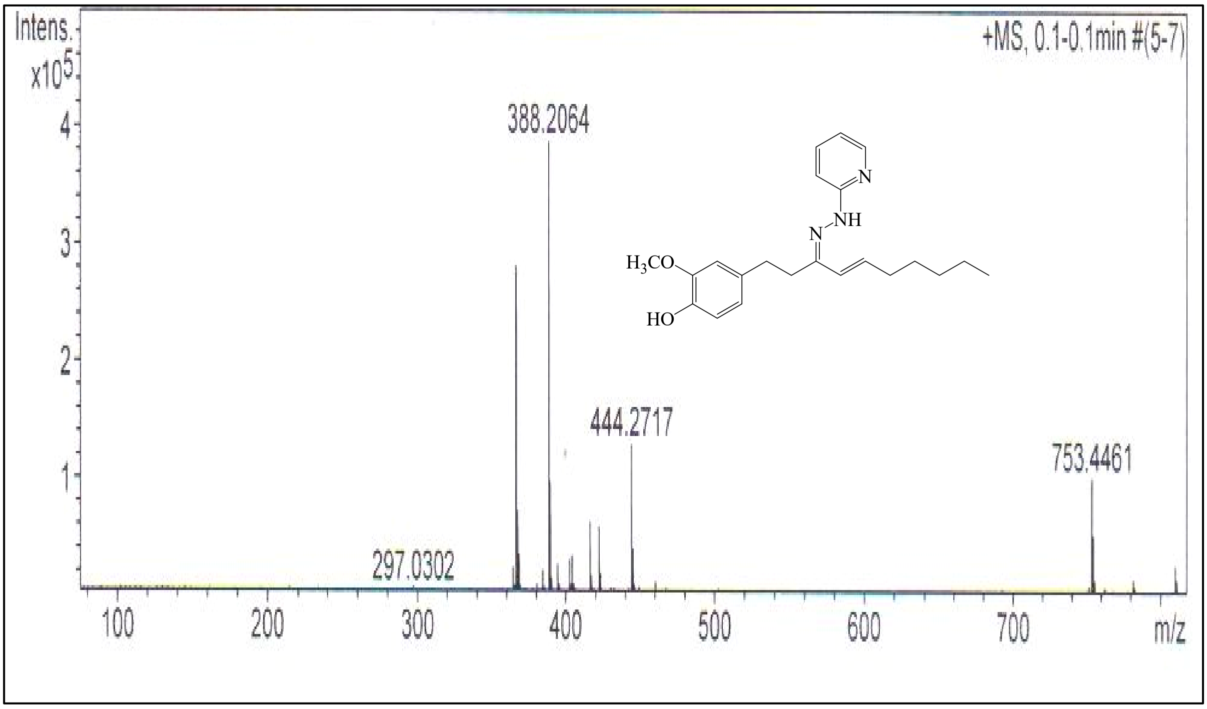

Figure S16. Mass spectrum of **4c**.

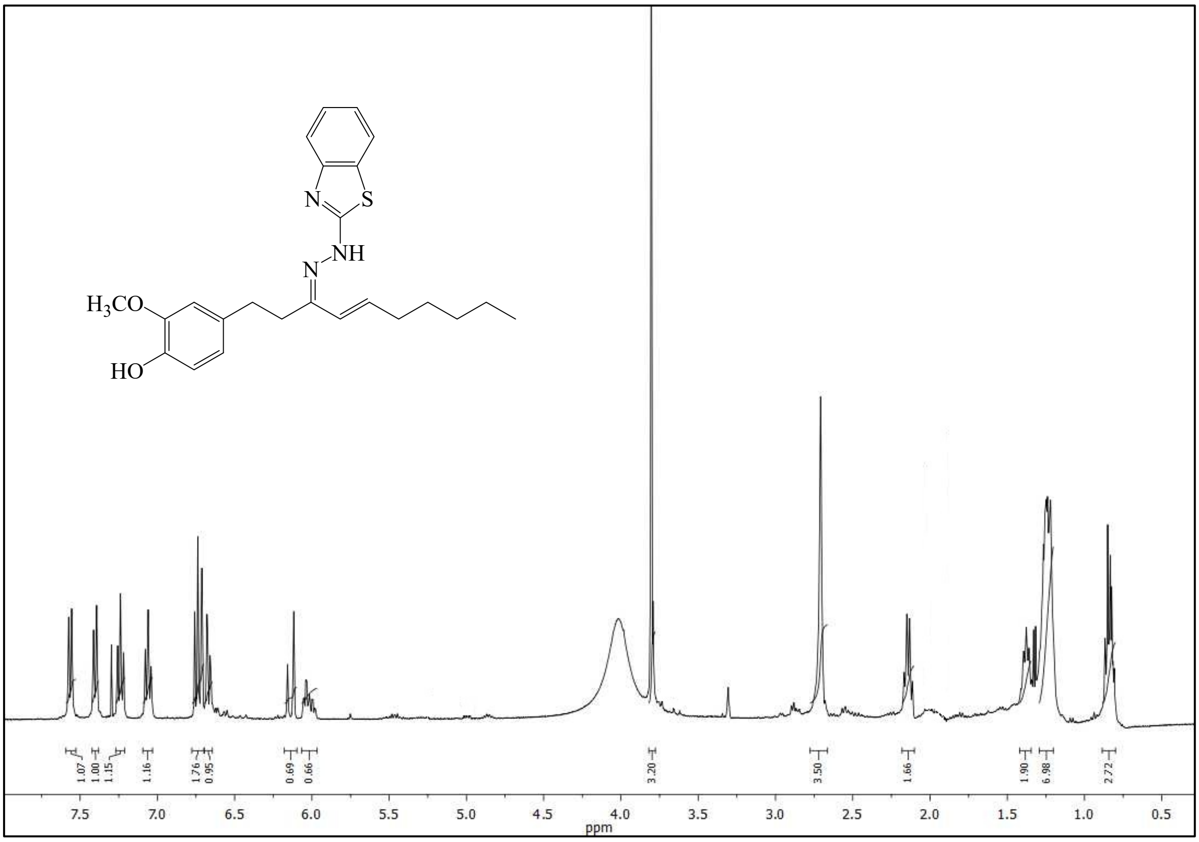

Figure S17. ^{1}H NMR spectrum of **4d**.

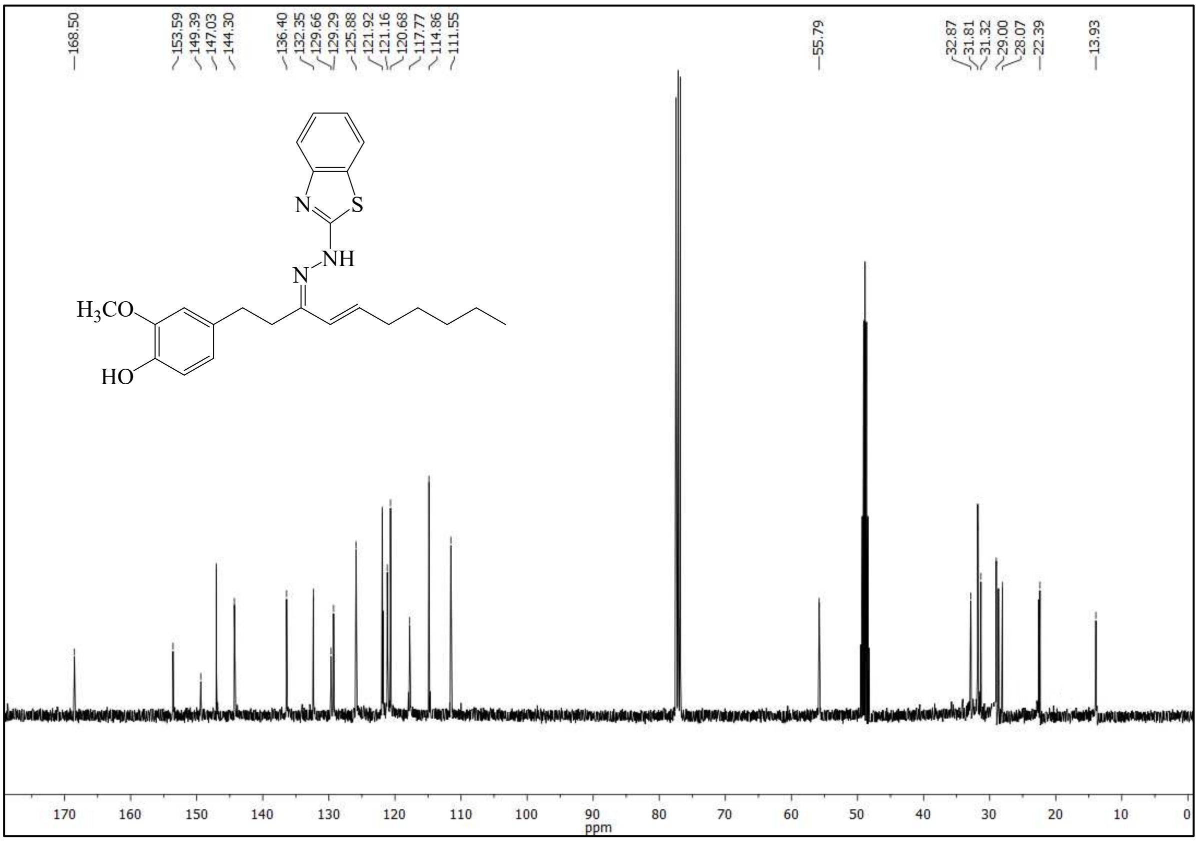

Figure S18. ^{13}C NMR spectrum of **4d**.

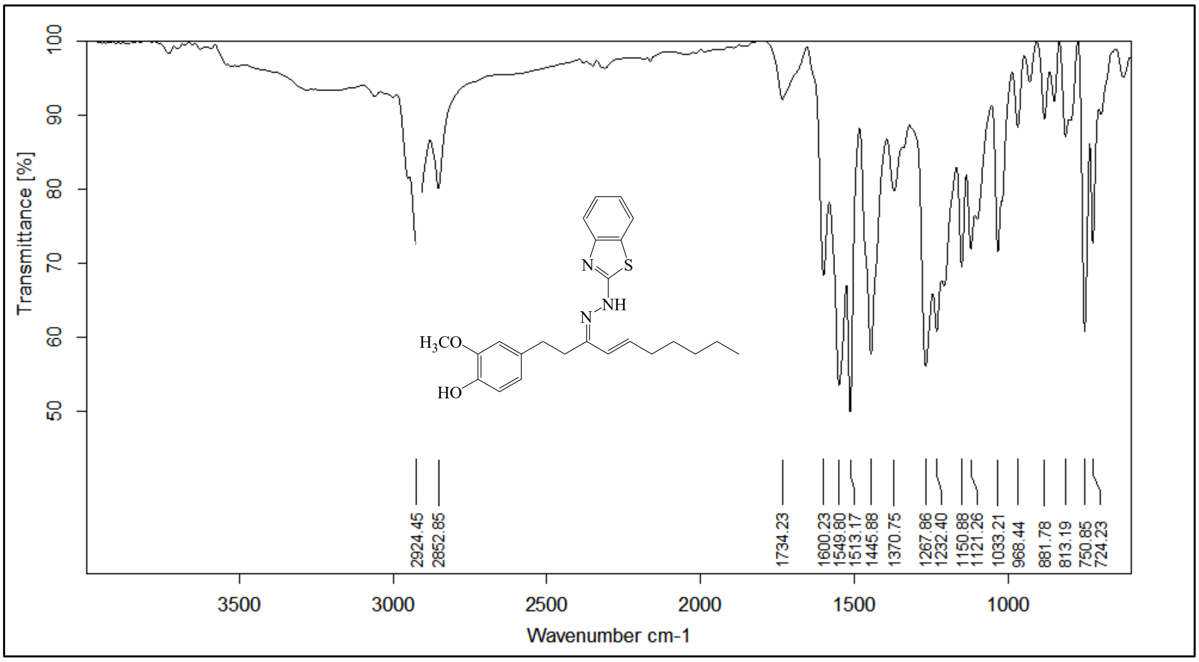

Figure S19. IR spectrum of **4d**.

#

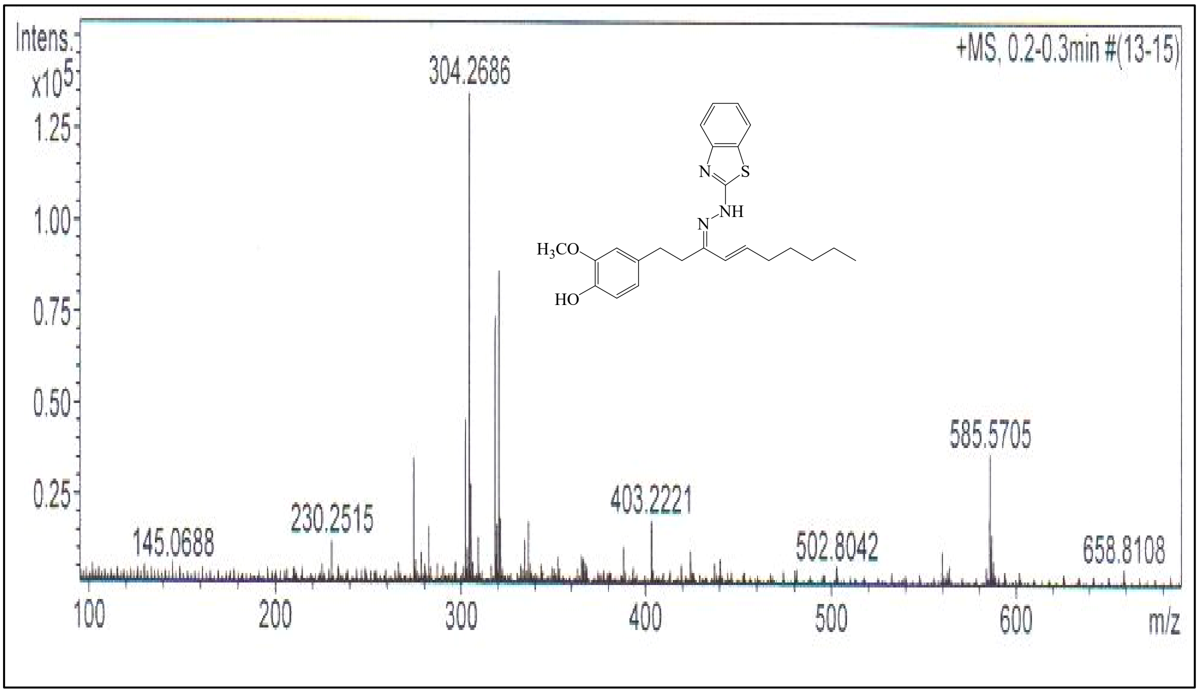

Figure S20. Mass spectrum of **4d**.

#

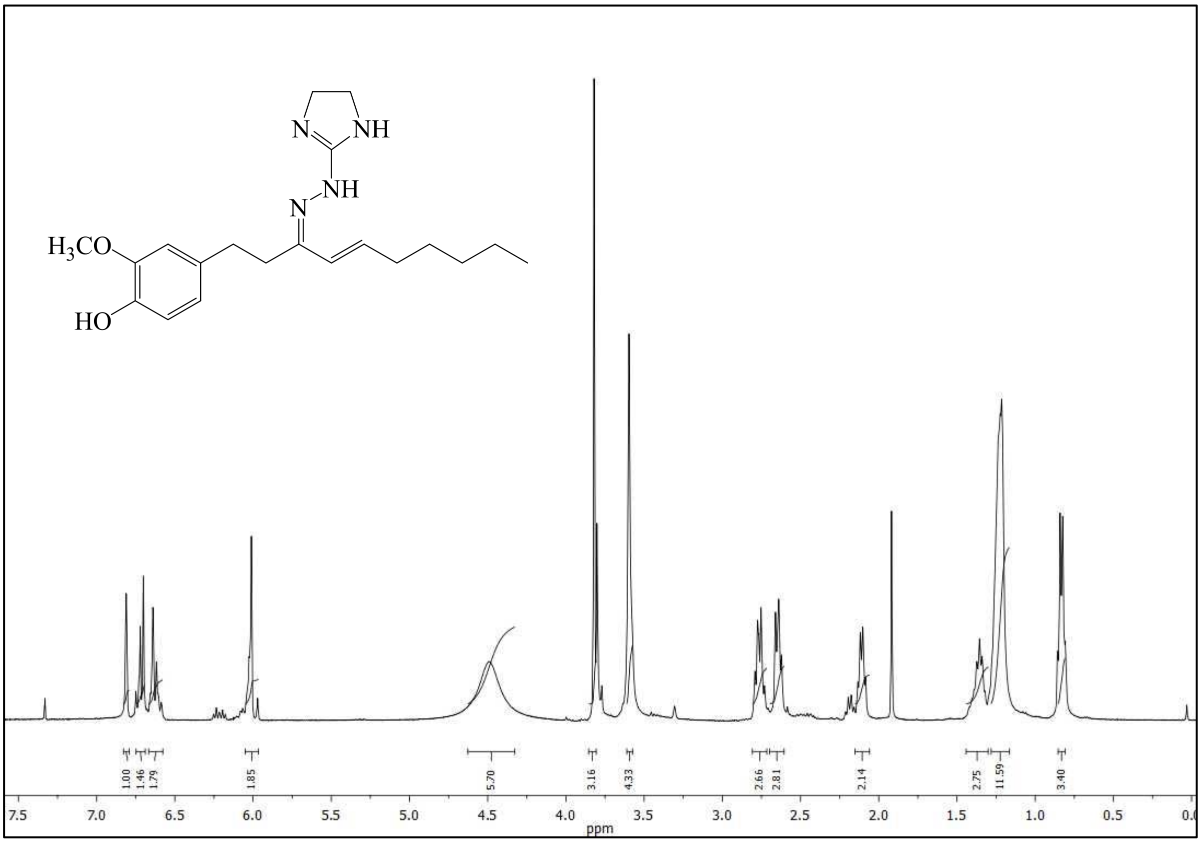

Figure S21. ^{1}H NMR spectrum of **4e**.

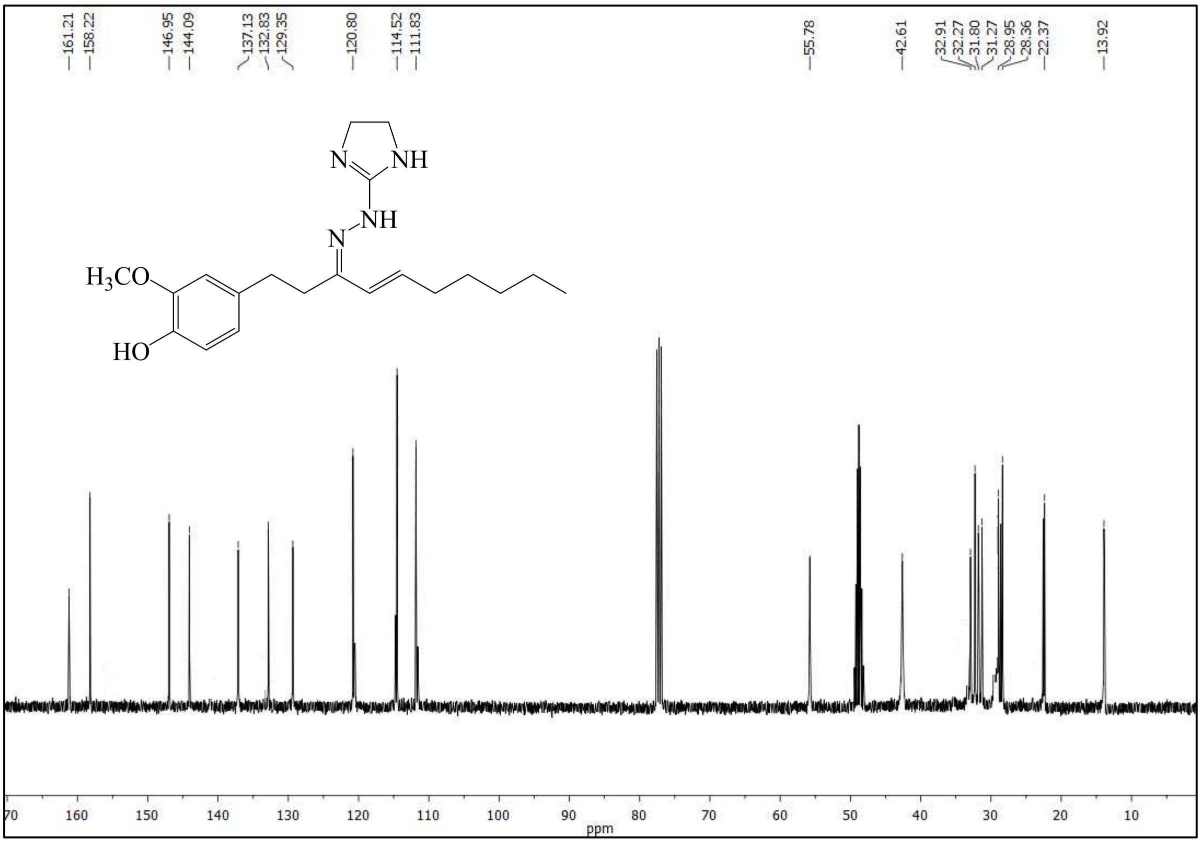

Figure S22. ^{13}C NMR spectrum of **4e**.

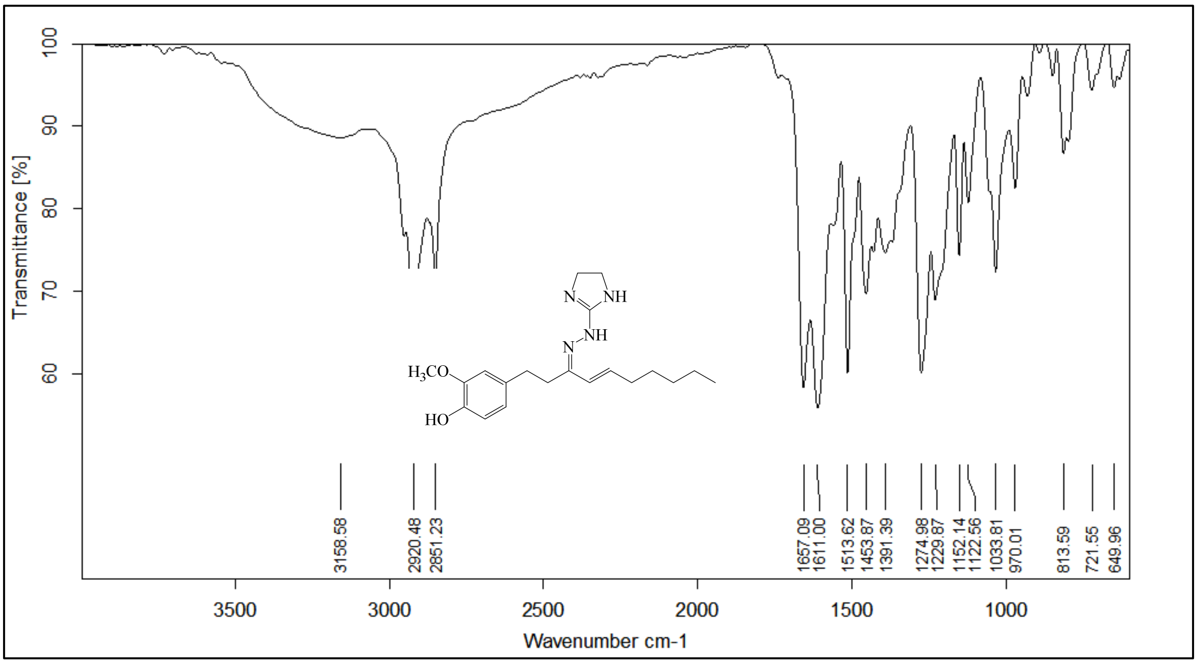

Figure S23. IR spectrum of **4e.**

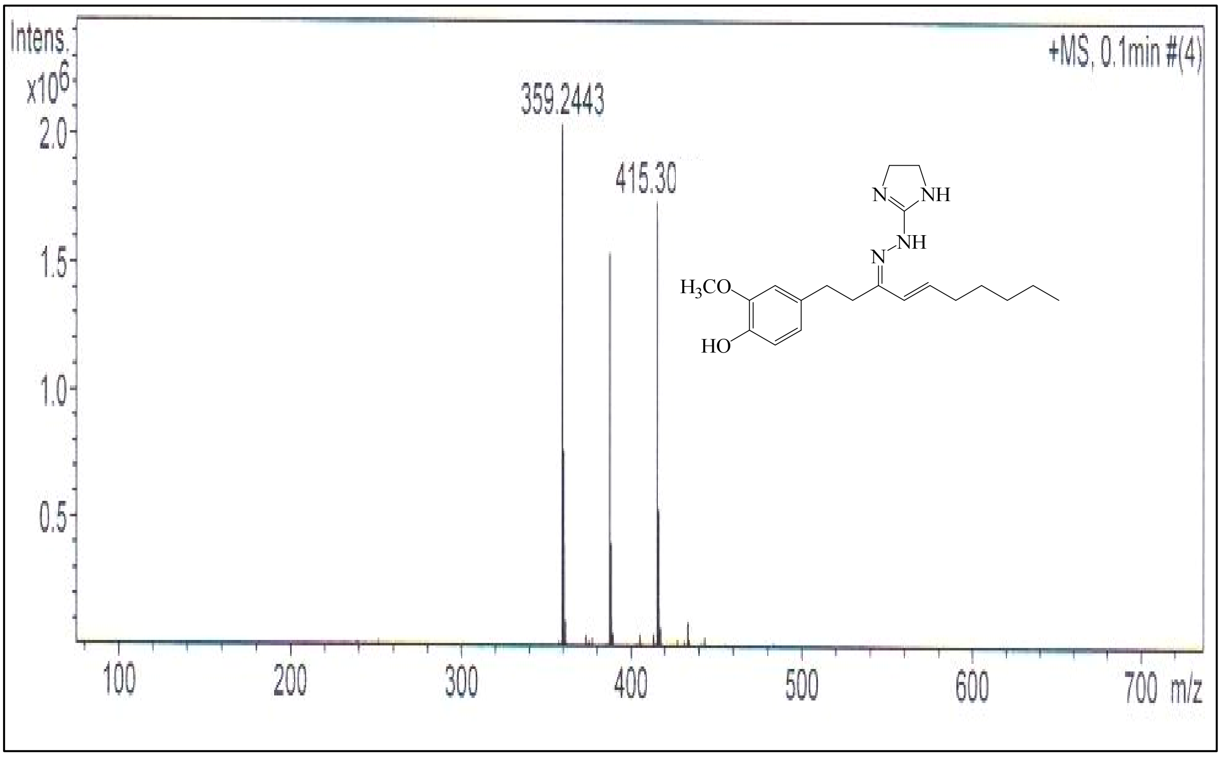

Figure S24. Mass spectrum of **4e**.

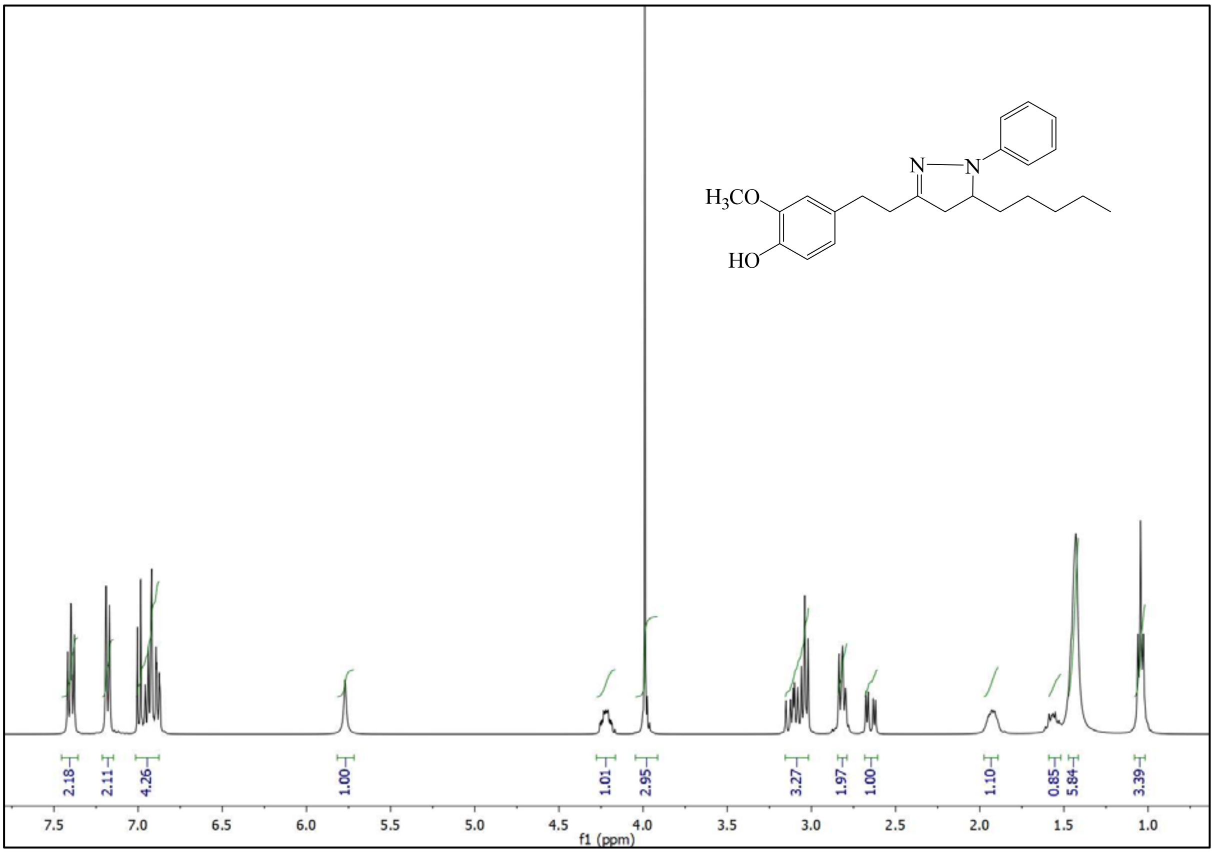

Figure S25. ^{1}H NMR spectrum of **5a**.

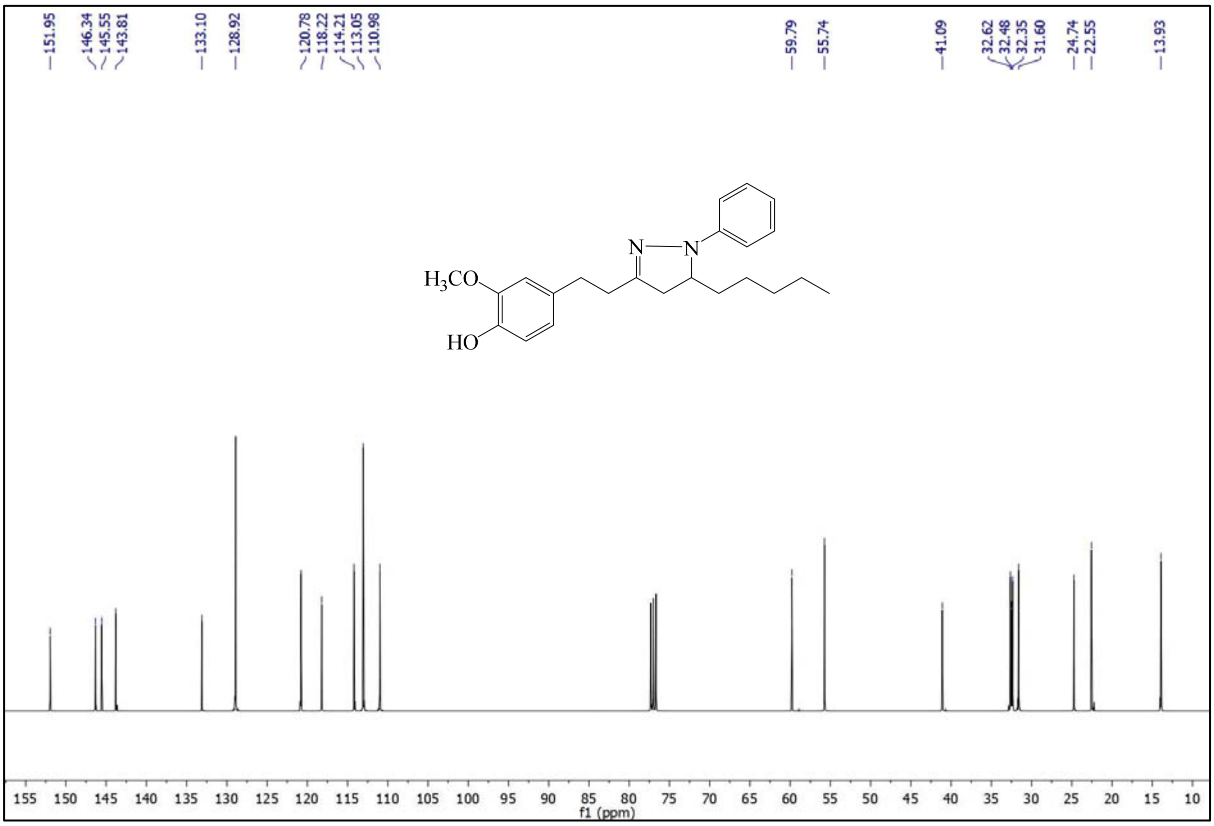

Figure S26. ^{13}C NMR spectrum of **5a.**

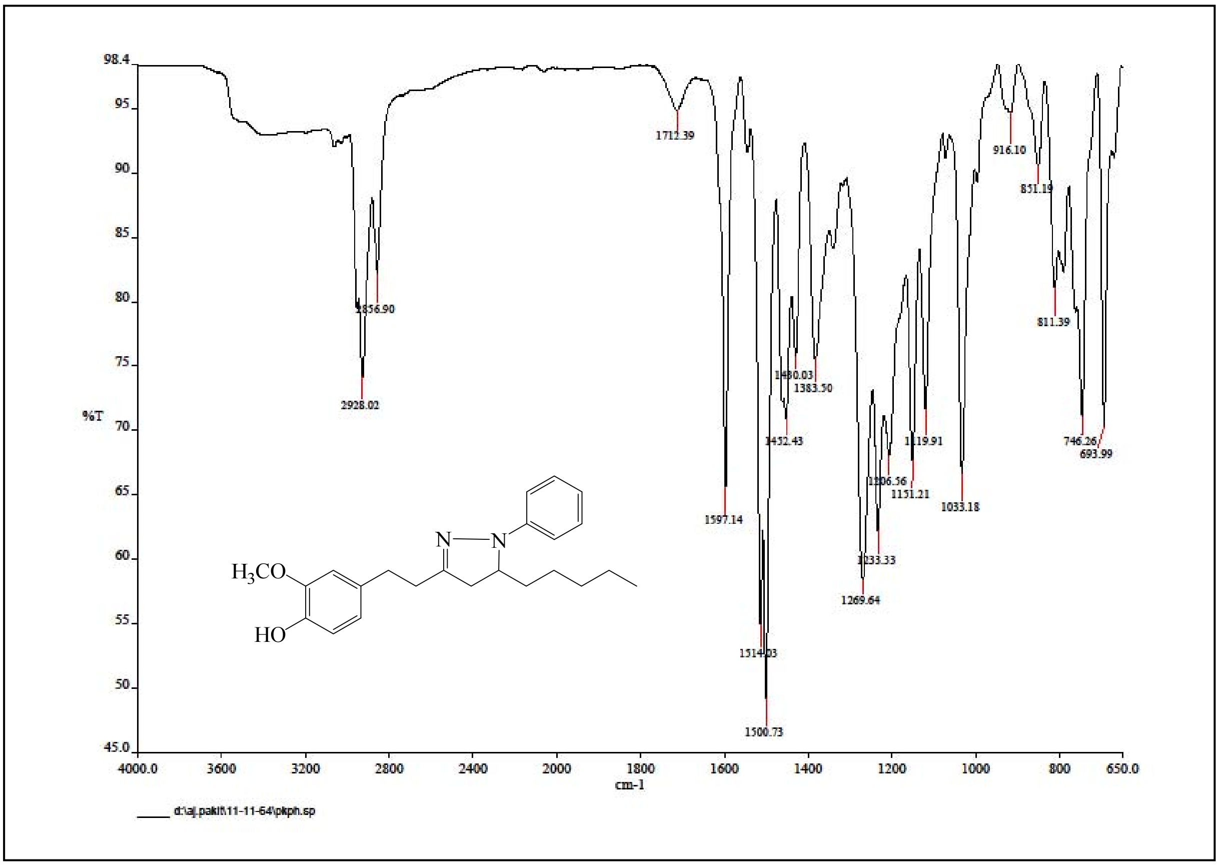

Figure S27. IR spectrum of **5a.**

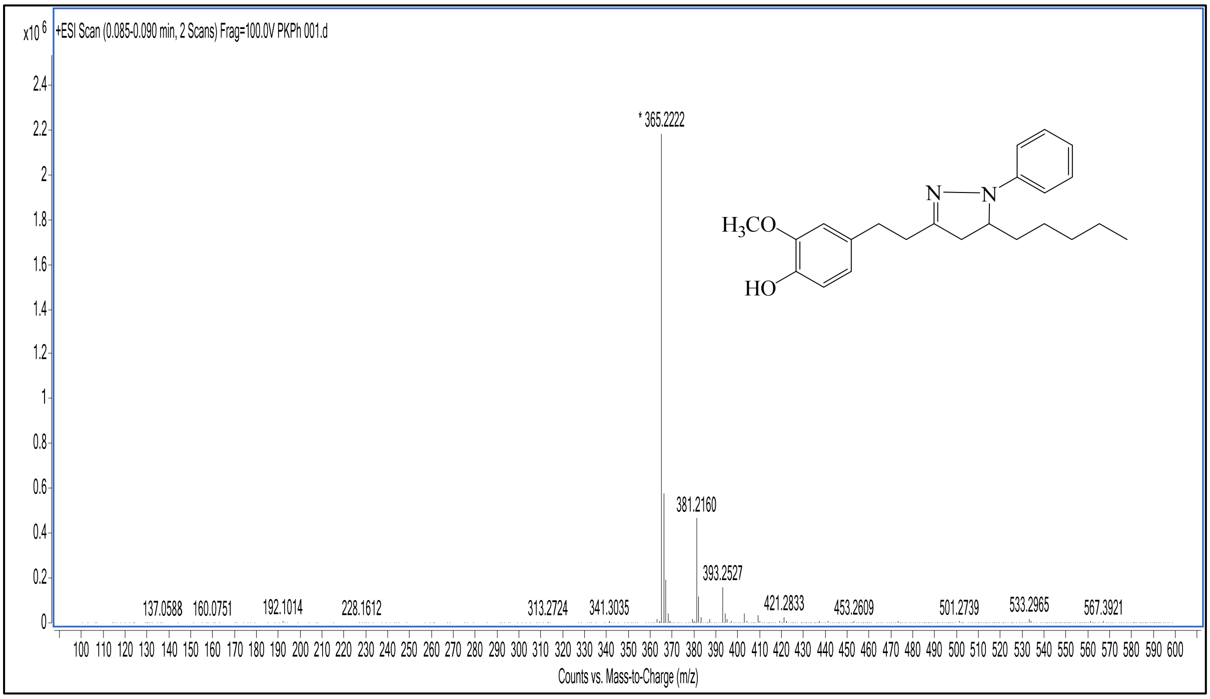

Figure S28. Mass spectrum of **5a**.

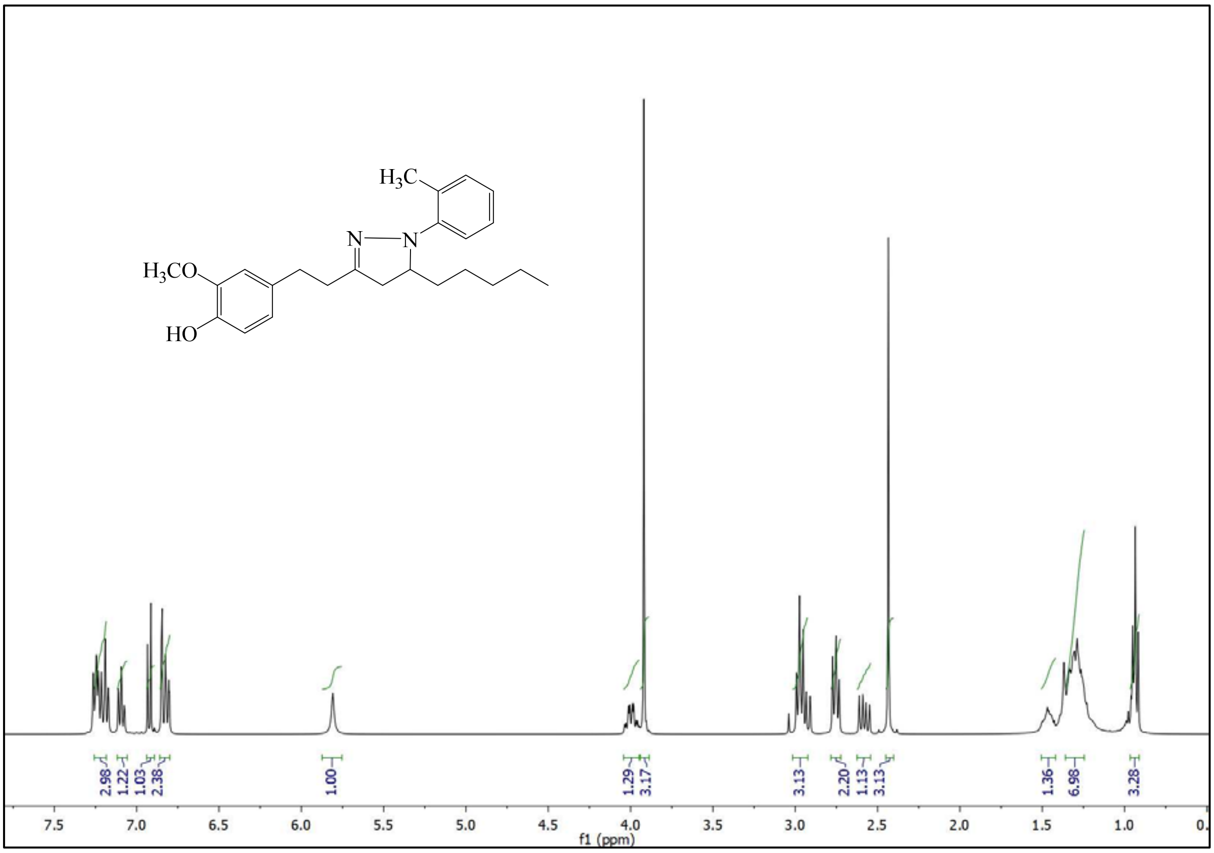

Figure S29. ^{1}H NMR spectrum of **5b**.

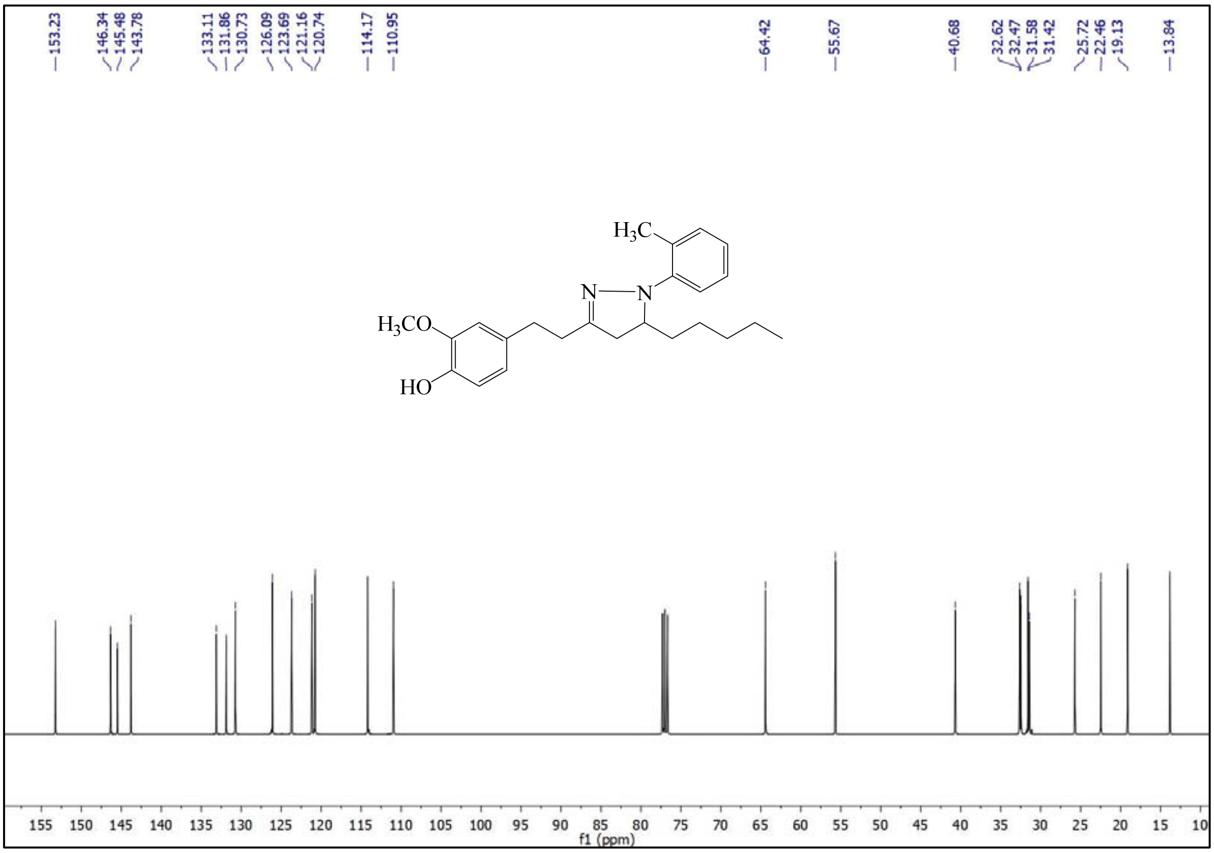

Figure S30. ^{13}C NMR spectrum of **5b**.

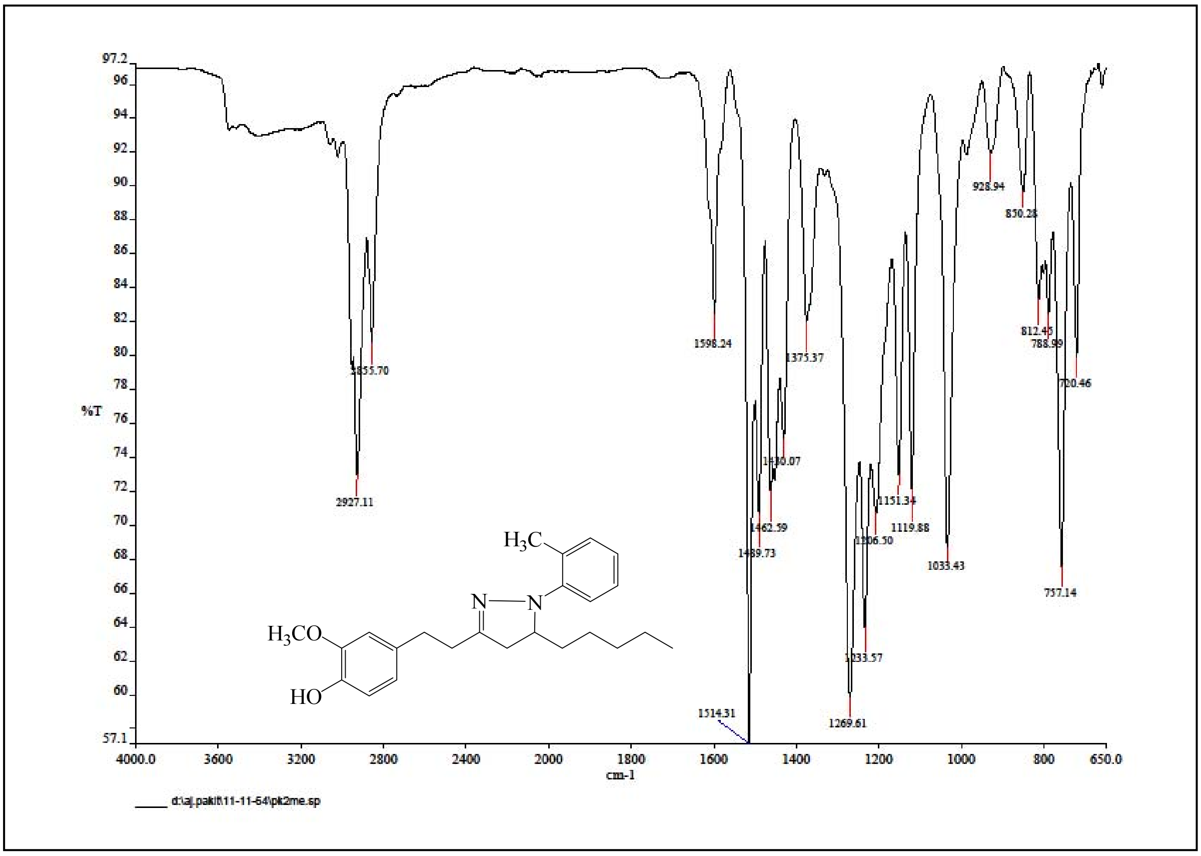

Figure S31. IR spectrum of **5b.**

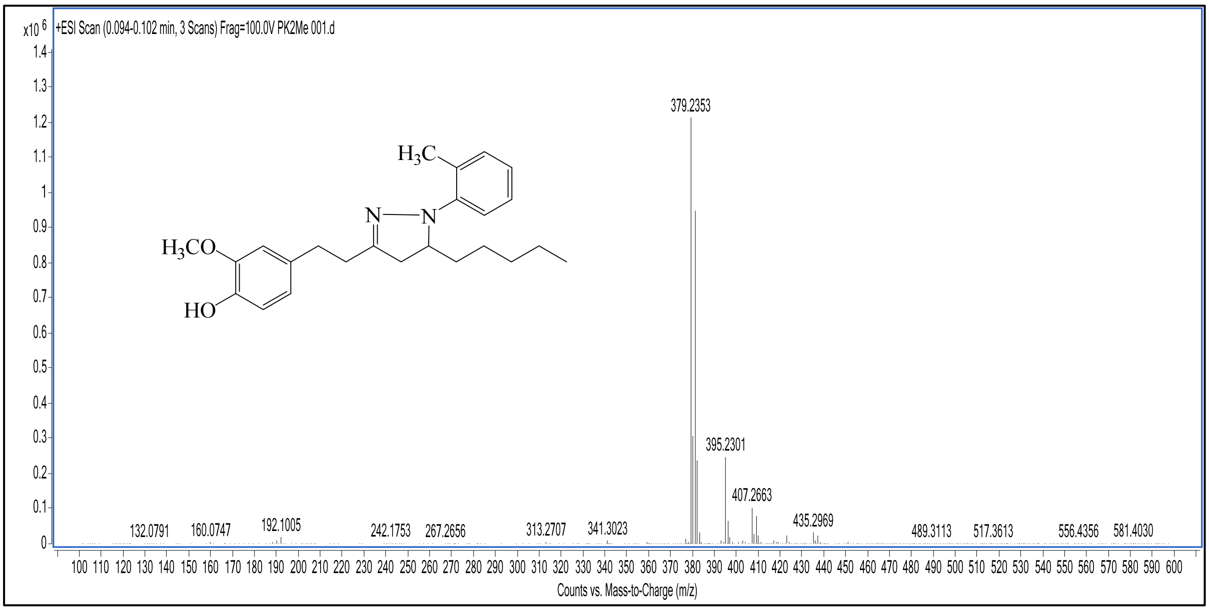

Figure S32. Mass spectrum of **5b**.

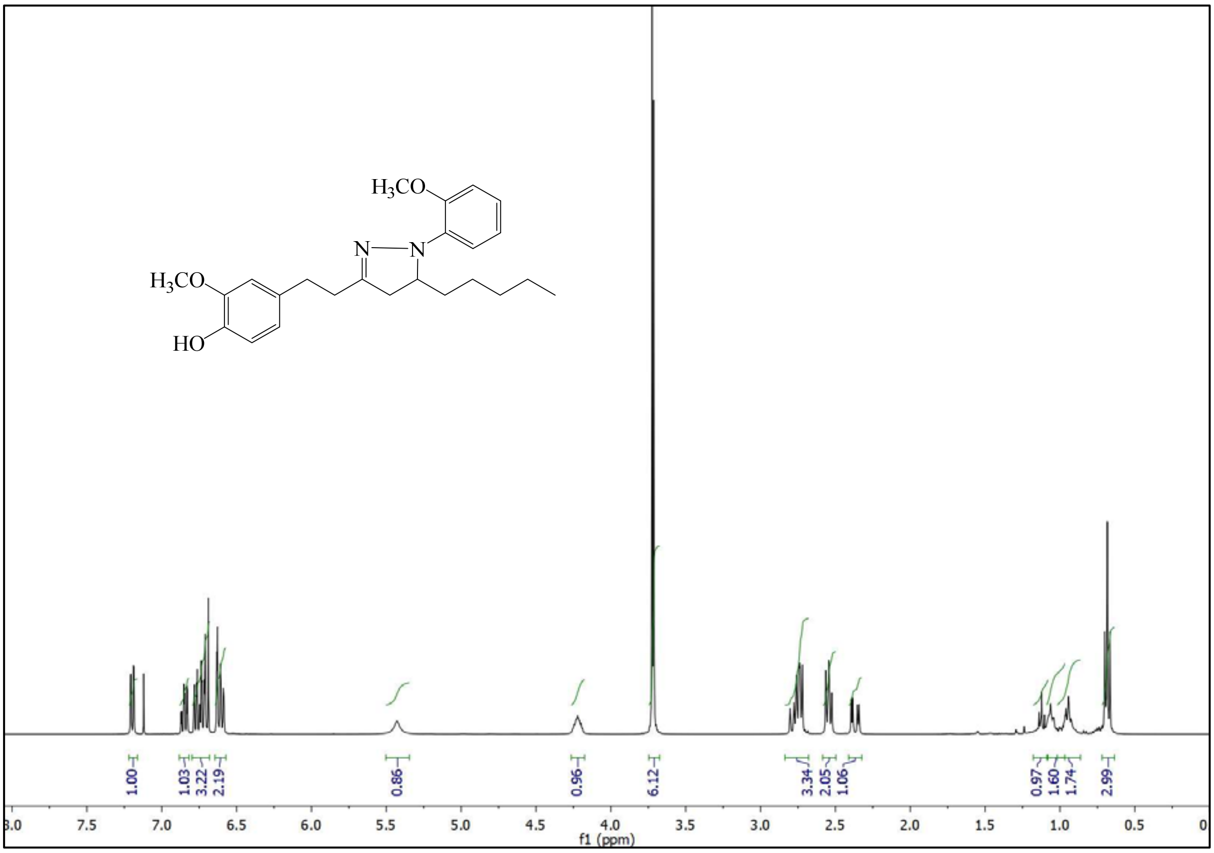

Figure S33. ^{1}H NMR spectrum of **5c**.

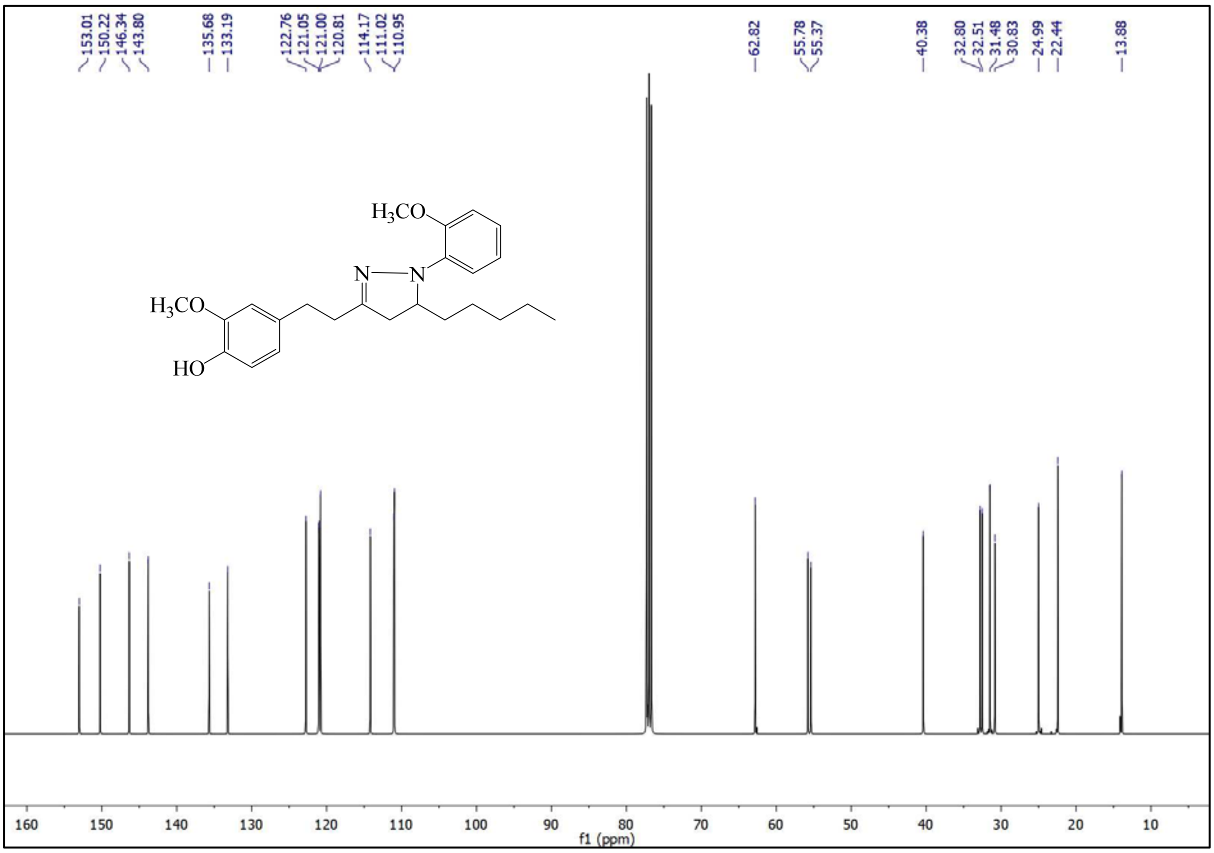

Figure S34. ^{13}C NMR spectrum of **5c**.

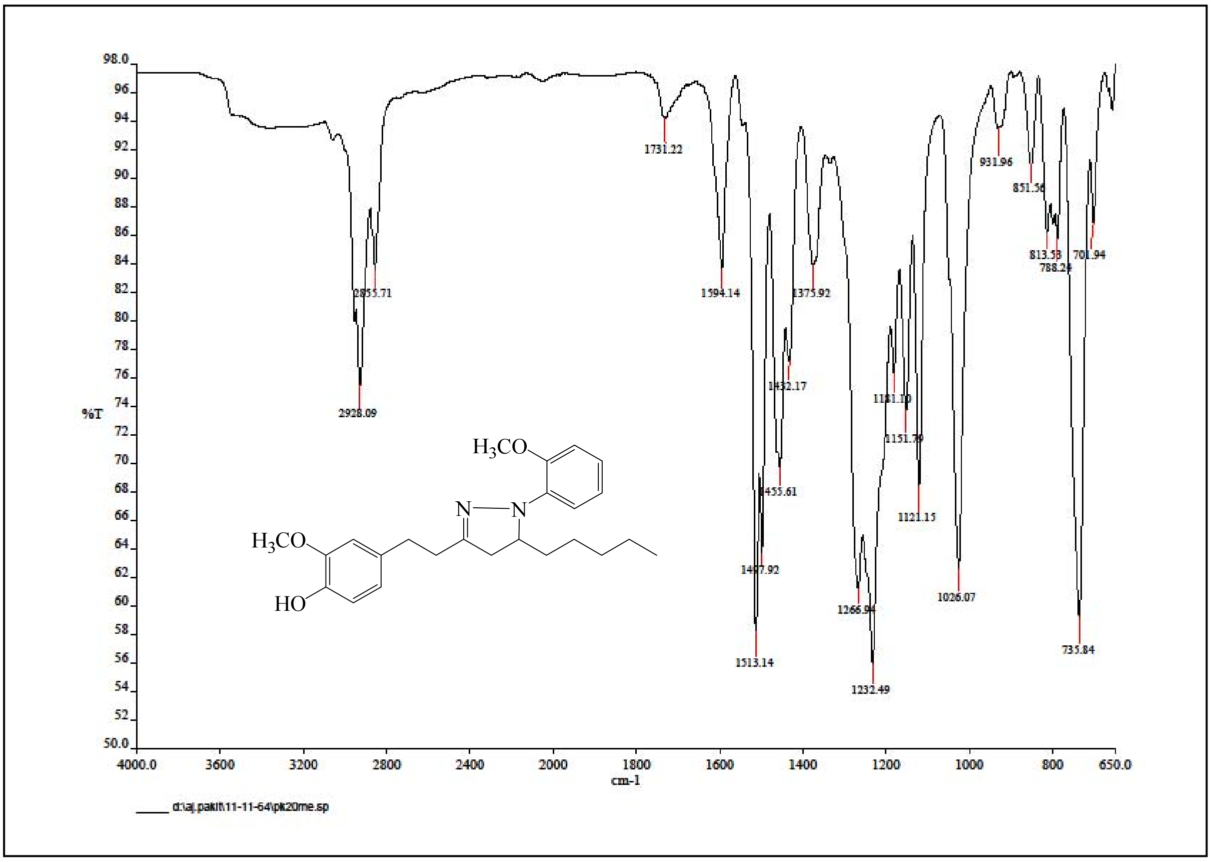

Figure S35. IR spectrum of **5c.**

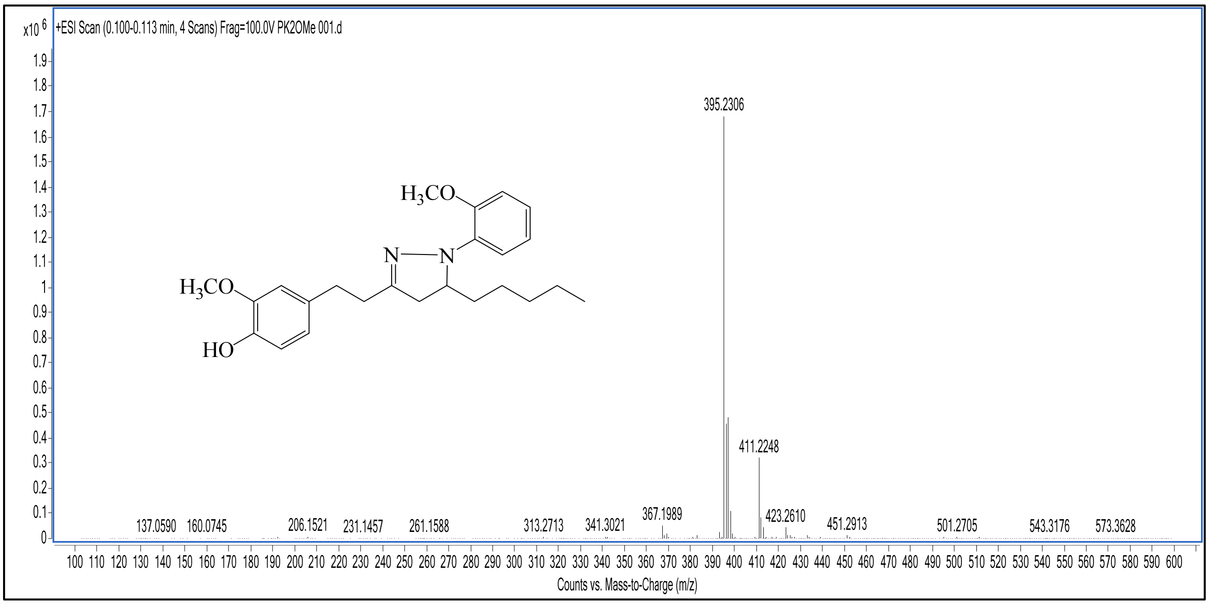

Figure S36. Mass spectrum of **5c**.

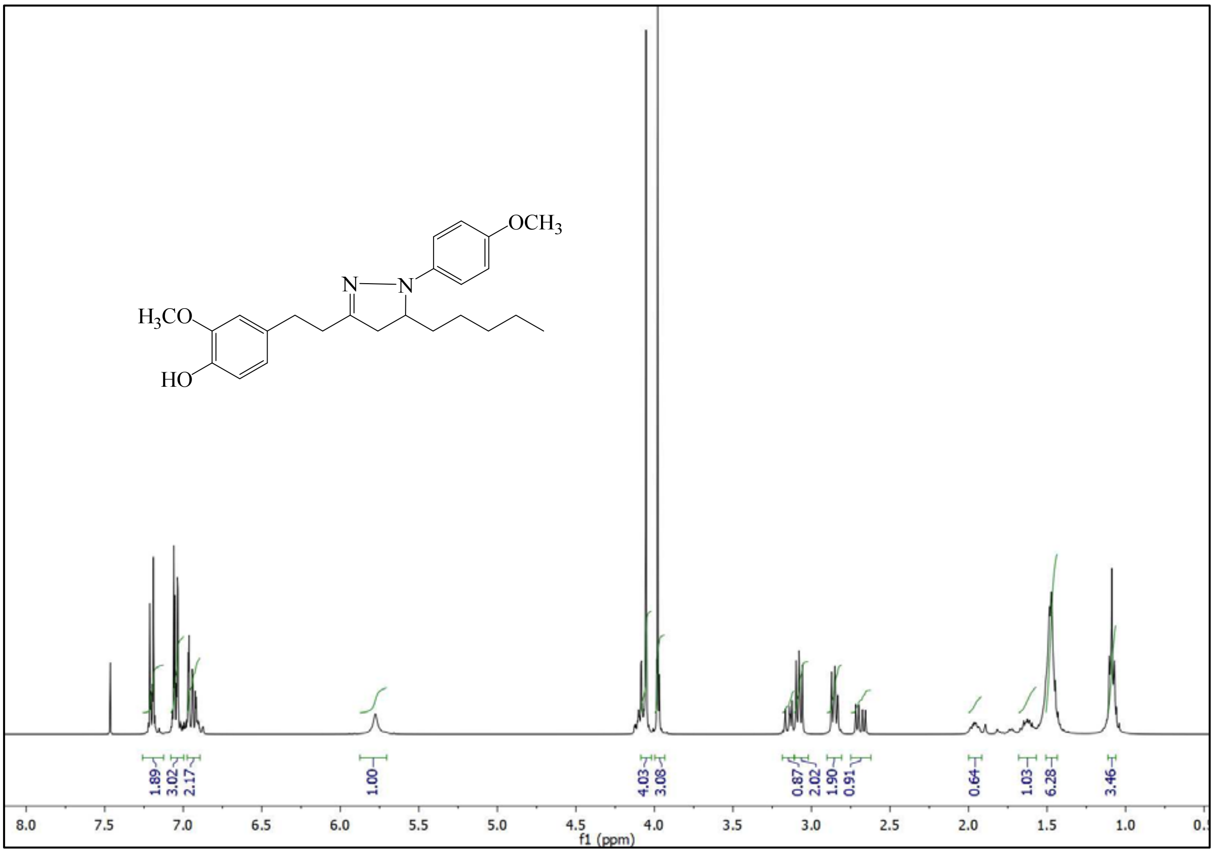

Figure S37. ^{1}H NMR spectrum of **5d**.

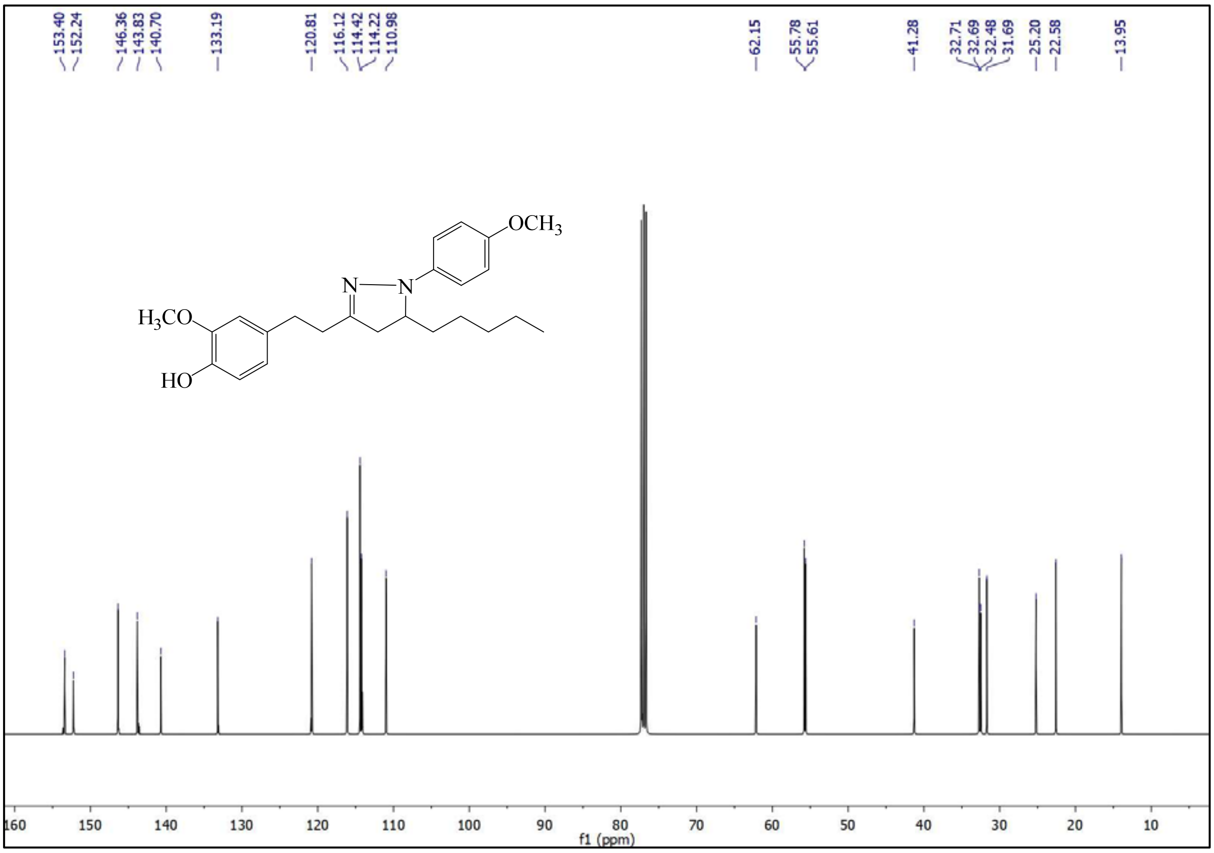

Figure S38. ^{13}C NMR spectrum of **5d**.

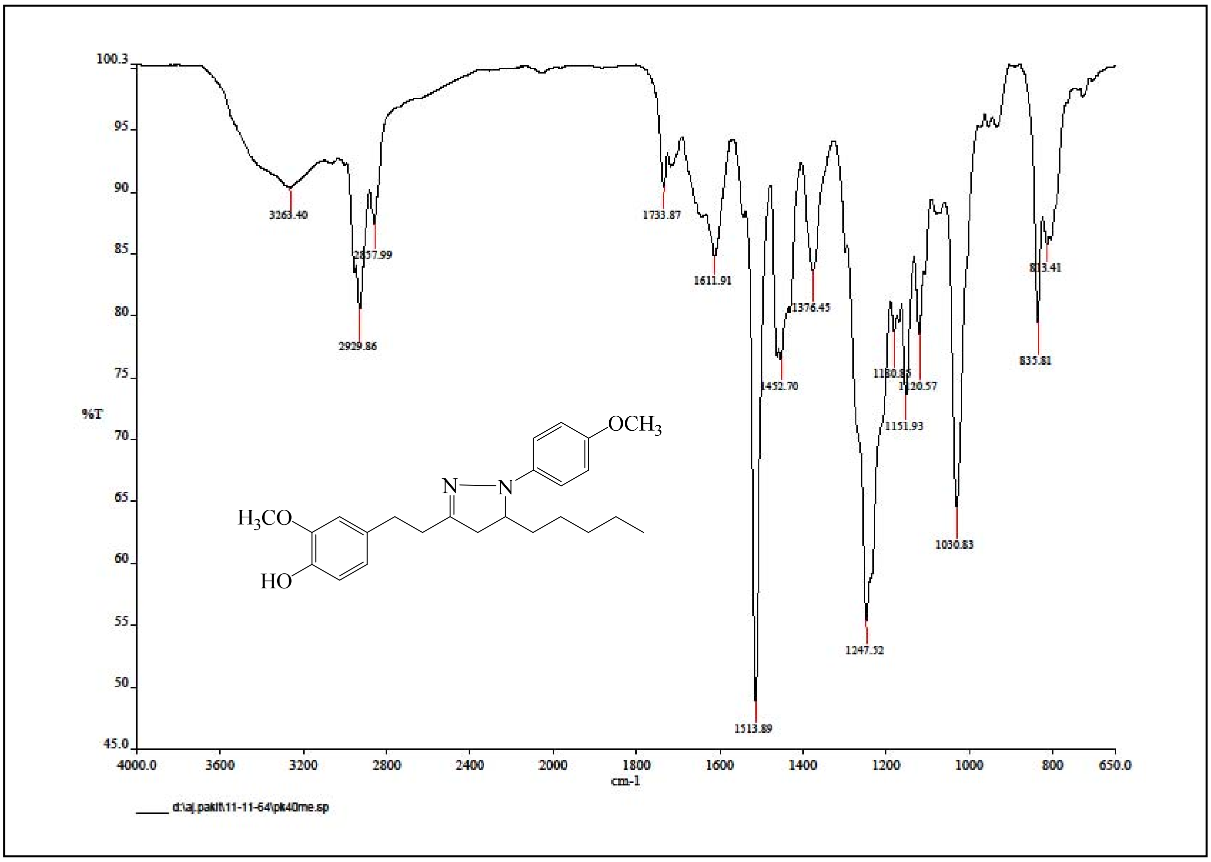

Figure S39. IR spectrum of **5d.**

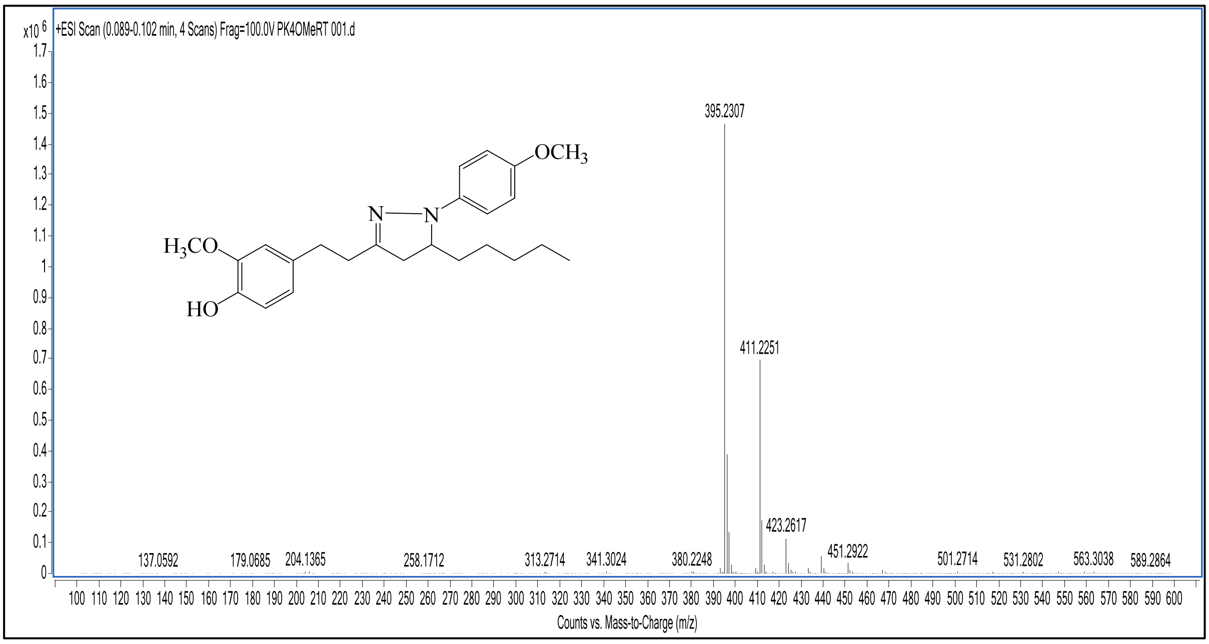

Figure S40. Mass spectrum of **5d**.

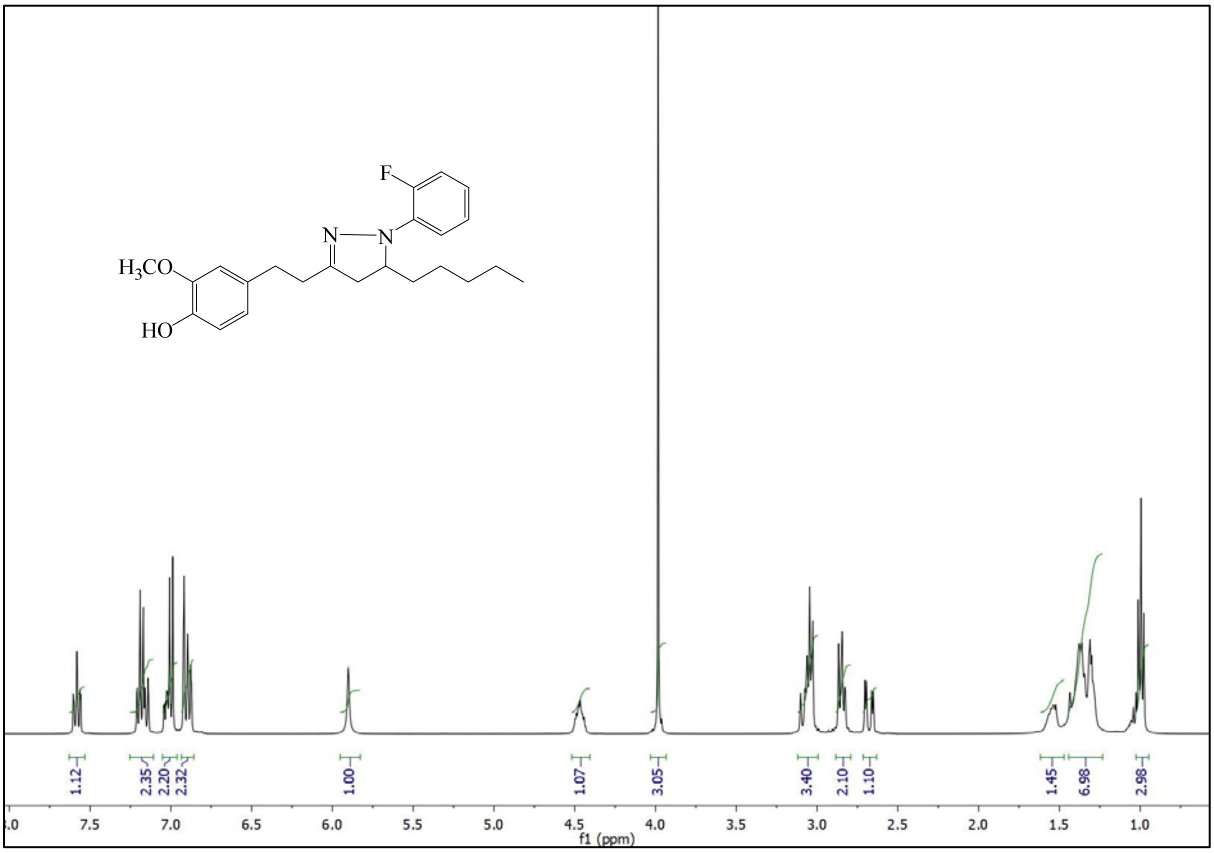

Figure S41. ^{1}H NMR spectrum of **5e**.

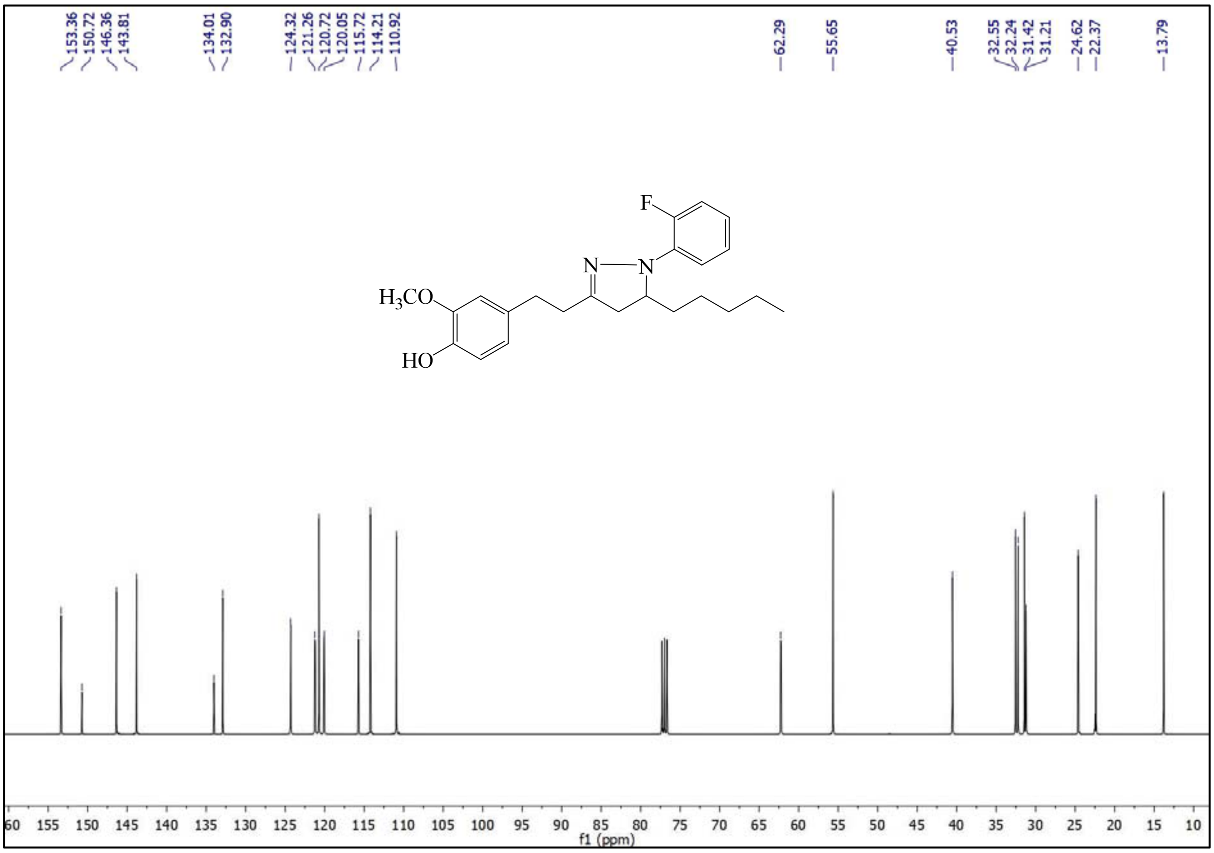

Figure S42. ^{13}C NMR spectrum of **5e**.

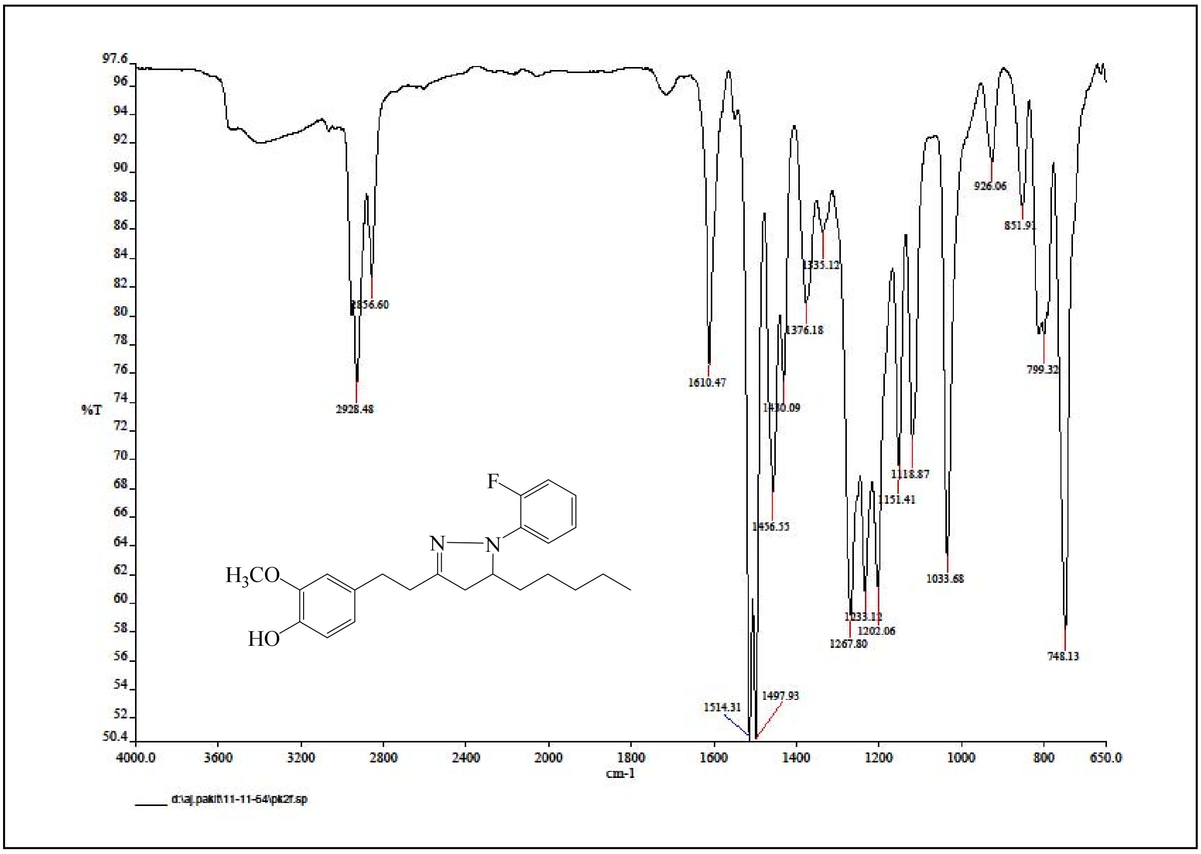

Figure S43. IR spectrum of **5e.**

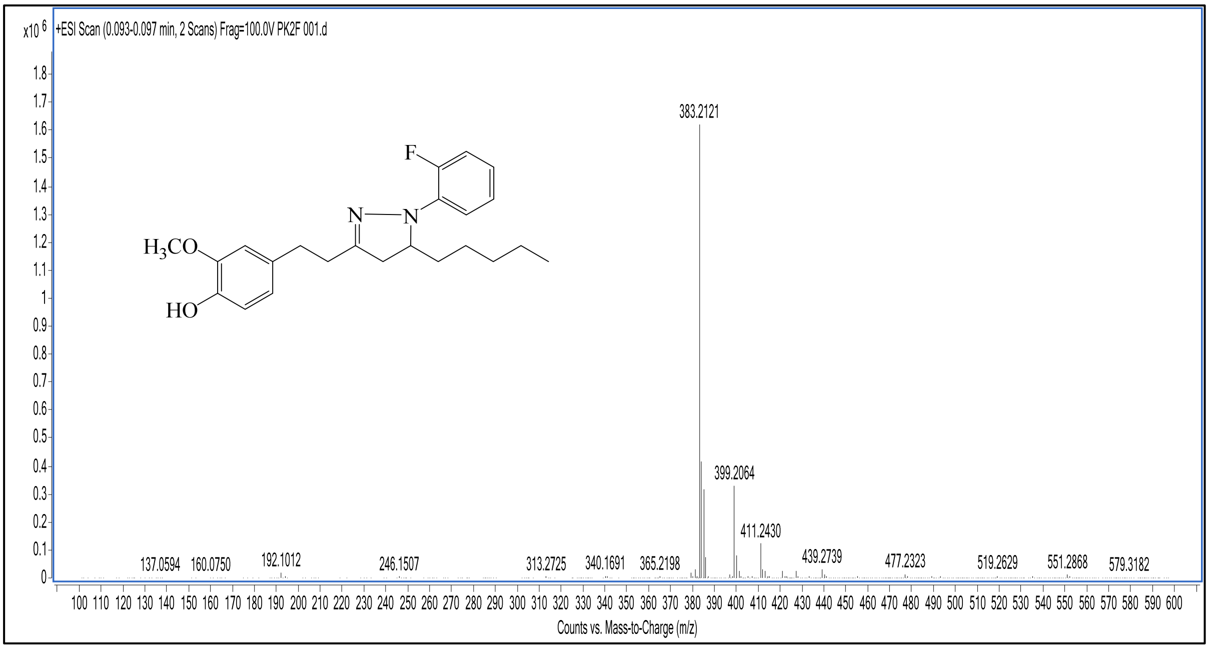

Figure S44. Mass spectrum of **5e**.

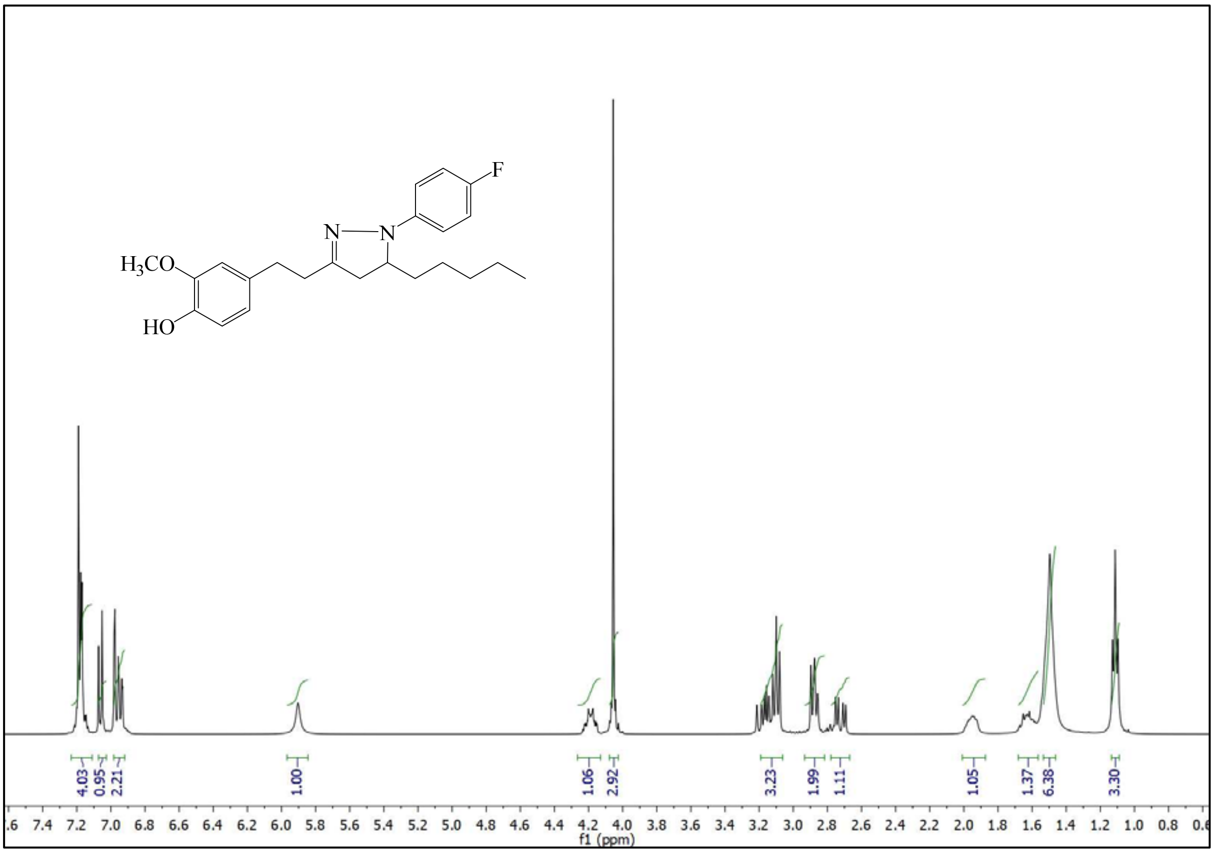

Figure S45. ^{1}H NMR spectrum of **5f**.

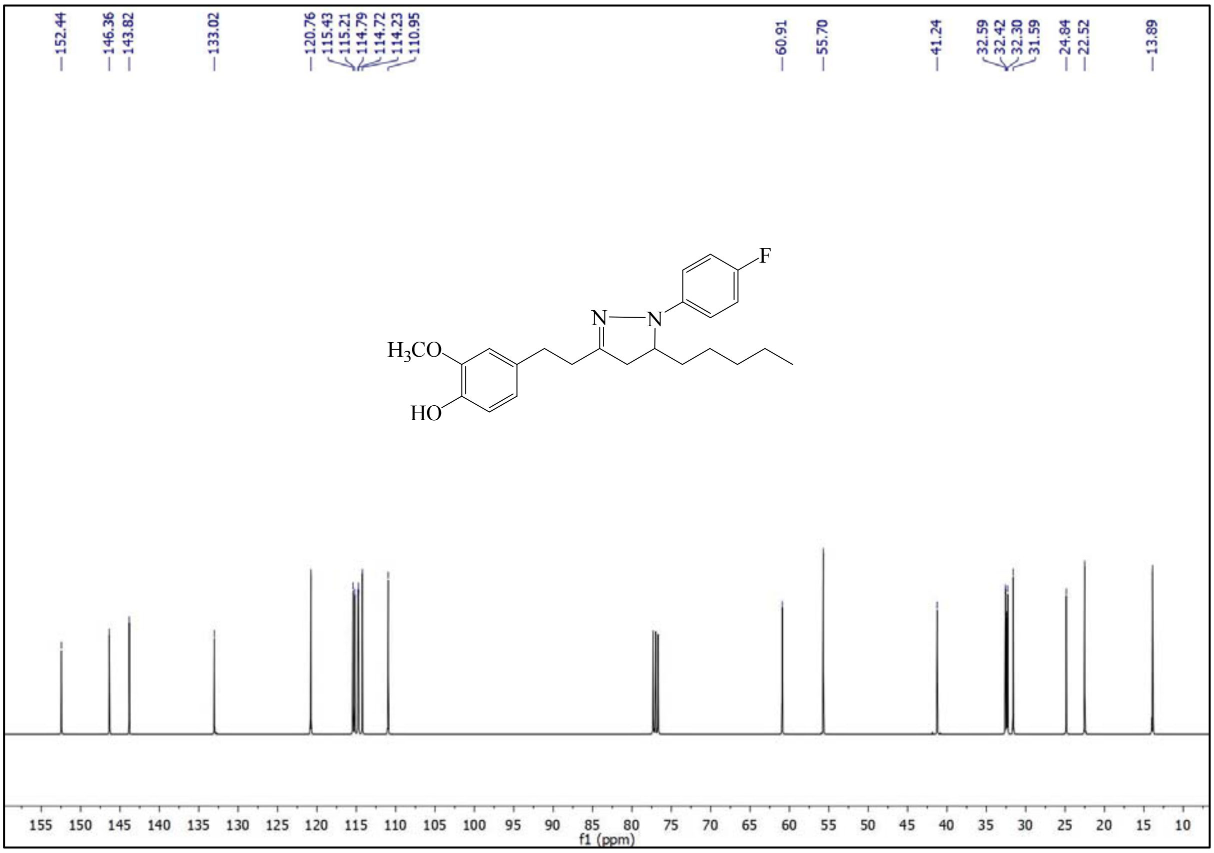

Figure S46. ^{13}C NMR spectrum of **5f**.

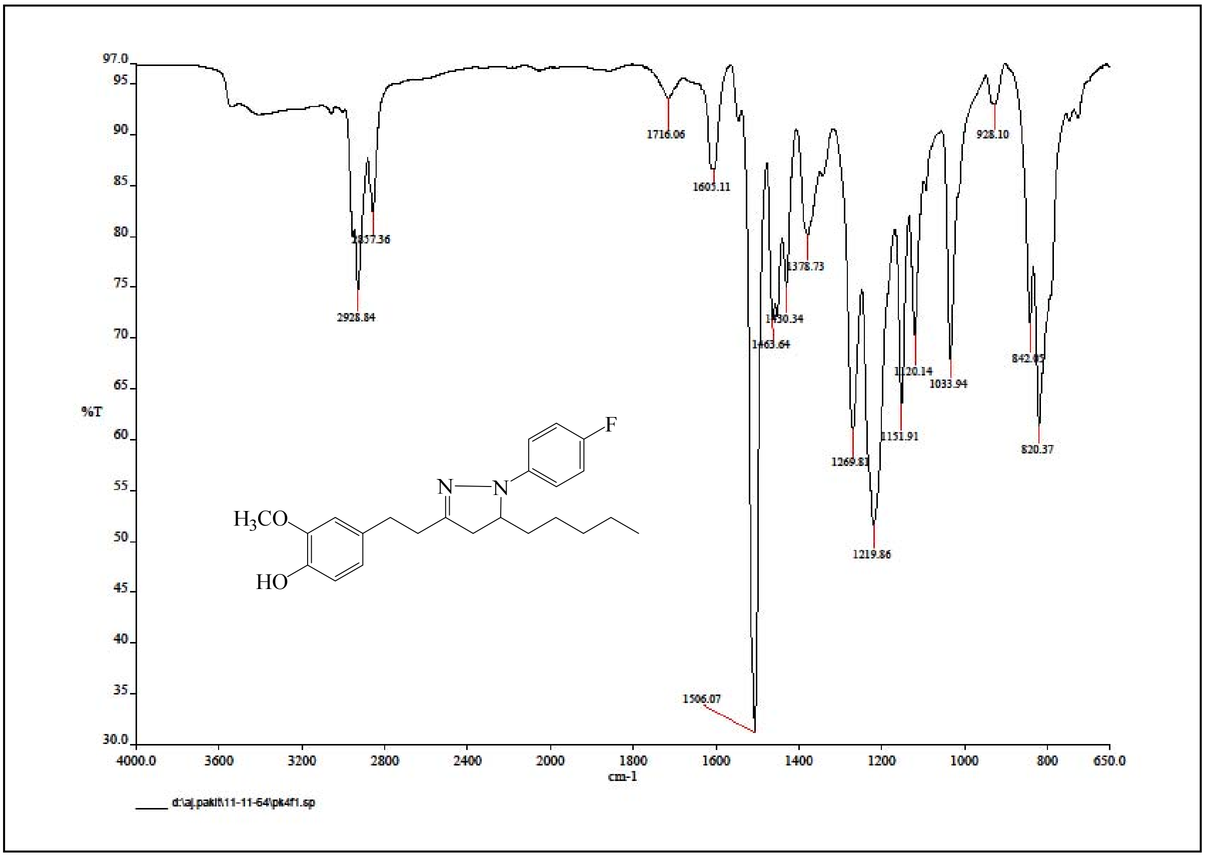

Figure S47. IR spectrum of **5f.**

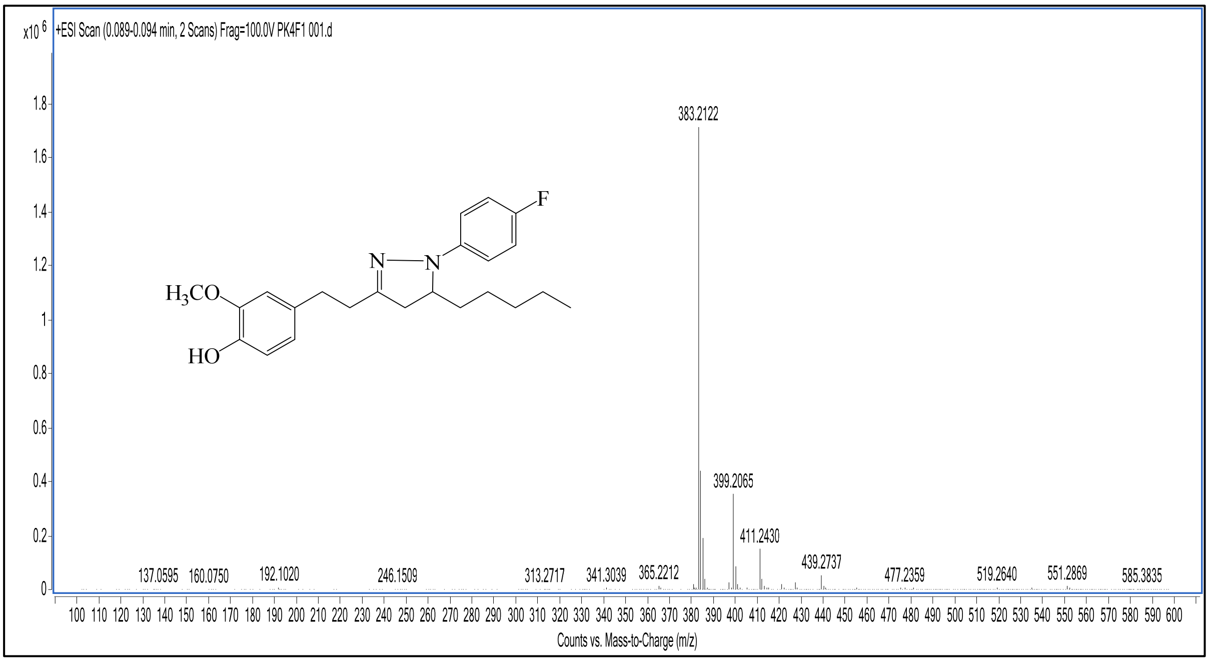

Figure S48. Mass spectrum of **5f**.

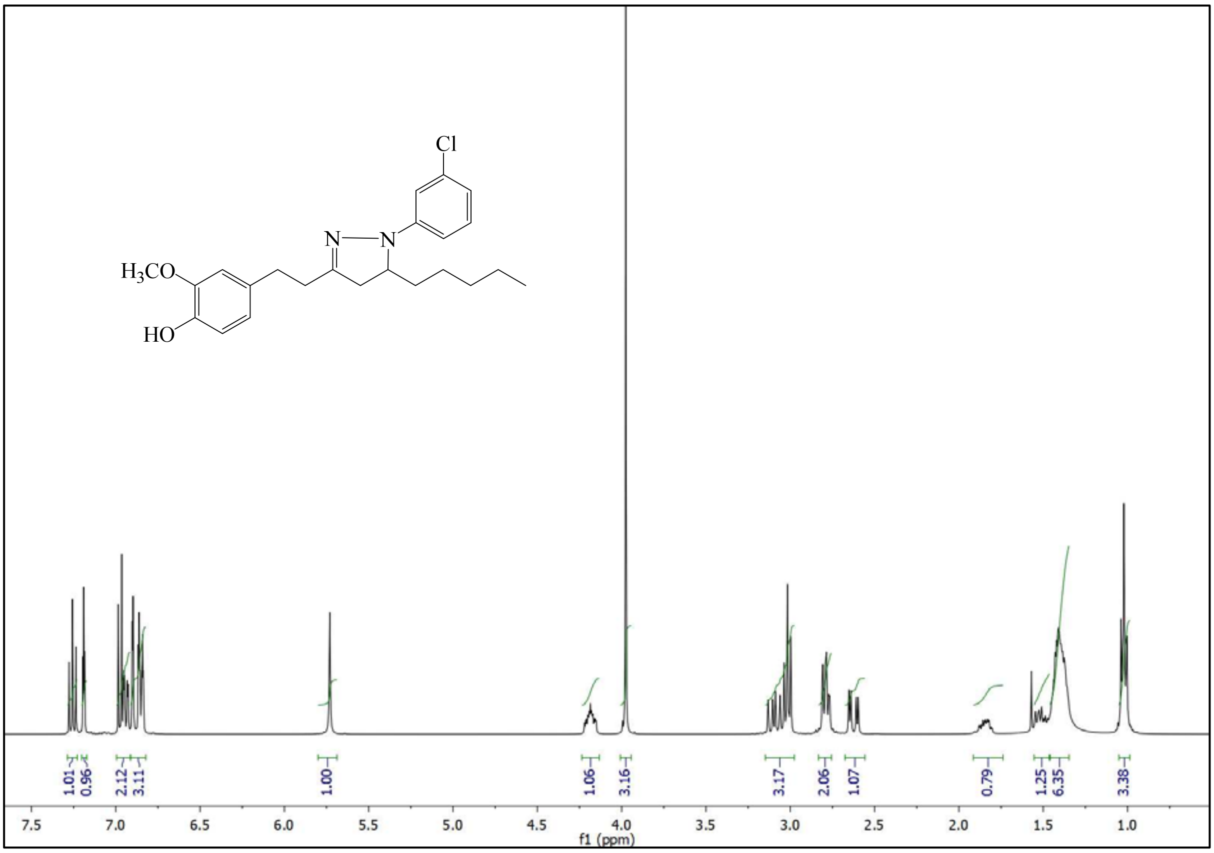

Figure S49. ^{1}H NMR spectrum of **5g**.

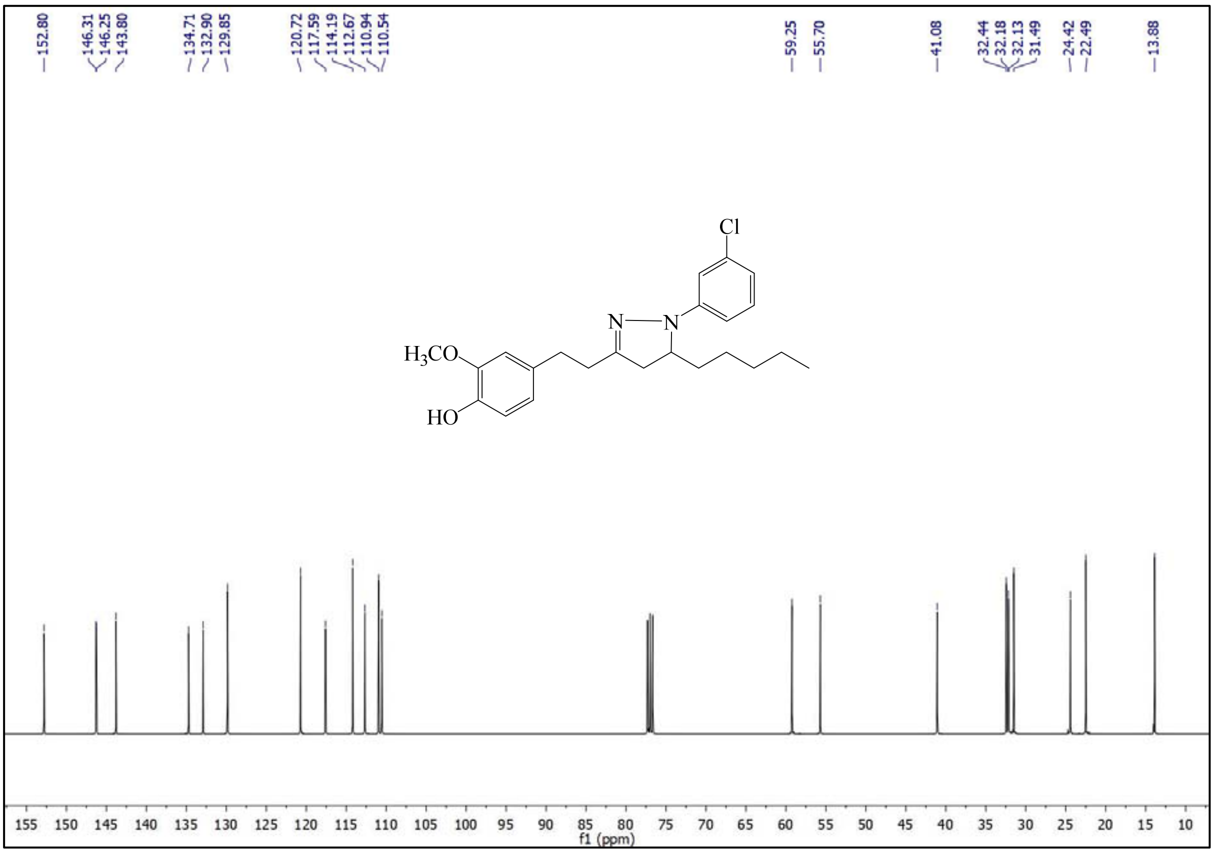

Figure S50. ^{13}C NMR spectrum of **5g**.

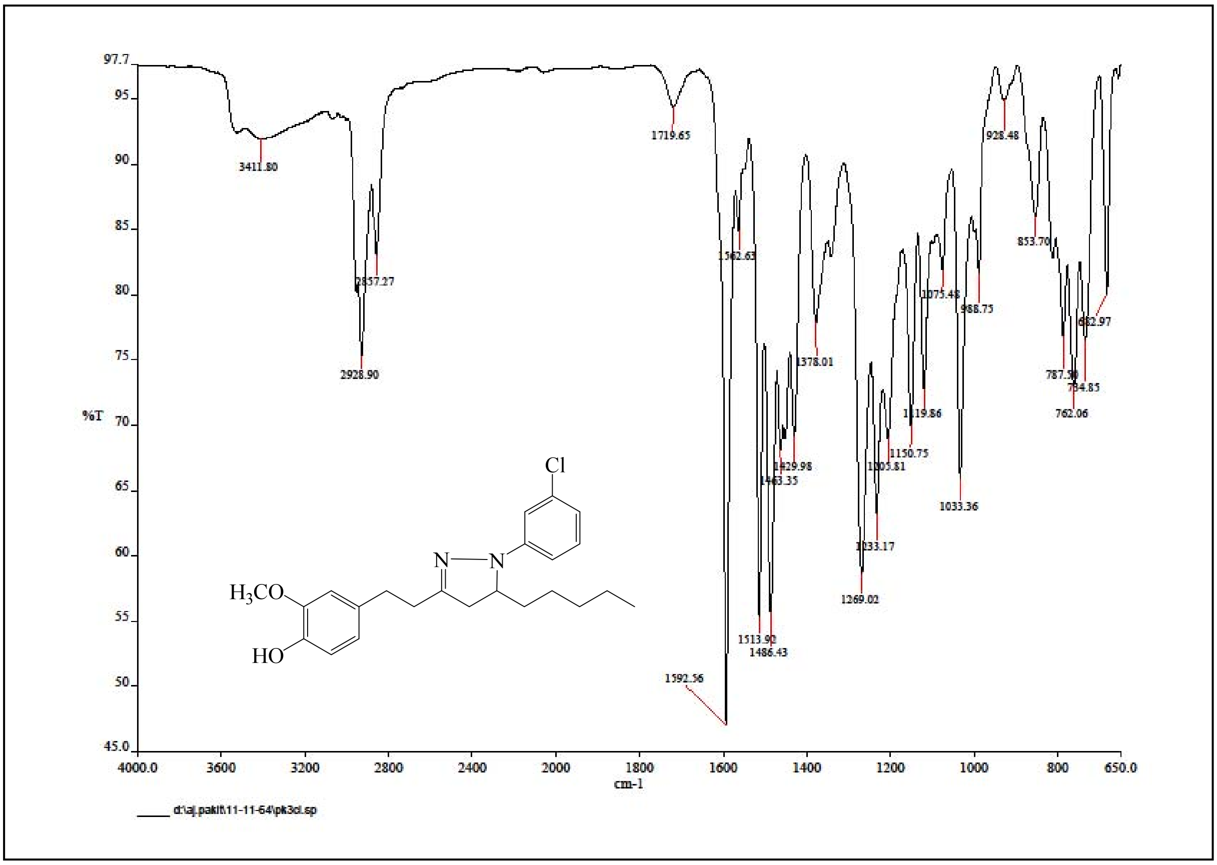

Figure S51. IR spectrum of **5g.**

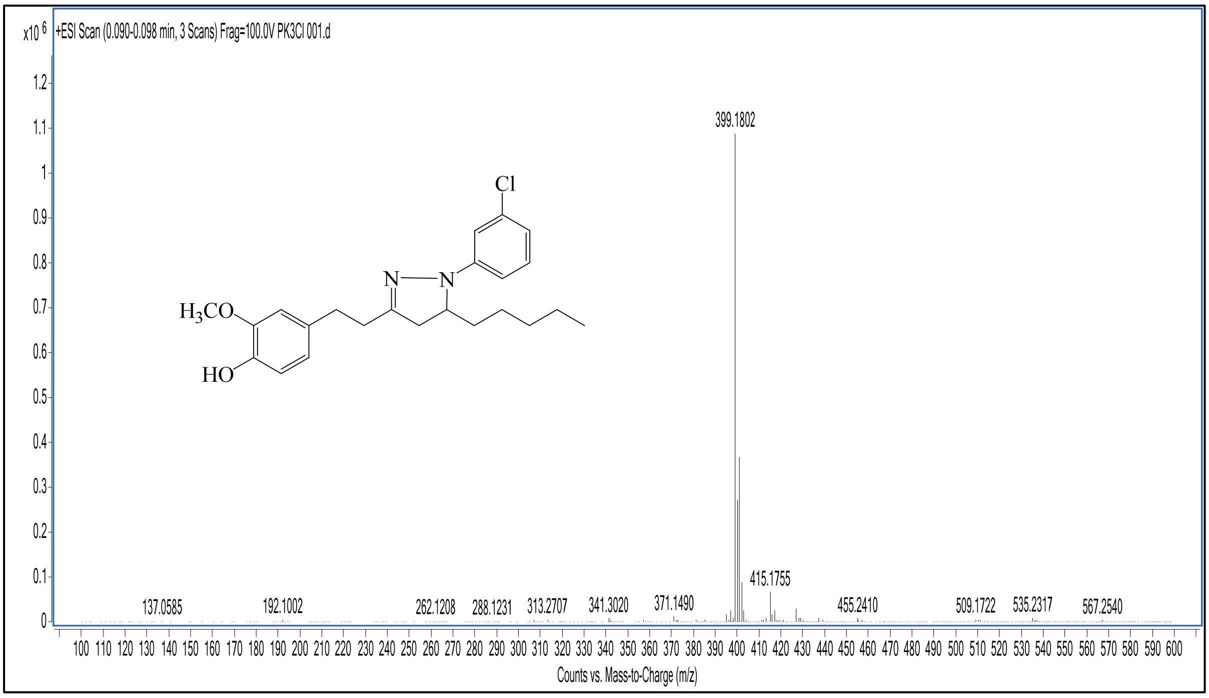

Figure S52. Mass spectrum of **5g**.

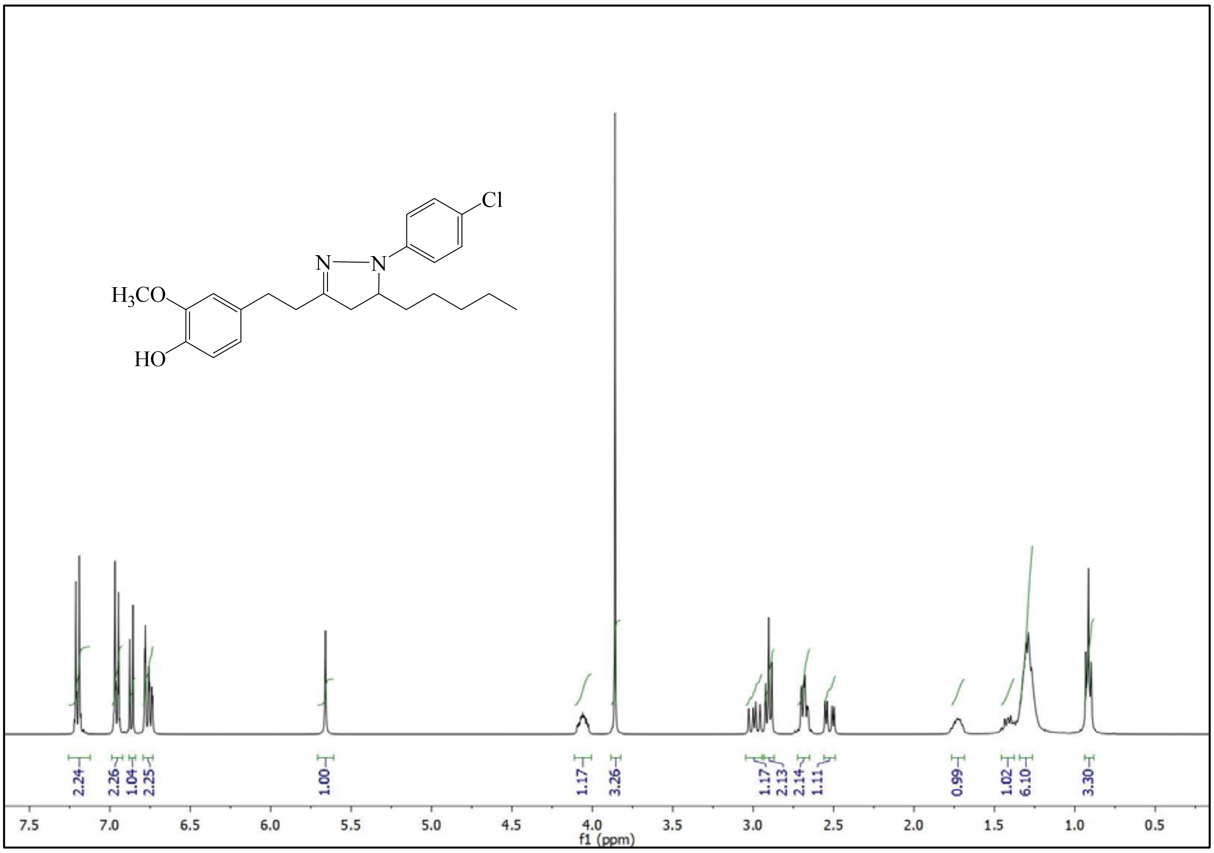

Figure S53. ^{1}H NMR spectrum of **5h**.

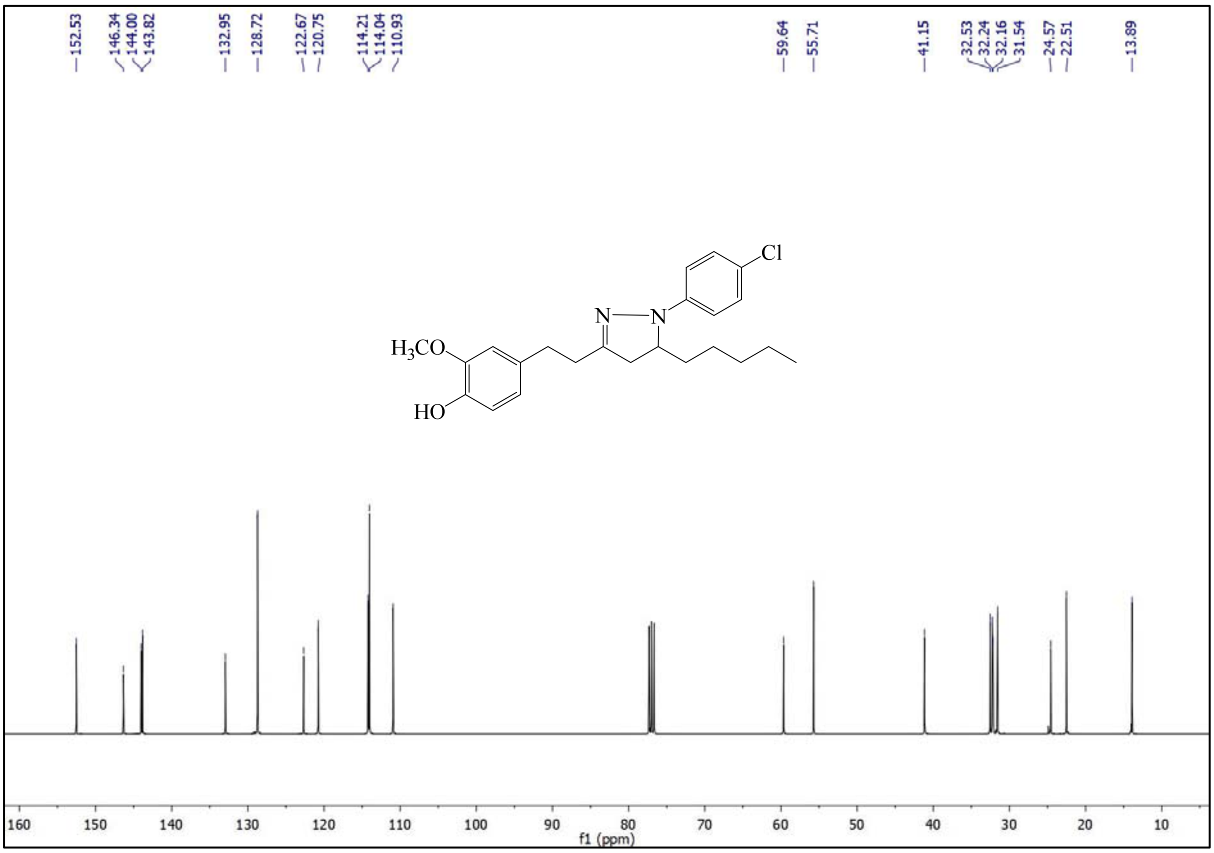

Figure S54. ^{13}C NMR spectrum of **5h**.

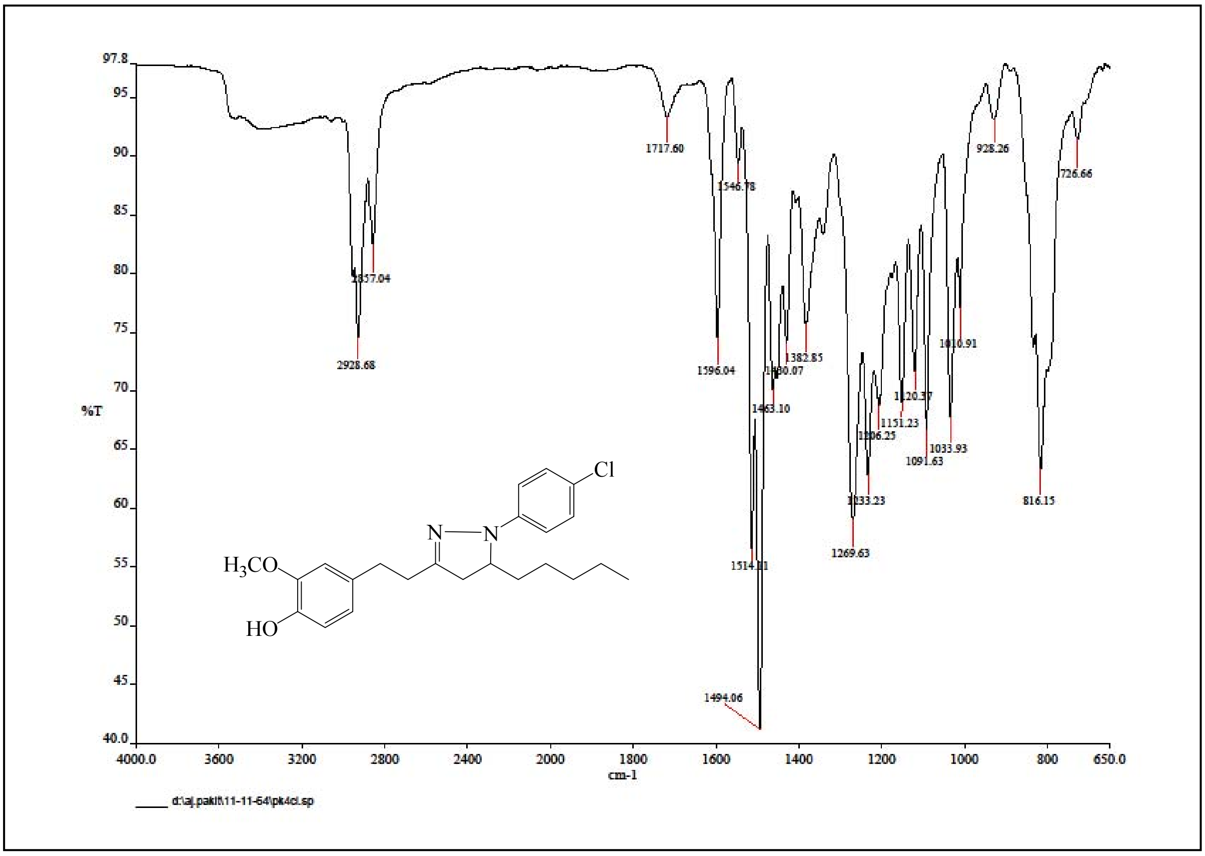

Figure S55. IR spectrum of **5h.**

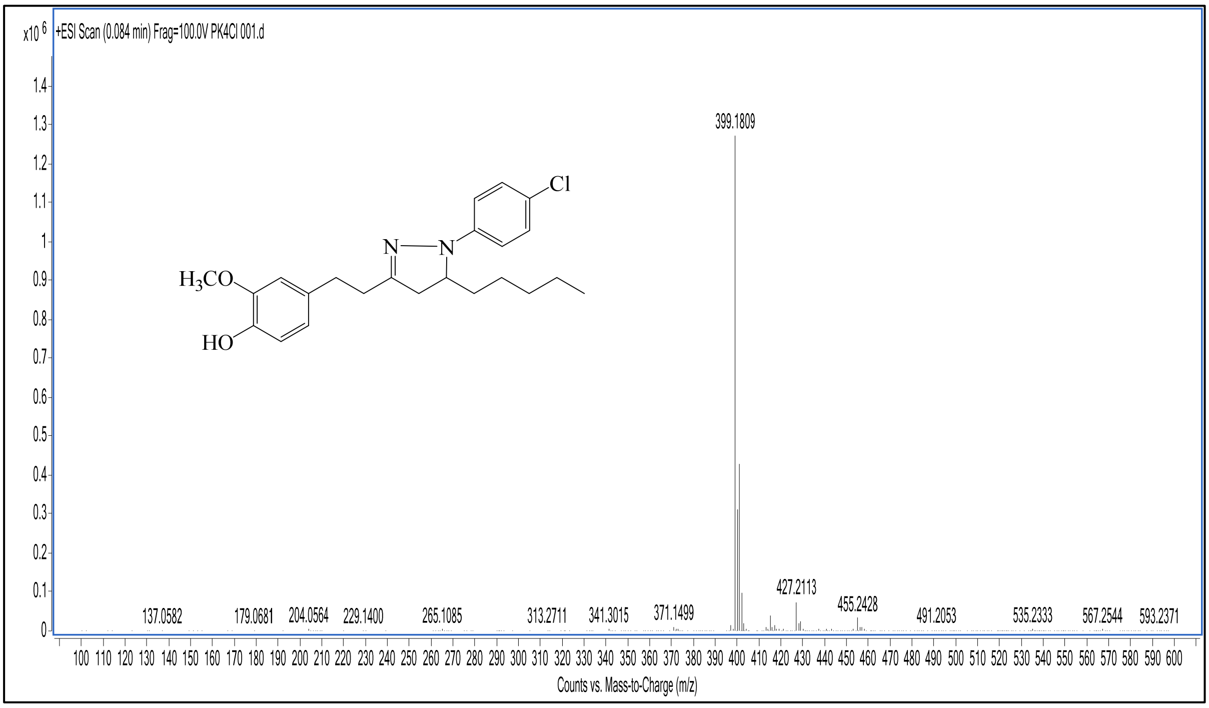

Figure S56. Mass spectrum of **5h**.

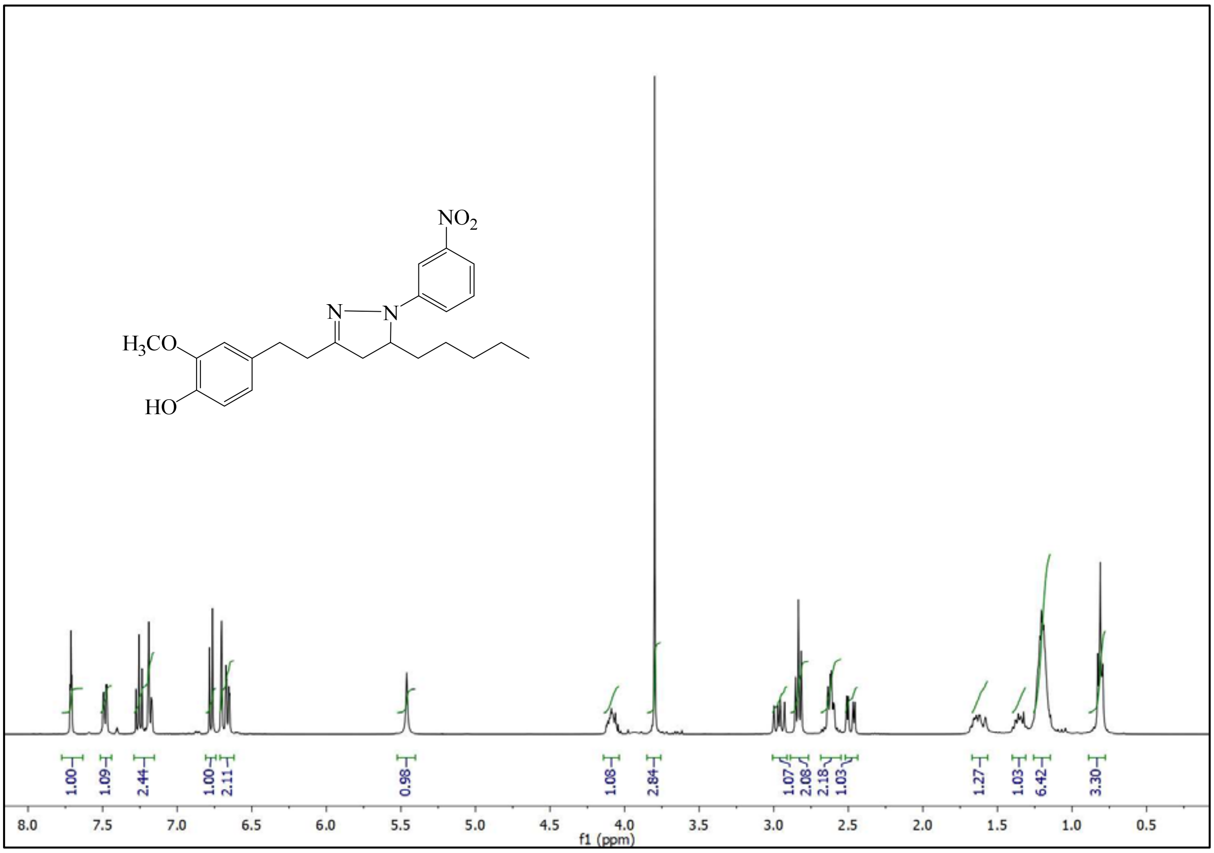

Figure S57. ^{1}H NMR spectrum of **5i**.

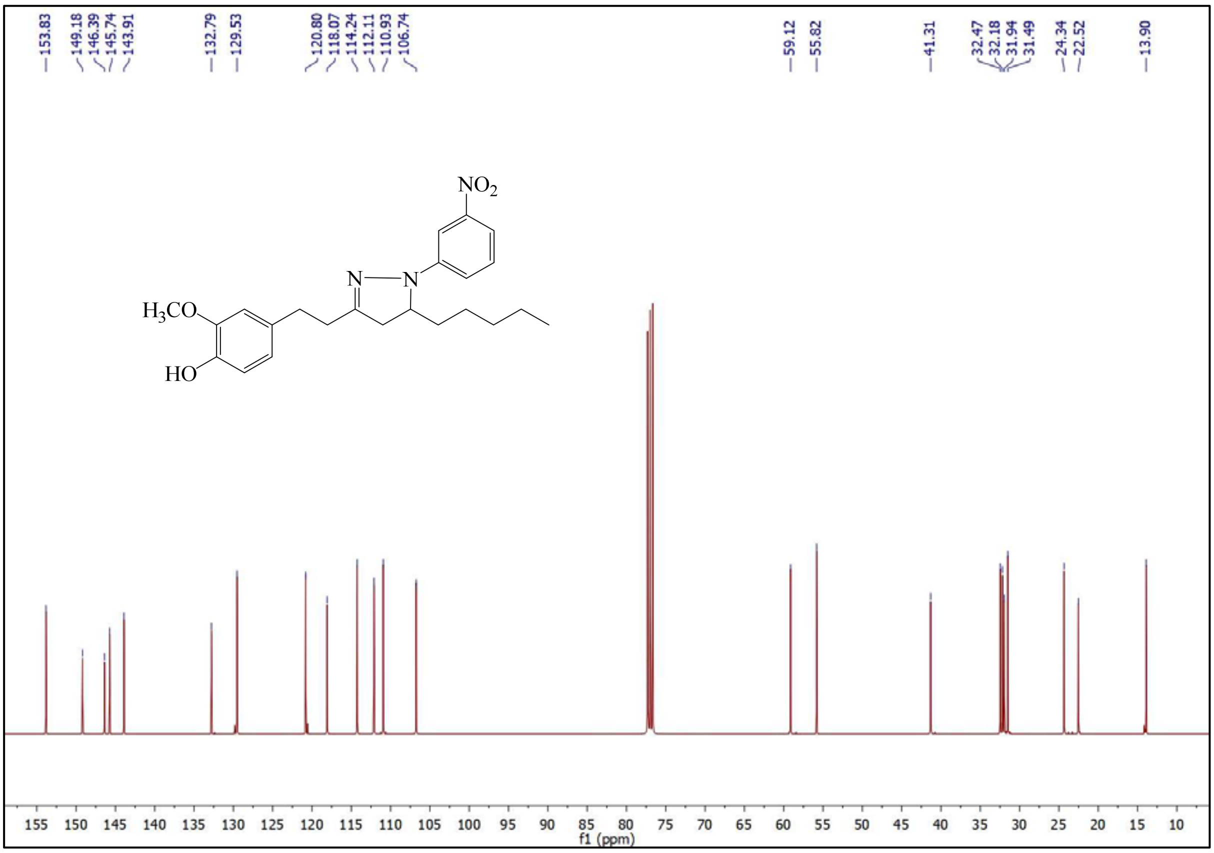

Figure S58. ^{13}C NMR spectrum of **5i**.

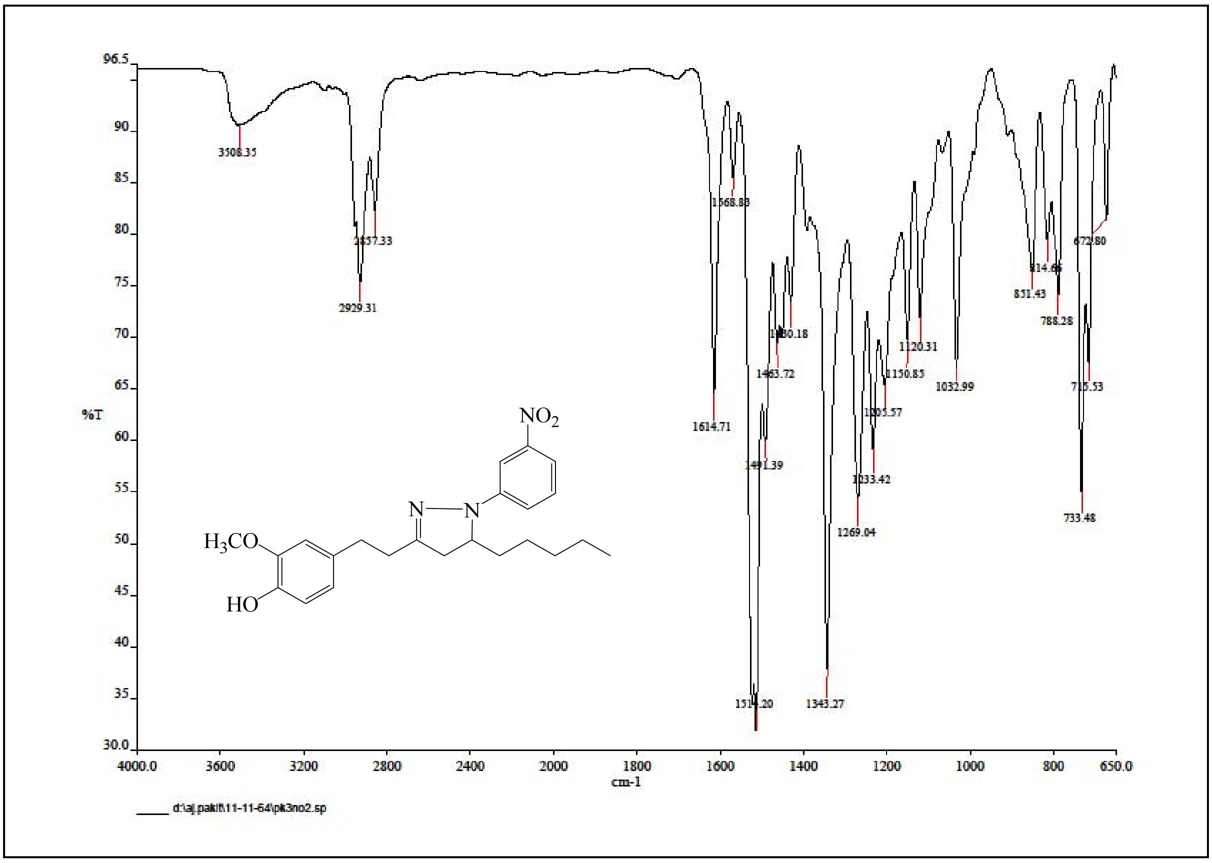

Figure S59. IR spectrum of **5i.**

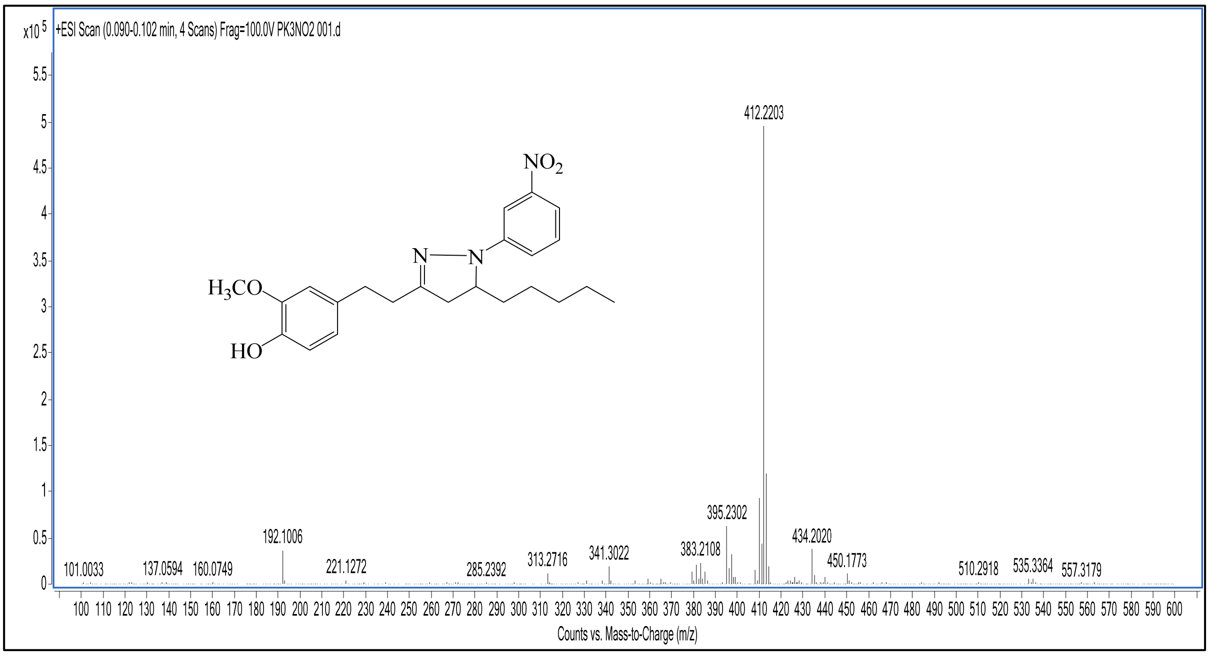

Figure S60. Mass spectrum of **5i**.

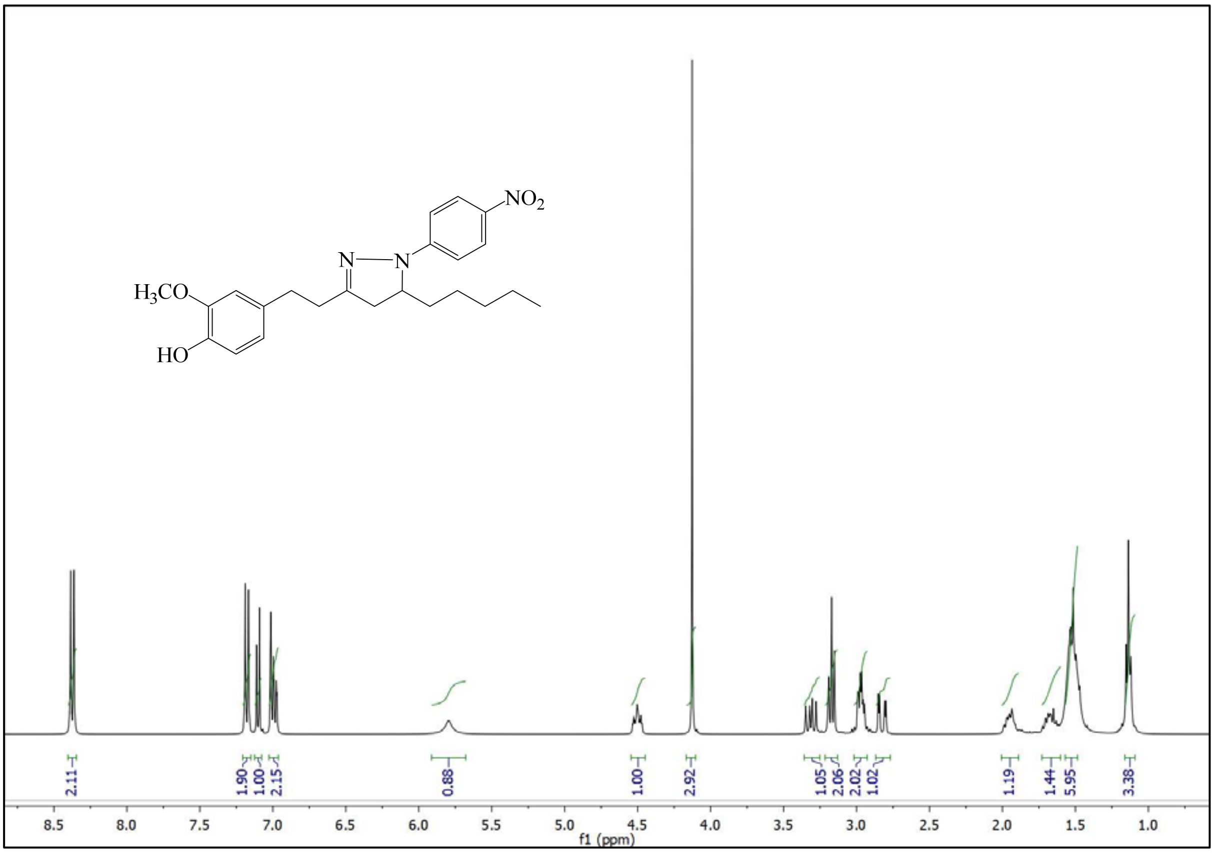

Figure S61. ^{1}H NMR spectrum of **5j**.

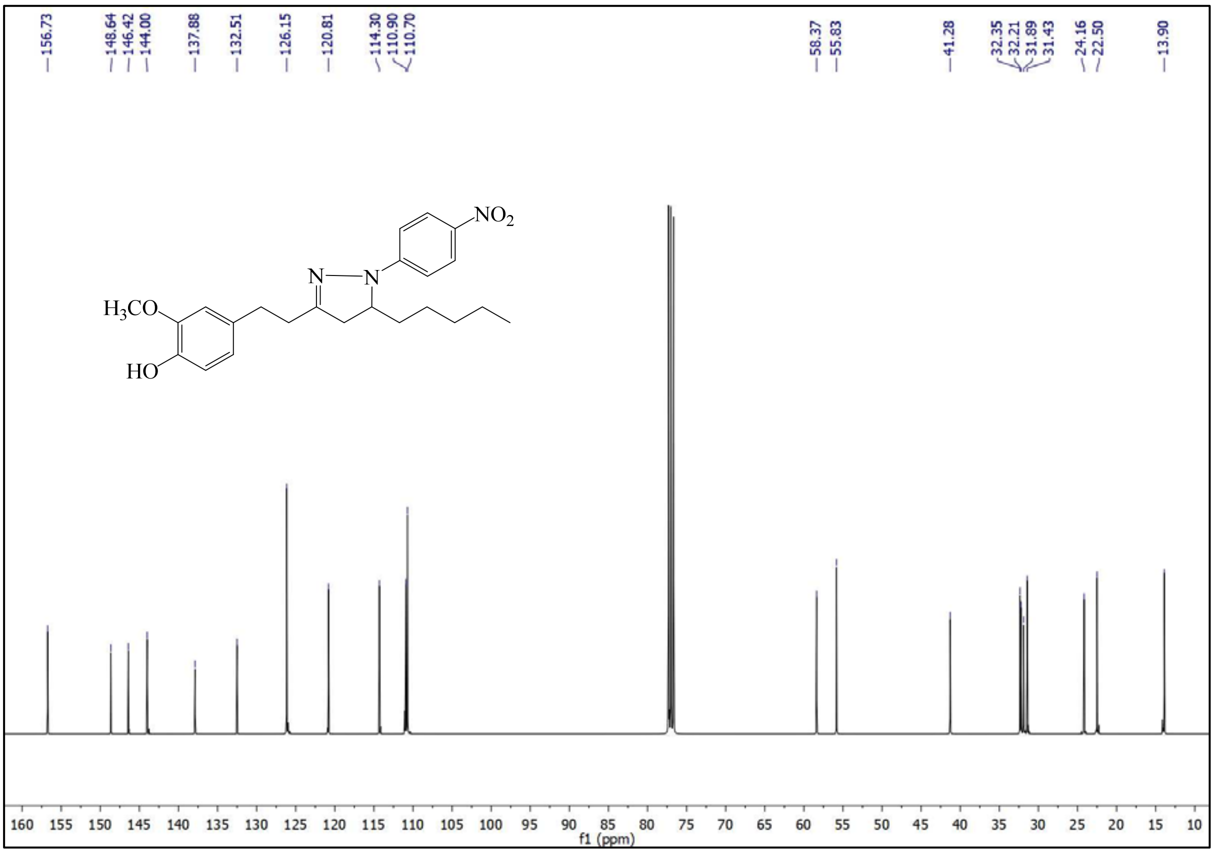

Figure S62. ^{13}C NMR spectrum of **5j**.

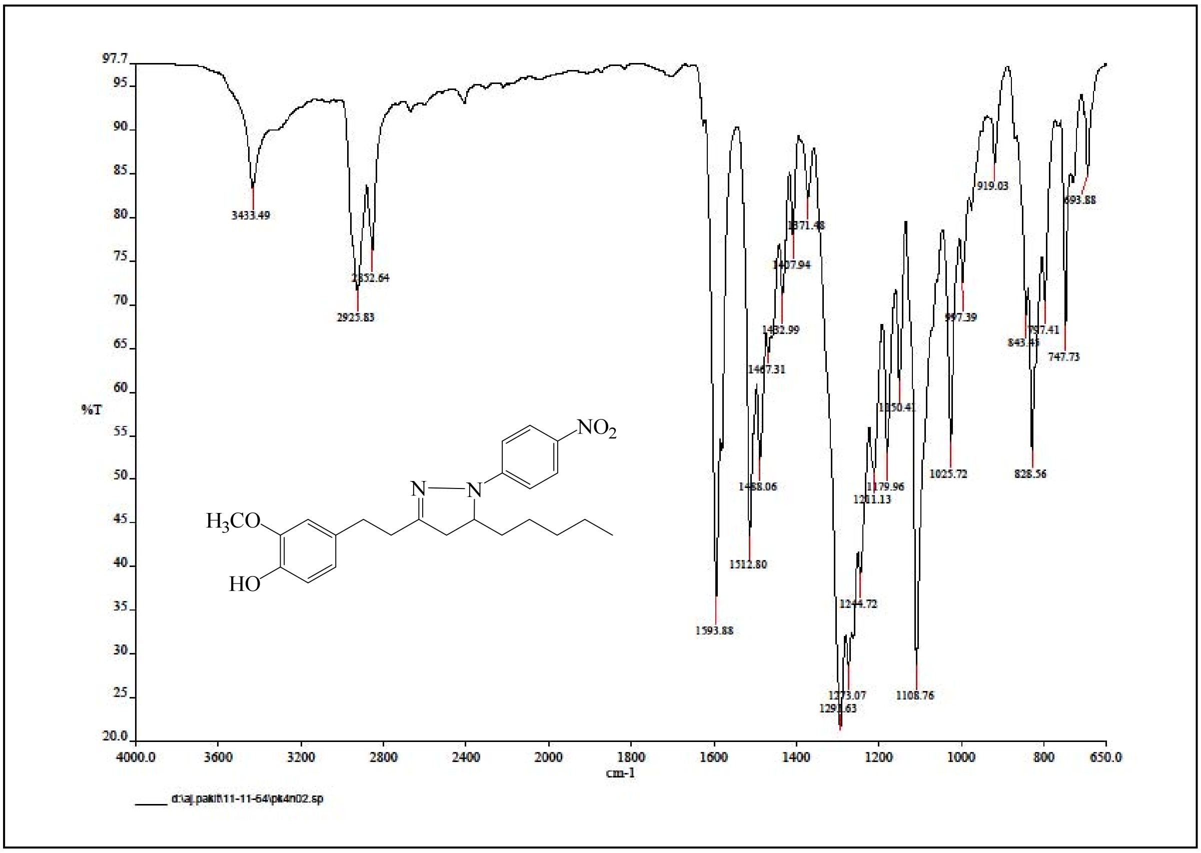

Figure S63. IR spectrum of **5j.**

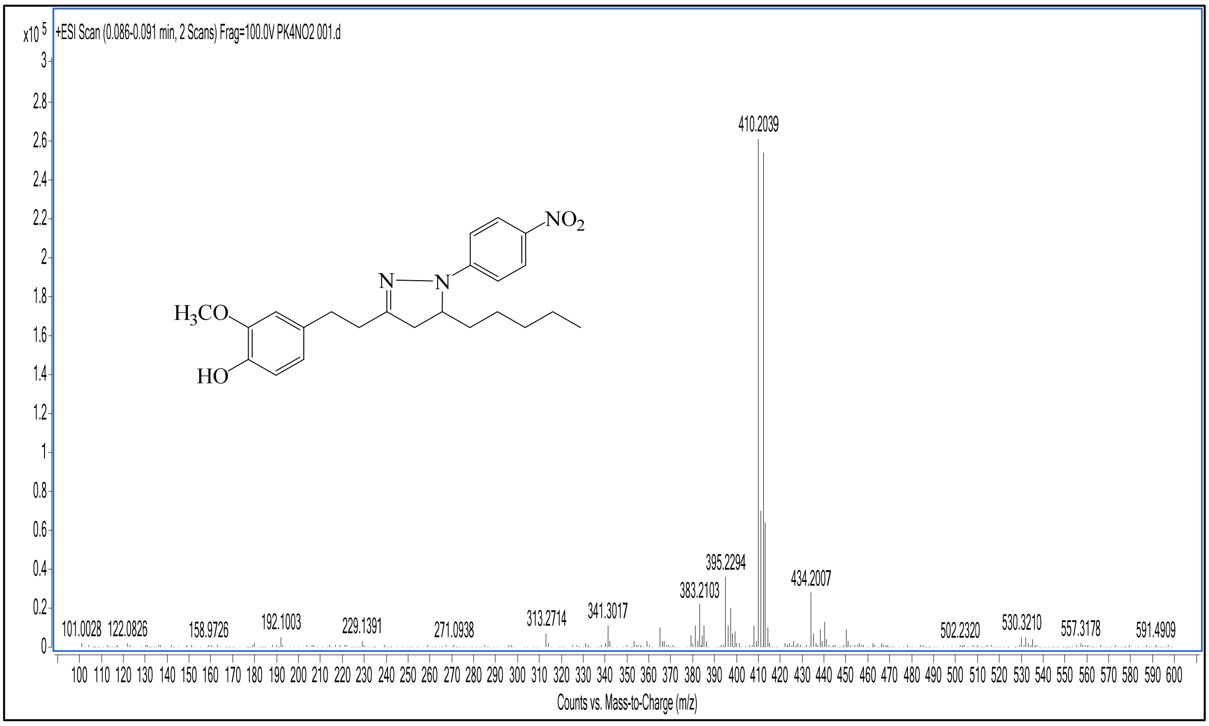

Figure S64. Mass spectrum of **5j**.

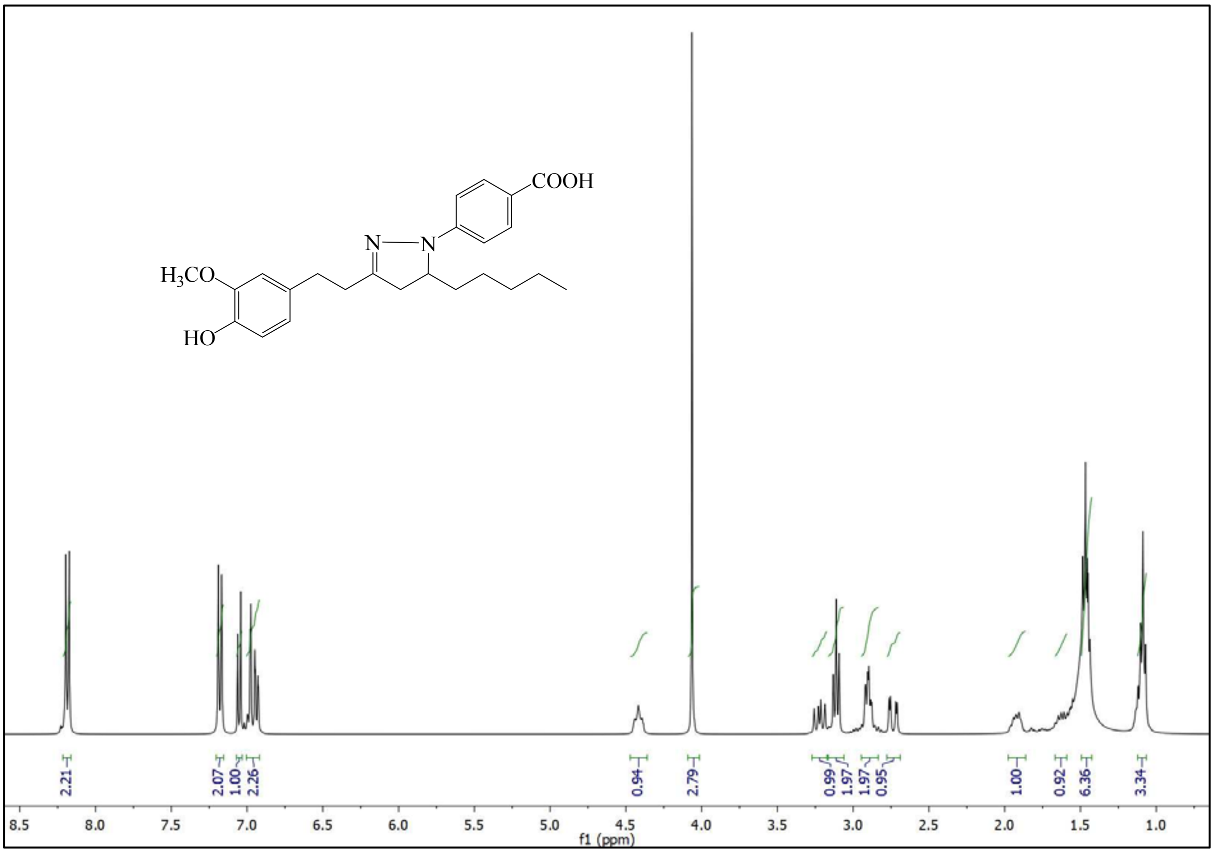

Figure S65. ^{1}H NMR spectrum of **5k**.

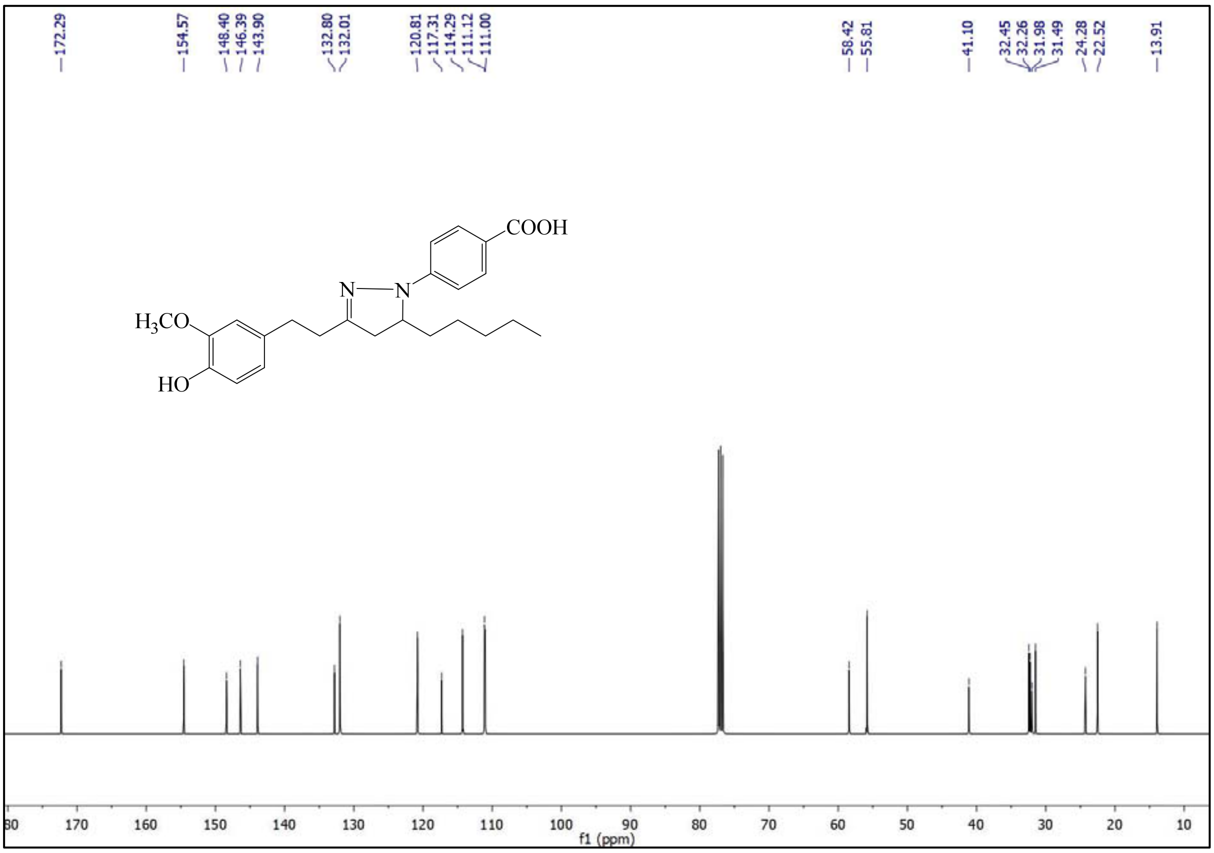

Figure S66. ^{13}C NMR spectrum of **5k**.

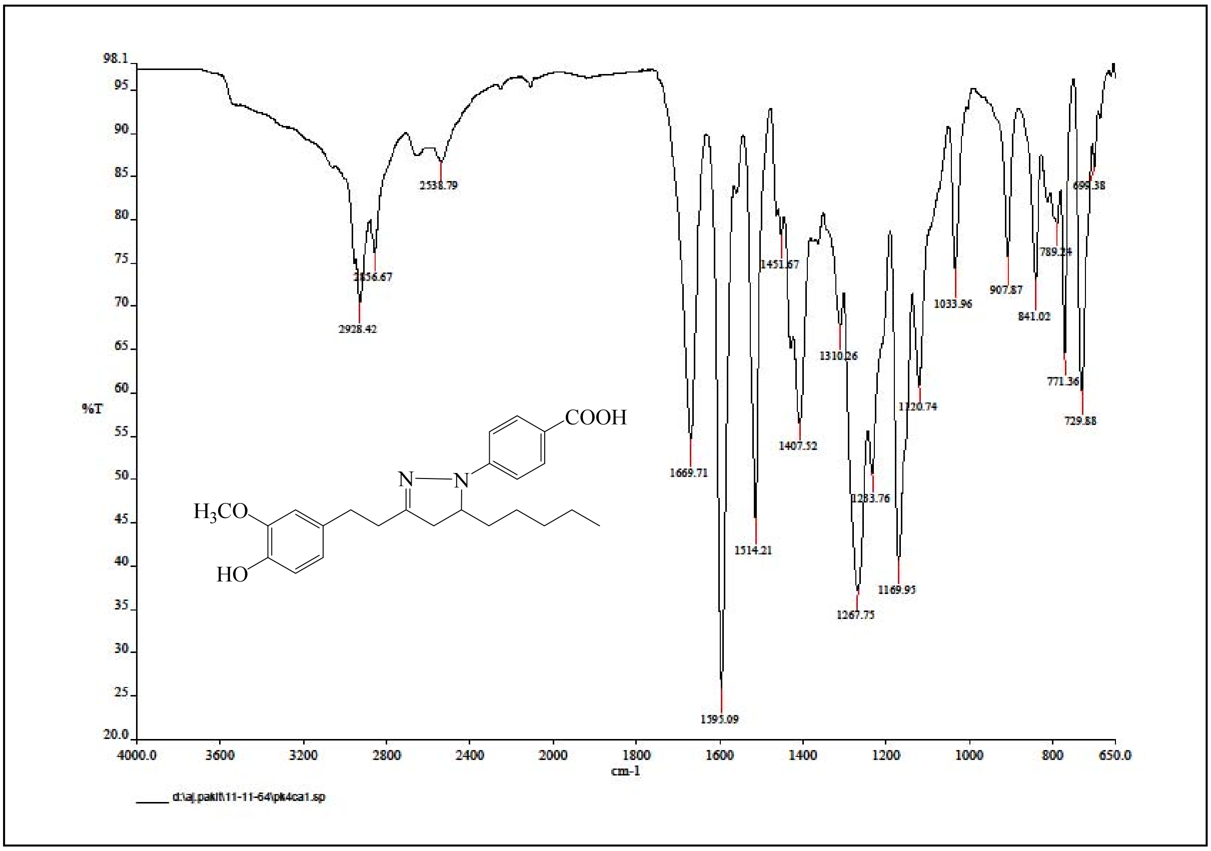

Figure S67. IR spectrum of **5k.**

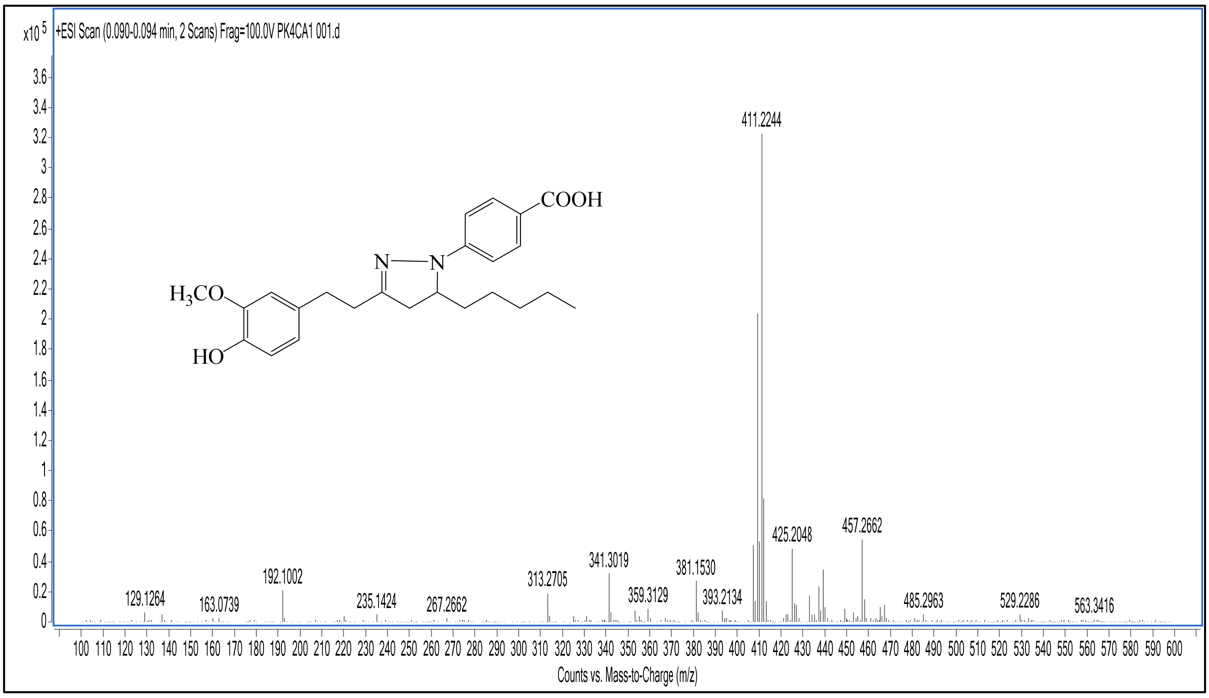

Figure S68. Mass spectrum of **5k**.

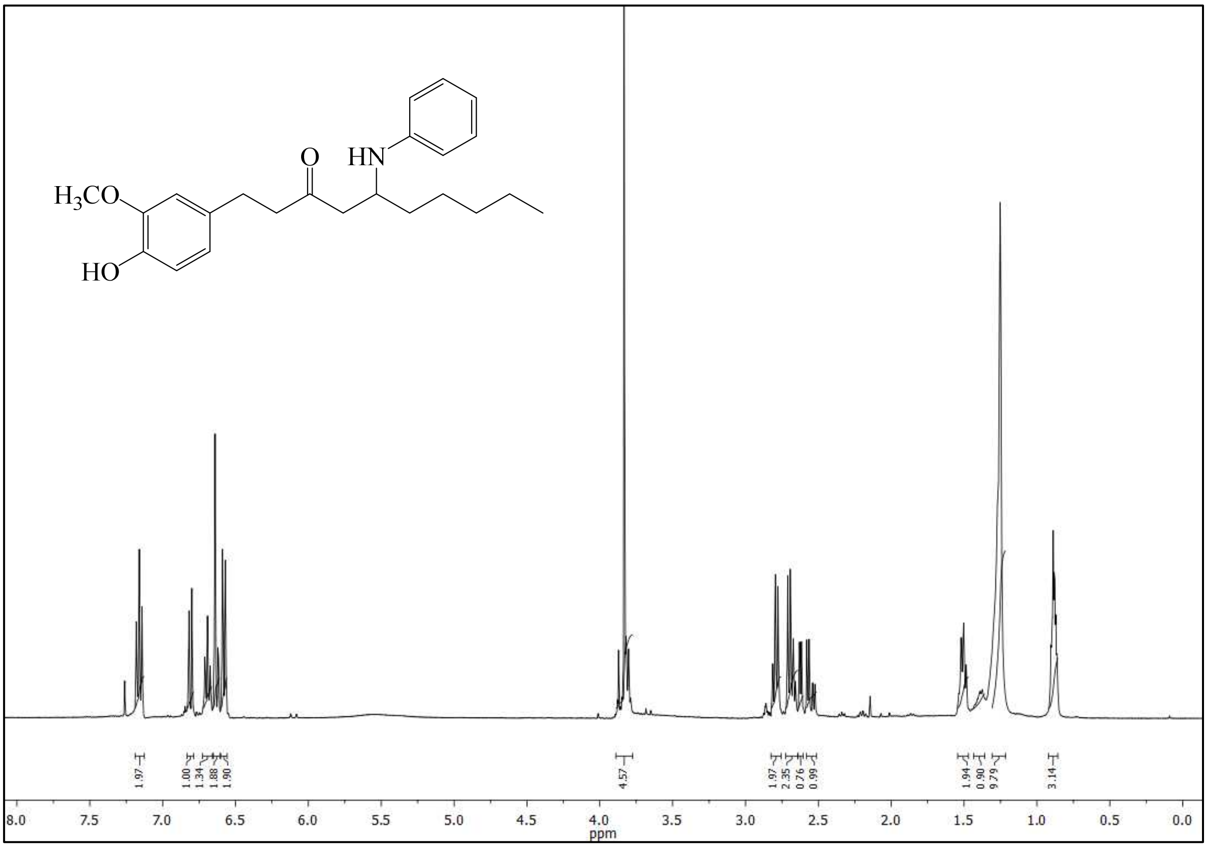

Figure S69. ^{1}H NMR spectrum of **6a**.

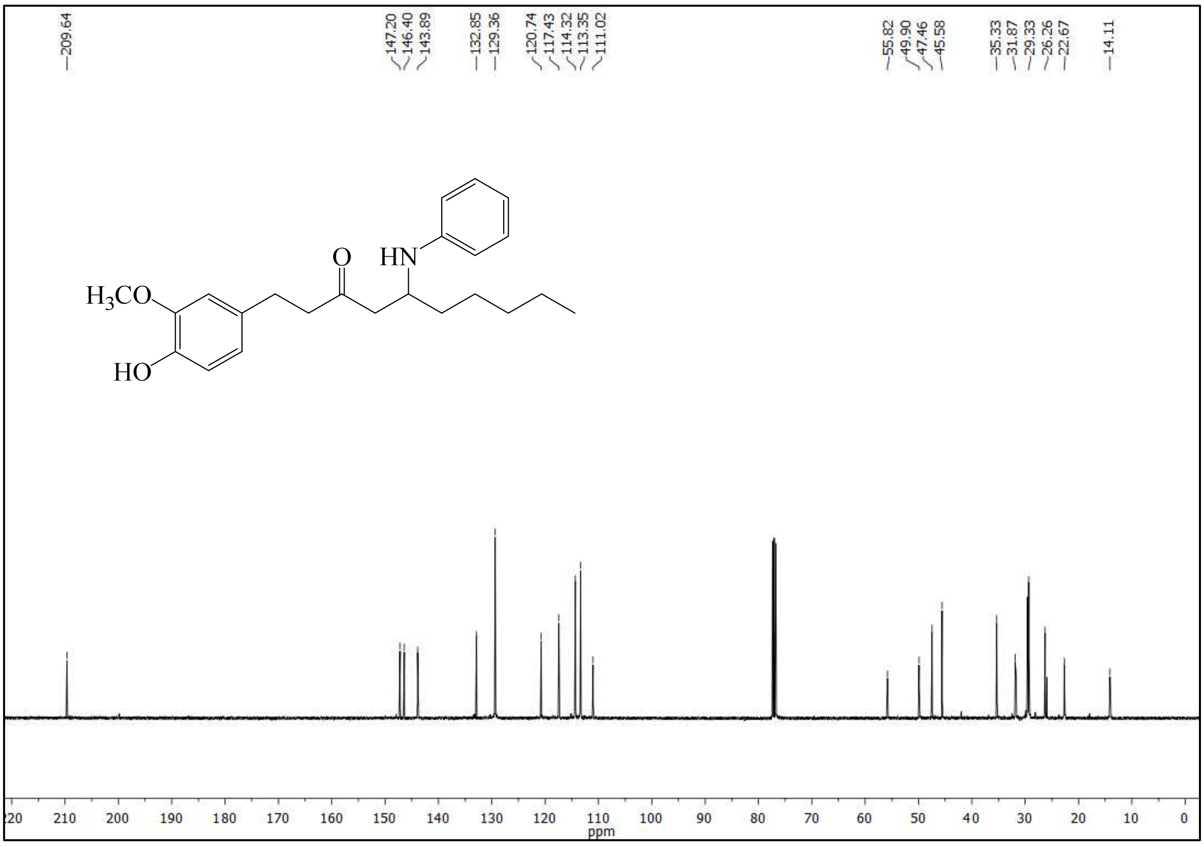

Figure S70. ^{13}C NMR spectrum of **6a**.

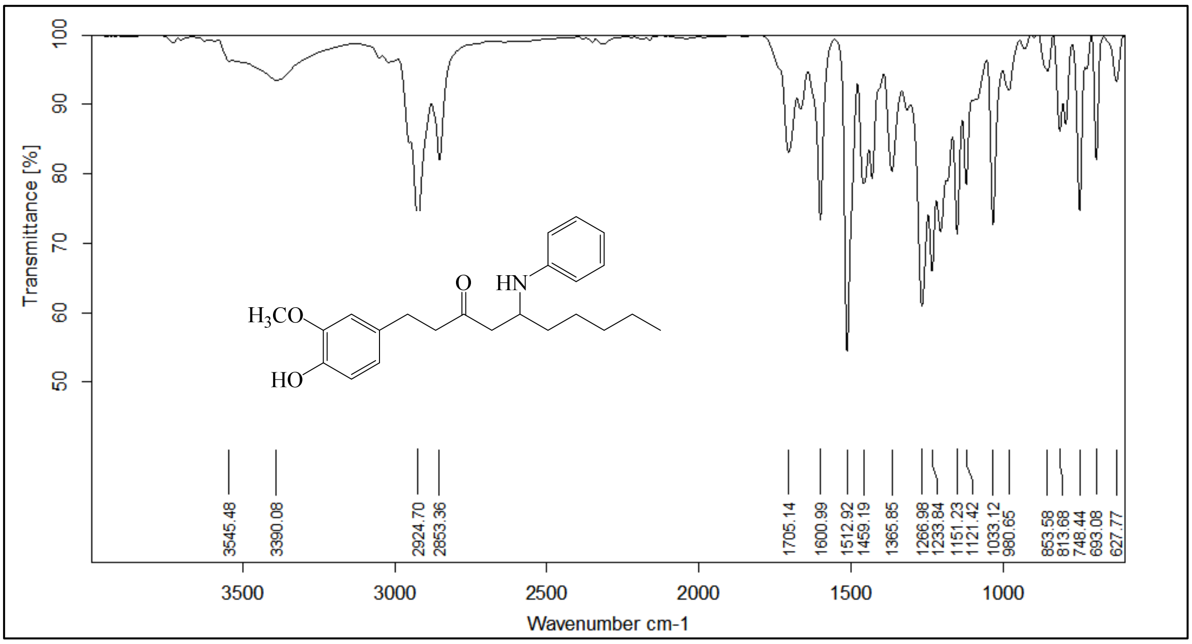

Figure S71. IR spectrum of **6a**.

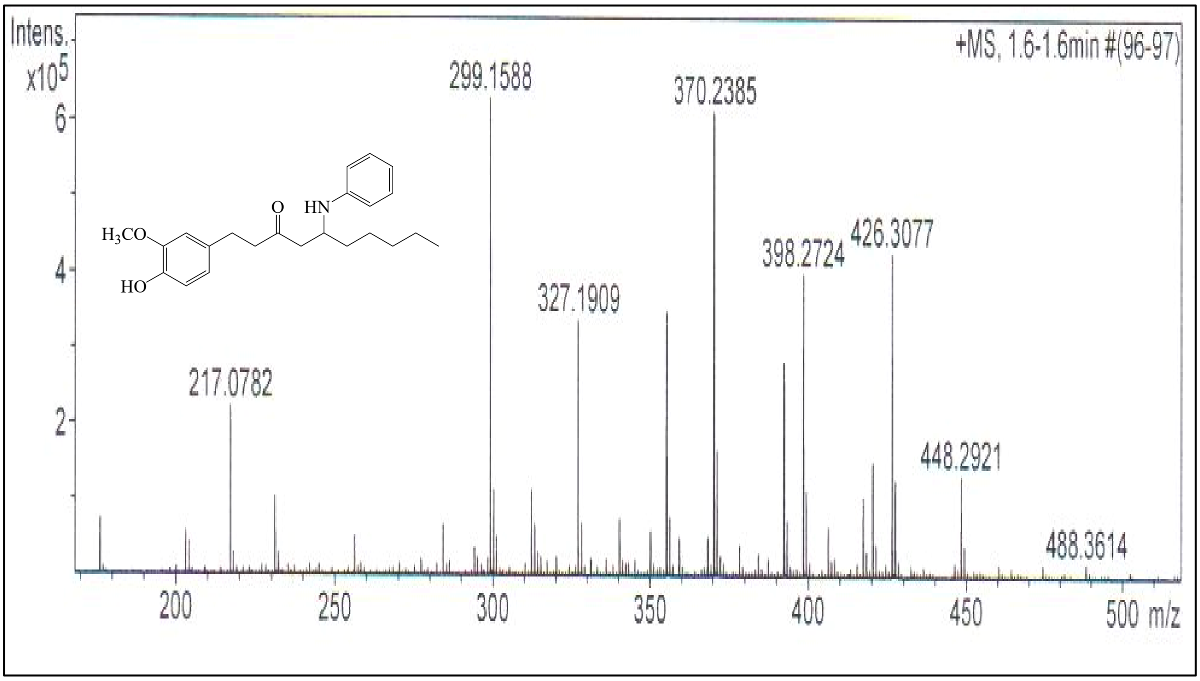

Figure S72. Mass spectrum of **6a**.

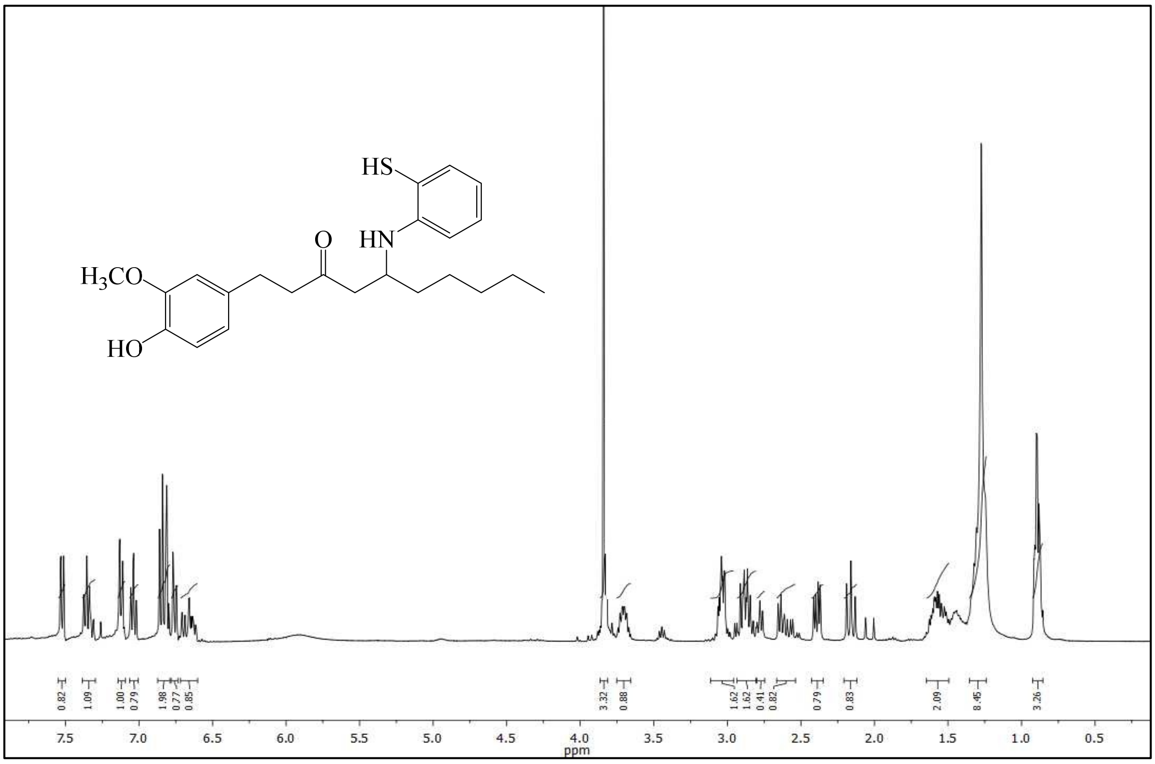

Figure S73. ^{1}H NMR spectrum of **6b**.

#

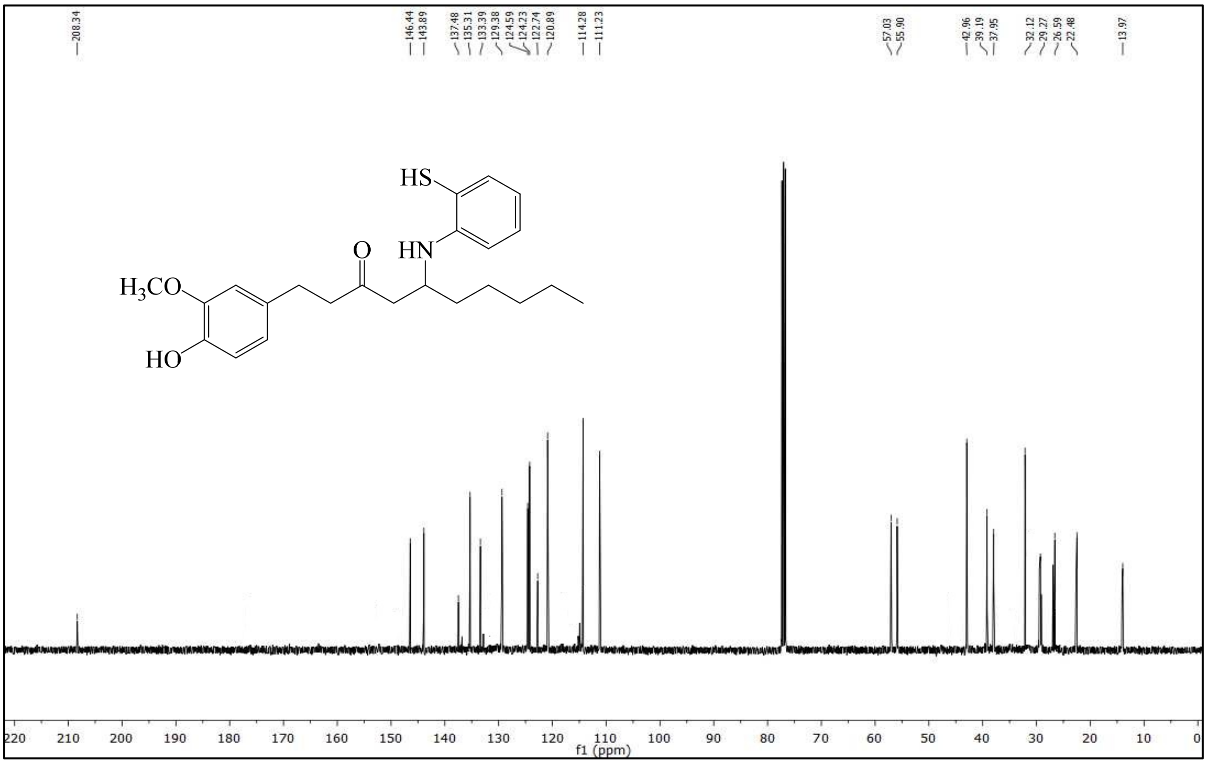

Figure S74. ^{13}C NMR spectrum of **6b**.

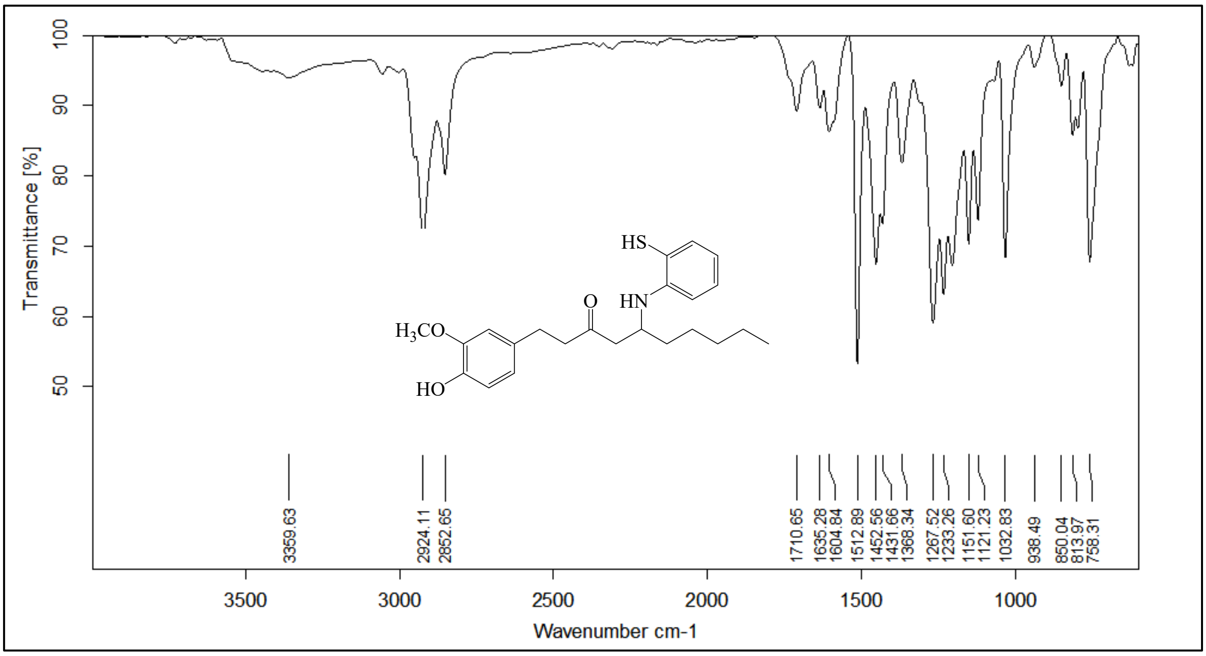

Figure S75. IR spectrum of **6b**.

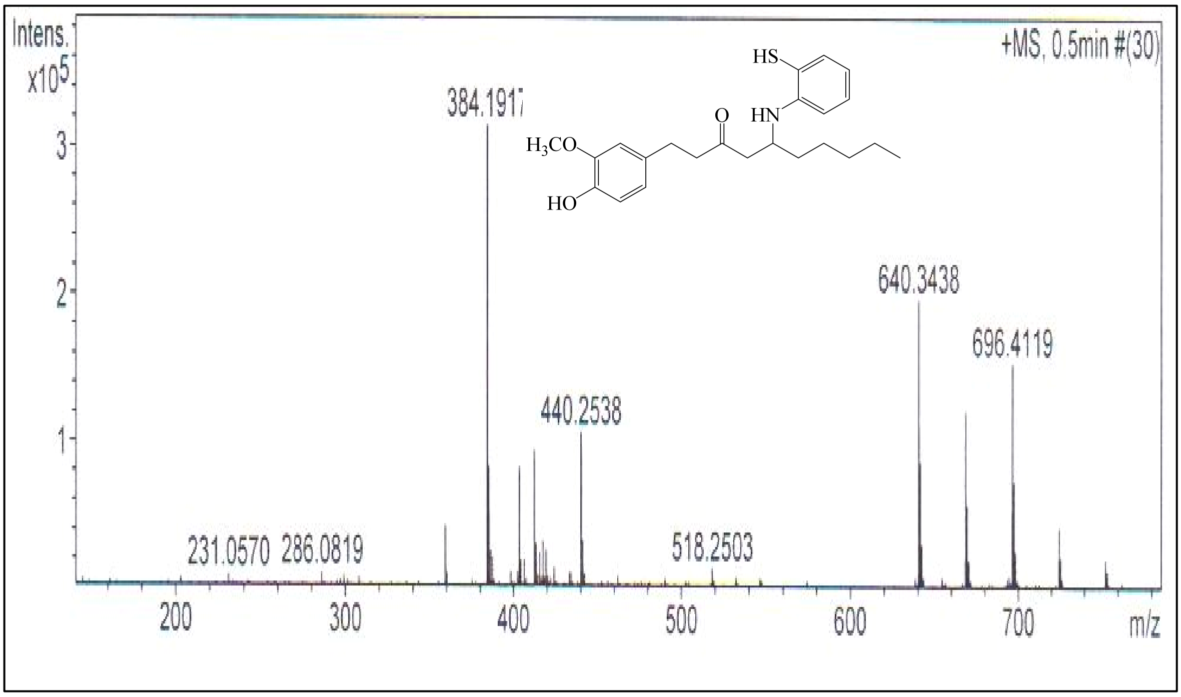

Figure S76. Mass spectrum of **6b**.

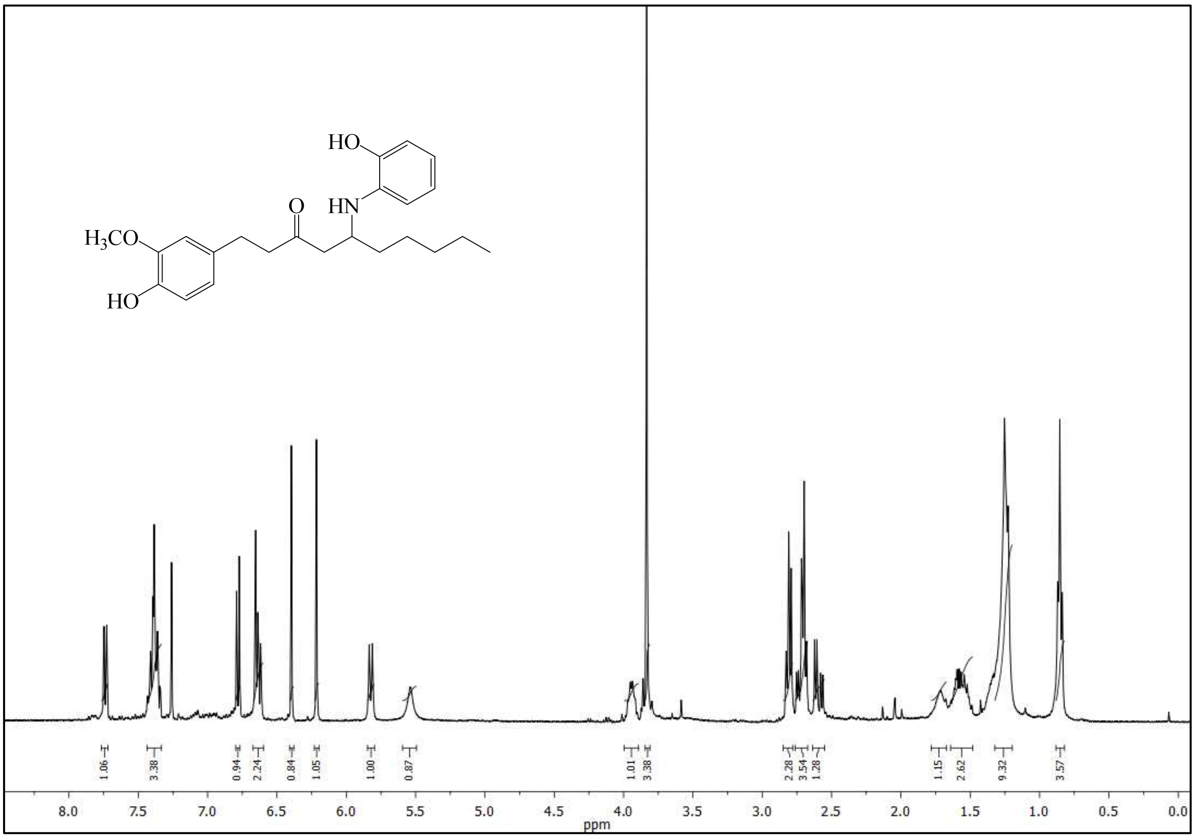

Figure S77. ^{1}H NMR spectrum of **6c**.

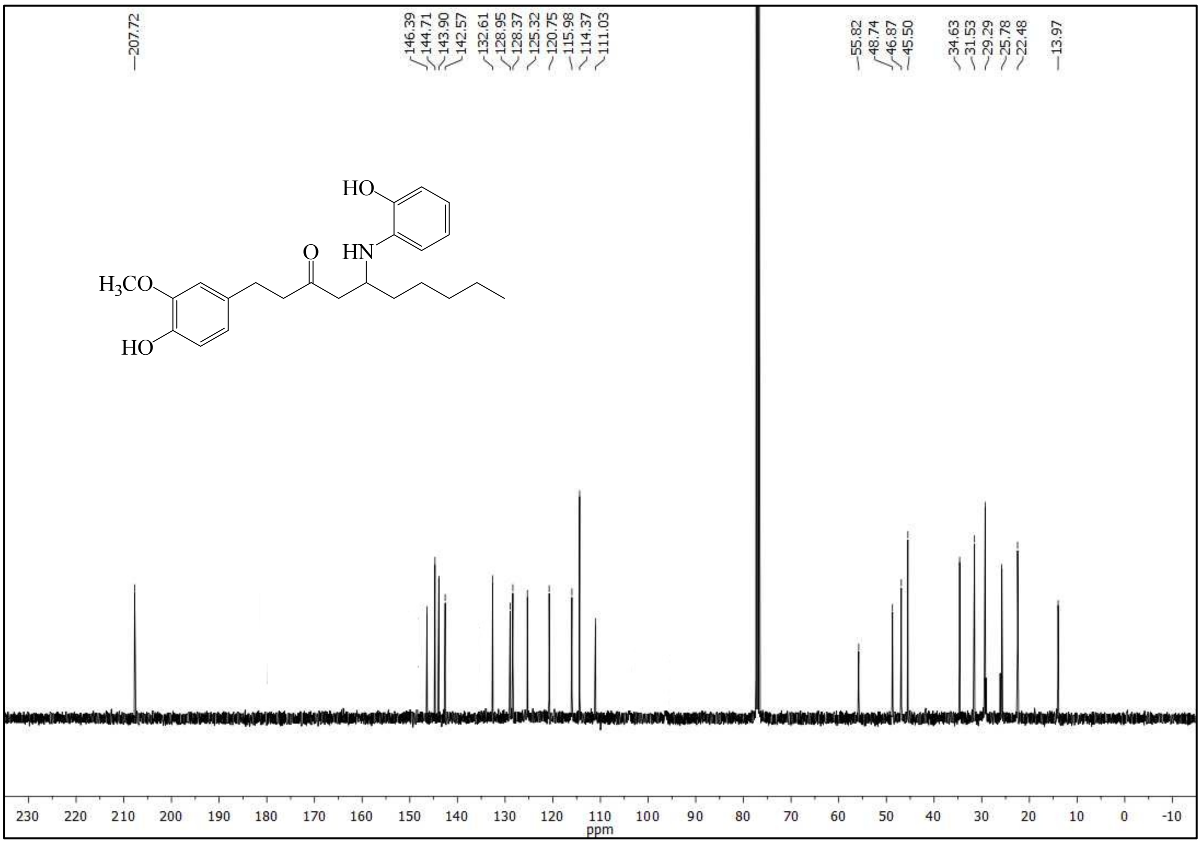

Figure S78. ^{13}C NMR spectrum of **6c**.

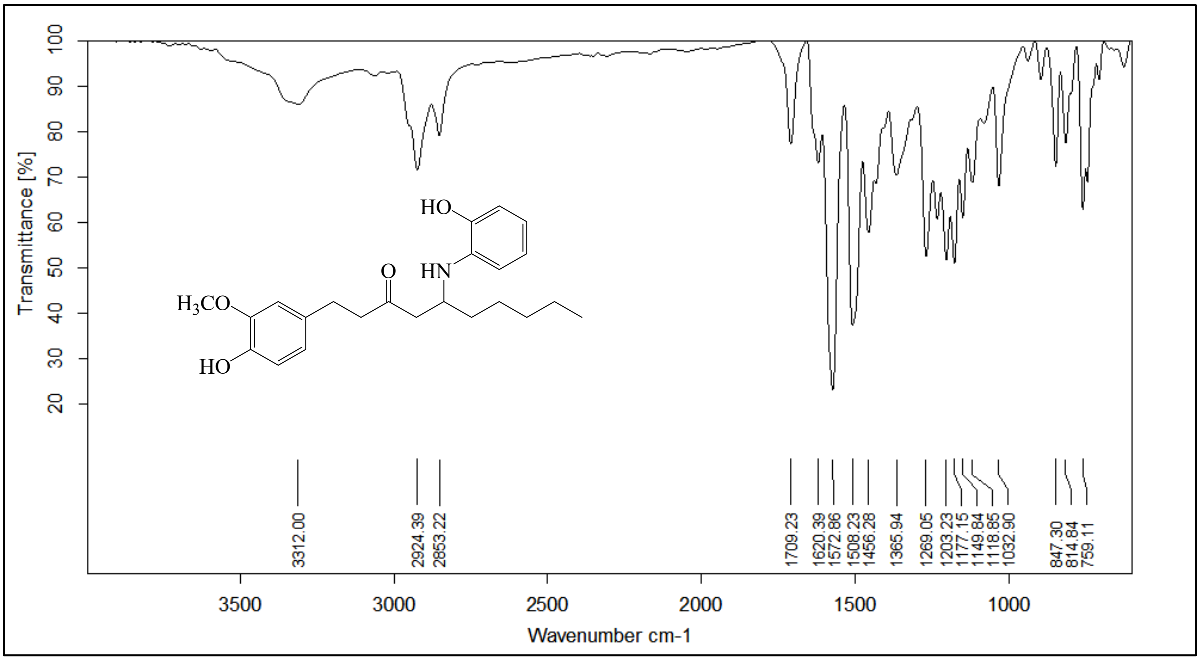

Figure S79. IR spectrum of **6c.**

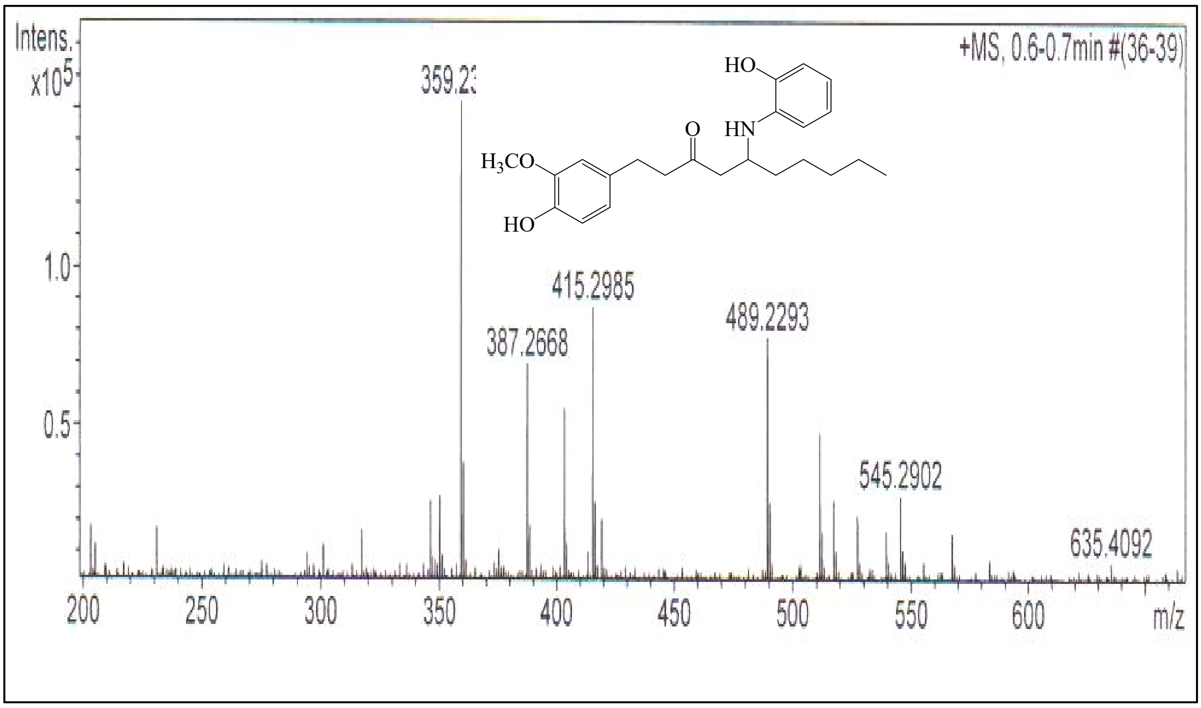

Figure S80. Mass spectrum of **6c.**

#

#

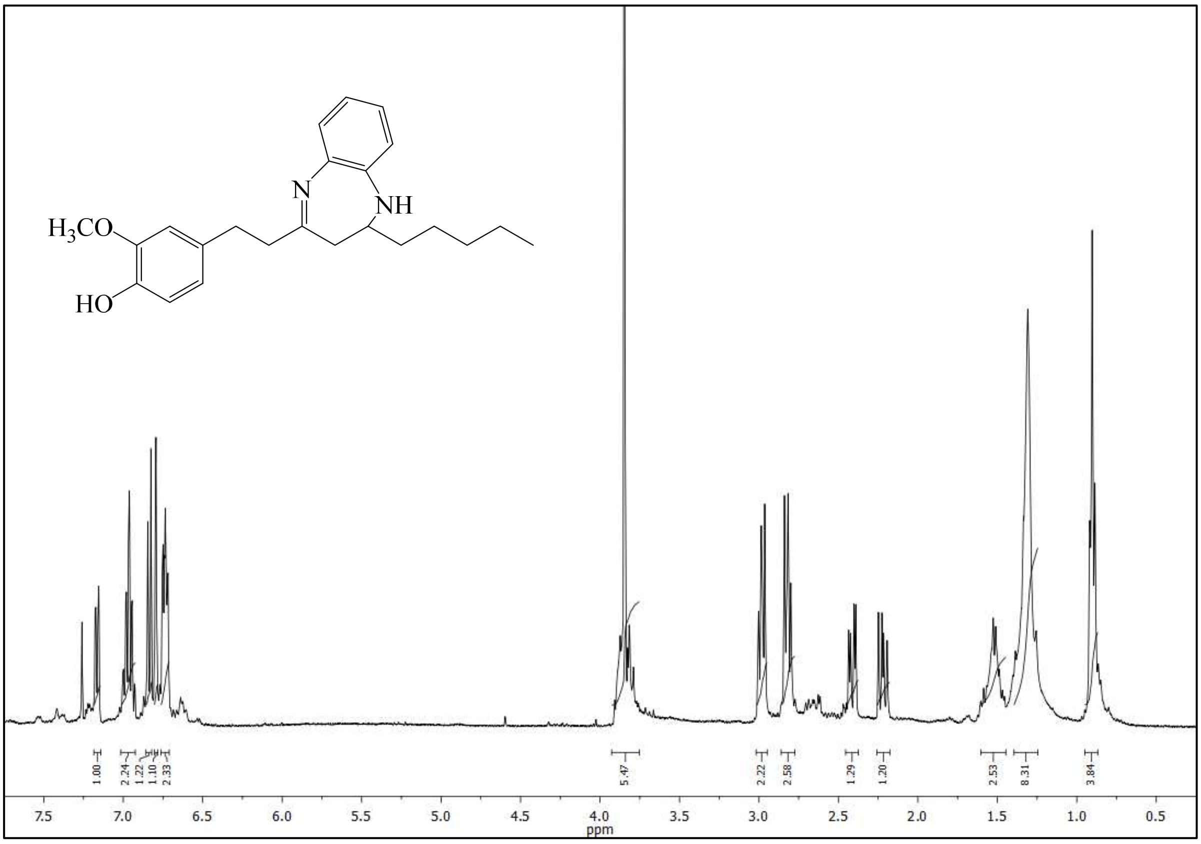

Figure S81. ^{1}H NMR spectrum of **7**.

#

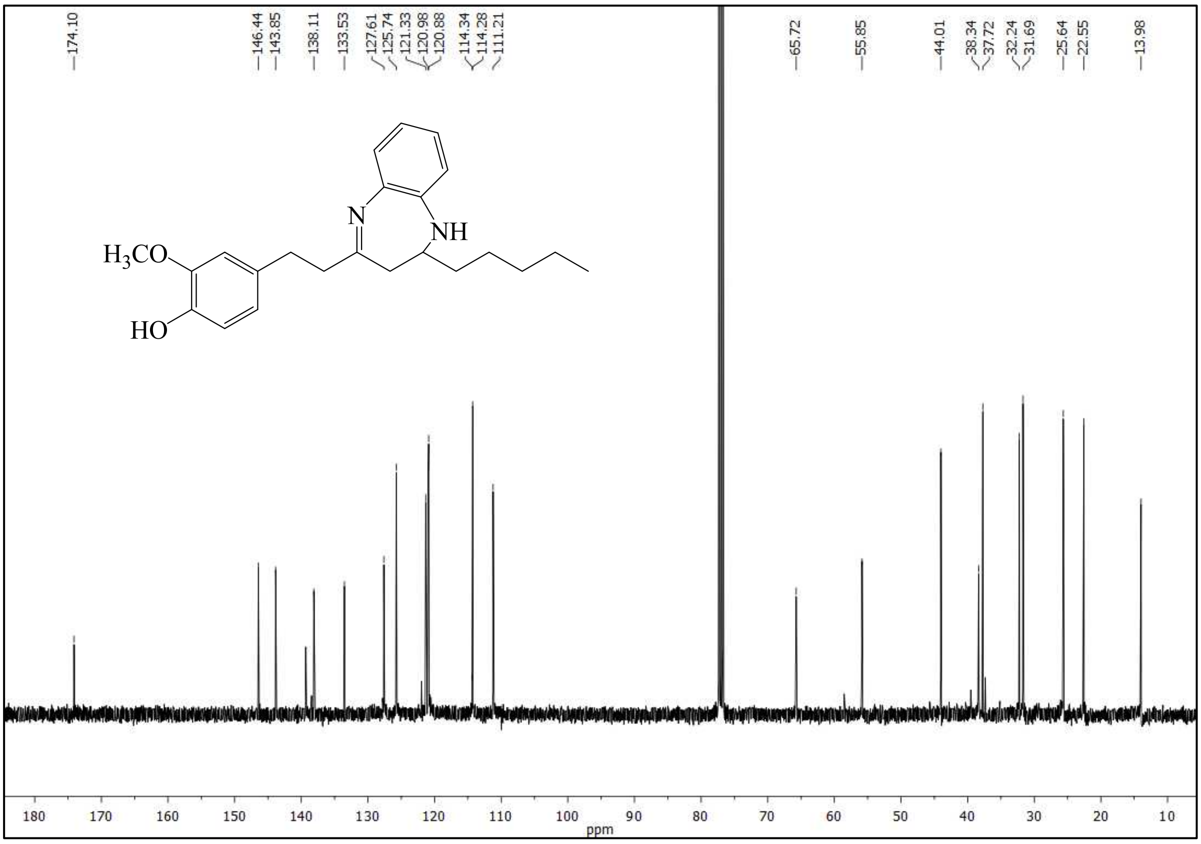

Figure S82. ^{13}C NMR spectrum of **7**.

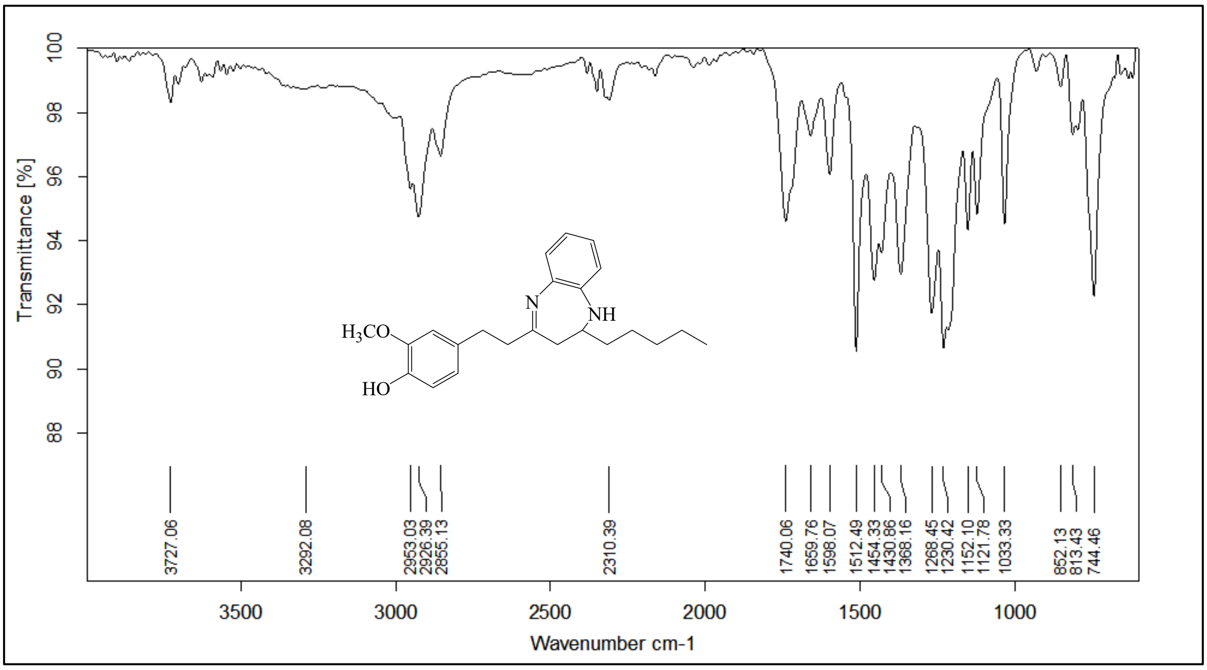

Figure S83. IR spectrum of **7**.

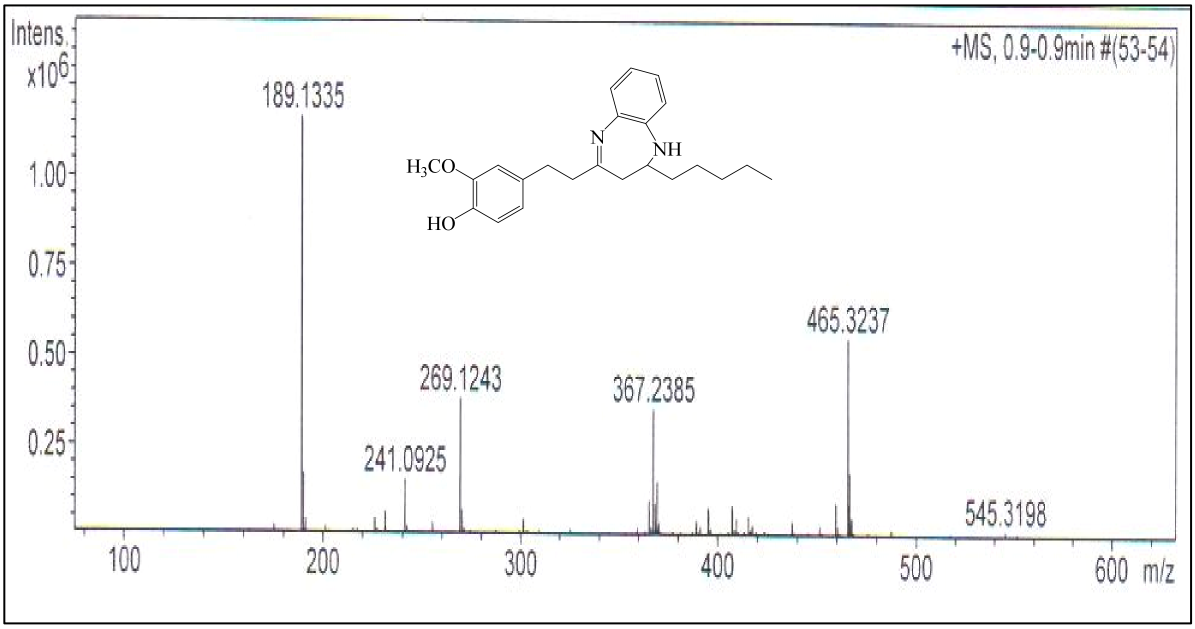

Figure S84. Mass spectrum of **7**.
